# Cardiac Macrophages and Fibroblasts Modulate Atrial Fibrillation Maintenance

**DOI:** 10.1161/CIRCRESAHA.125.326291

**Published:** 2026-02-10

**Authors:** Ana Simon-Chica, Jorge G. Quintanilla, Carlos Torroja, Marinela Couselo-Seijas, Haruka Toda, Peter Lee, Alberto Benguria, Concepción Revilla, Andrés Redondo-Rodríguez, José Manuel Alfonso-Almazán, Alba García-Escolano, Manuel Marina-Breysse, Carlos Galán-Arriola, María Linarejos Vera-Pedrosa, Giulio La Rosa, Ana Dopazo, Fátima Sánchez-Cabo, María Jesús García-Torrent, Adriana Ortega-Hernández, Borja Ibáñez, Estefanía Núñez, Dulcenombre Gómez-Garre, Carlos Morillo, Joachim Greiner, Peter Kohl, Julián Pérez-Villacastín, Nicasio Pérez-Castellano, José Jalife, Javier Domínguez, Jesús Vázquez, Manuel Carnero-Alcázar, David Filgueiras-Rama

**Affiliations:** 1 Novel Arrhythmogenic Mechanisms Program, Centro Nacional de Investigaciones Cardiovasculares (CNIC), Madrid, Spain (A.S.-C., J.G.Q., M.C.-S., H.T., A.R.-R., J.M.A.-A., A.G.-E., M.M.-B., G.L.R., C.M., D.F.-R.).; 2 Centro de Investigación Biomédica en Red de Enfermedades Cardiovasculares (CIBERCV), Madrid, Spain (J.G.Q., M.C.-S., A.R.-R., M.M.-B., A.D., F.S.-C., B.I., E.N., D.G.-G., J.P.-V., N.P.-C., J.J., J.V., D.F.-R.).; 3 Cardiovascular Institute, Instituto de Investigación Sanitaria del Hospital Clínico San Carlos (IdISSC), Madrid, Spain (J.G.Q., J.P.-V., N.P.-C., D.F.-R.).; 4 Bioinformatics Unit, Centro Nacional de Investigaciones Cardiovasculares (CNIC), Madrid, Spain (C.T., F.S.-C.).; 5 Essel Research and Development Inc., Toronto, Canada (P.L.).; 6 Genomics Unit, Centro Nacional de Investigaciones Cardiovasculares (CNIC), Madrid, Spain (A.B., A.D.).; 7 Departamento de Biotecnología, Centro Nacional Instituto de Investigación y Tecnología Agraria y Alimentaria (INIA-CSIC), Madrid, Spain (C.R., J.D.).; 8 Myocardial Homeostasis and Cardiac Injury Program, Centro Nacional de Investigaciones Cardiovasculares (CNIC), Madrid, Spain (C.G.-A., B.I.).; 9 Cardiovascular Regeneration Program, Centro Nacional de Investigaciones Cardiovasculares (CNIC), Madrid, Spain (M.L.V.-P., J.J.).; 10 Department of Medicine, Universidad Complutense de Madrid, Spain (M.J.G.-T., J.P.-V).; 11 Instituto de Investigación Sanitaria del Hospital Clínico San Carlos (IdISSC), Laboratorio de Microbiota y Biología Vascular, Madrid, Spain (A.O.-H., D.G.-G.).; 12 Cardiology department, IIS-University Hospital Fundación Jiménez Díaz, Madrid, Spain (B.I.).; 13 Laboratory of Cardiovascular Proteomics, Centro Nacional de Investigaciones Cardiovasculares (CNIC), Madrid, Spain (E.N., J.V.).; 14 Department of Cardiac Sciences, Libin Cardiovascular Institute, Cumming School of Medicine, University of Calgary, AB, Canada (C.M.).; 15 Institute for Experimental Cardiovascular Medicine, University Heart Center Freiburg—Bad Krozingen, Medical Center—University of Freiburg and Faculty of Medicine, University of Freiburg, Germany (J.G., P.K.).; 16 Fundación Interhospitalaria para la Investigación Cardiovascular (FIC), Madrid, Spain (J.P.-V., N.P.-C.).; 17 Cardiac Surgery Department, Hospital Clínico San Carlos, Madrid, Spain (M.C.-A.).

**Keywords:** atrial appendage, atrial fibrillation, fibroblasts, macrophages

## Abstract

**BACKGROUND:**

Nonmyocytes may contribute to regional adaptive changes during persistent atrial fibrillation (PsAF), favoring its perpetuation. We aimed to investigate the differential features of fibroblast and macrophage populations within individual-specific atrial regions associated with PsAF maintenance.

**METHODS:**

The study was conducted in 2 pig models of PsAF with and without infarct-related substrate (N=27 and N=27, respectively) and further validated in humans with PsAF (N=20). Sham-operated pigs (N=9), healthy animals (N=4), and patients in sinus rhythm (N=7) were used as comparative controls. In pigs, in vivo high-density instantaneous frequency modulation maps were used to identify atrial regions associated with PsAF maintenance (drivers). Regional cellular composition and phenotypic states of fibroblast and myeloid lineages were determined using flow cytometry, single-cell RNA sequencing, immunohistochemistry, and proteomic analyses. The functional relevance of driver regions was further studied in patients with symptomatic PsAF undergoing ablation. Flow cytometry and single-cell RNA sequencing analyses were performed in tissue samples of the left atrial appendage in a complementary cohort of patients with PsAF undergoing thoracoscopic-guided ablation.

**RESULTS:**

PsAF terminated acutely in 12 of 14 pigs undergoing mapping and ablation of driver regions. In humans, driver ablation was associated with 90% AF-freedom (on/off antiarrhythmic drugs) after 2 years of follow-up. Samples from nonablated pigs revealed a phenotypic shift towards ACTA2 (actin alpha 2)-fibroblasts and PTX3 (pentraxin 3)-fibroblasts during PsAF. Although ACTA2-fibroblasts were highly preserved in human samples, paired comparisons in pig samples showed that PTX3-fibroblasts were enriched only in driver regions. PsAF also showed changes in myeloid cells towards inflammatory profiles. However, regional analysis revealed that, in both humans and pigs with PsAF, driver regions were enriched in cardiac resident macrophages with transcriptomic and proteomic profiles favoring cardiomyocyte homeostasis and cell survival.

**CONCLUSIONS:**

PsAF shows differential regional changes in fibroblast and myeloid populations with distinctive gene signatures in areas that drive the overall arrhythmia.

**Novelty and Significance**

**What Is Known?**

- Recruited macrophages, activated myofibroblasts, and some fibroblast states have been linked to fibrosis and atrial fibrillation (AF) pathophysiology.
- Regional analysis of atrial nonmyocyte populations in individual-specific locations associated with long-term AF maintenance (driver regions) remains unexplored.

**What New Information Does This Article Contribute?**

- Nonmyocyte populations during persistent AF show regional adaptive changes within the atria that depend on the functional role of such regions in sustaining the arrhythmia in the long term.
- In pigs with long-lasting persistent AF, fibroblasts show overt compositional shifts with a marked enrichment in PTX3 (pentraxin 3)-fibroblasts within driver regions associated with AF maintenance.
- Recruited inflammatory monocytes emerge in the atria of animals and humans with persistent AF compared with their respective controls. However, driver regions show differential phenotypic changes characterized by an enrichment of resident cardiac macrophages, with homeostatic and anti-apoptotic signatures that may be crucial for sustaining persistent AF dynamics.

Atrial remodeling during persistent AF involves region-specific changes in nonmyocyte populations, which show distinctive gene signatures in areas that drive the overall arrhythmia in the long term. Driver regions are characterized by overt compositional shifts in fibroblasts and myeloid populations, findings that are highly consistent across pig models and humans with persistent AF.

**Meet the First Author, see p e000749**

Persistent and long-standing persistent atrial fibrillation (PsAF) are the most prevalent types of AF (≈75%).^1^ Notwithstanding the associated clinical problems, current therapeutic options fail to provide effective long-term rhythm control in patients with complex substrates.^2,3^ A more careful look at AF progression indicates that atrial remodeling and its modulation may have important implications on the success of rhythm control strategies.^4^ AF itself induces progressive functional and structural changes in the atria that facilitate its perpetuation.^4,5^ Interestingly, AF can be sustained in the long term by the electrical activity located at individual-specific atrial sites, which show faster activation rates than the surrounding tissue.^6,7^ A recent large-scale randomized trial has shown that artificial intelligence-guided ablation of specific atrial regions beyond pulmonary vein isolation increases AF freedom at 1-year follow-up compared with pulmonary vein isolation alone.^8^ These data challenge the assumption that atrial remodeling develops uniformly across the atria.^9,10^ Indeed, regional functional differences in AF dynamics may reflect specific adaptive mechanisms involved in the modulation of homeostatic, functional, and structural changes critical for AF maintenance.

Recent studies have shown that atrial remodeling and AF susceptibility are strongly modulated by innate immunity and inflammatory cells.^11,12^ Hulsmans et al^11^ reported a significant expansion of recruited macrophages in the atria of AF patients using single-cell RNA sequencing (scRNA-seq). The implication of such macrophages in AF susceptibility was confirmed in mice subjected to a macrophage-targeted therapy, which demonstrated a decrease in acute AF inducibility. These data suggest that AF maintenance can be modulated by nonmyocyte cells from the innate immune system. Among these cells, cardiac resident macrophages are the most abundant cells in the heart and have vital organ-specific functions, including extracellular matrix remodeling,^13^ and metabolic homeostasis by clearing debris from mitochondria.^14^ The latter may be essential for cardiomyocytes located in regions associated with AF maintenance, where such cells are exposed to the high metabolic demand associated with long-term high activation rates.^4,7,15^

To date, spatial analysis of atrial nonmyocyte populations and their implications for long-term AF dynamics remains ill-explored. Here, we aimed to investigate the differential regional features of nonmyocyte populations in the atria of clinically relevant pig models and humans with PsAF. We highlight the emergence of cellular states in myeloid cells and fibroblasts associated with PsAF. More importantly, we demonstrate that individual-specific regions, driving the overall arrhythmia, exhibit differential adaptive changes characterized by an enrichment in resident cardiac macrophages and a high abundance of antiapoptotic proteins. The latter may play a critical role in cardiomyocyte homeostasis and survival within driver regions subject to long-term high activation rates.

## Methods

### Data Availability

Raw and processed scRNA-seq data from pigs and humans are available in BioStudies (ArrayExpress)—access no. E-MTAB-14275. All other data supporting the findings in this study are included in the main article and associated source files. The mass spectrometry proteomics have been deposited to the ProteomeXchange Consortium via de PRIDE^16^ partner repository with the data set identifier PXD053398.

### Porcine Model of Long-Lasting Lone Persistent AF

**Figure 1.**
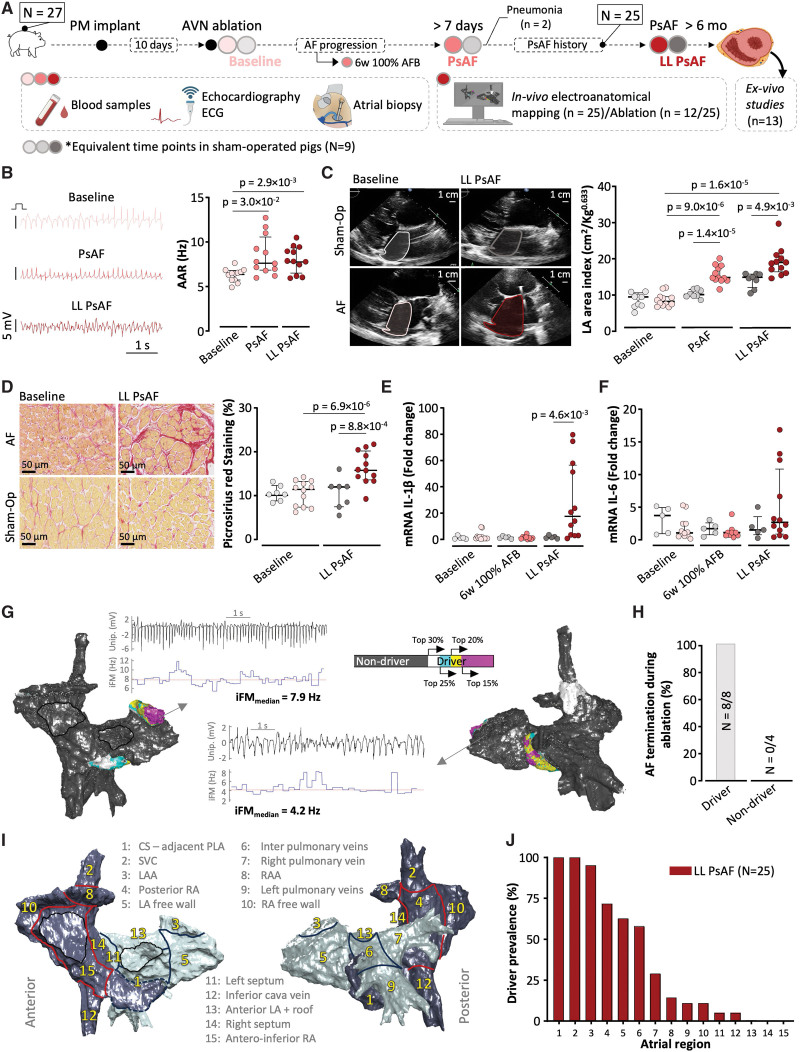
**Phenotypic features of persistent atrial fibrillation (PsAF) in a porcine model without underlying heart disease. A**, Schematic outline of the experimental protocol in pigs with lone PsAF. Red color-graded circles indicate AF animals (N=27) and different follow-up periods. Gray color-graded circles indicate sham-operated controls (N=9) and equivalent follow-up time points based on the average times of AF animals to reach PsAF and long-lasting PsAF (LL PsAF). **B**, **Left**, Representative examples of local bipolar atrial electrograms at baseline, PsAF, and LL PsAF. **Right**, Quantification and comparisons of atrial activation rates (AAR, in Hz) at baseline, PsAF, and LL PsAF (n=12). **C**, **Left**, Sample echocardiography images and left atrial (LA) area delineation in PsAF animals and sham-operated controls at baseline and during LL PsAF (or equivalent follow-up time in controls). **Right**, Quantification and comparisons of LA areas in AF animals and sham-operated controls at baseline, PsAF and LL PsAF (AF n=12/ Sham-Op n=8). **D**, **Left**, Representative micrographs of picrosirius red-stained sections of right atrial biopsies (40×) from PsAF animals and sham-operated controls at baseline and LL PsAF (or equivalent follow-up time in controls). **Right**, quantification and comparisons of interstitial atrial fibrosis in AF animals and sham-operated controls. (AF n=12/Sham-Op n=7). **E** and **F**, Quantification of mRNA expression levels in tissue samples from endocardial right atrial biopsies of IL (interleukin)-1β; (**E**) and IL-6 (**F**) at baseline (AF n=12/Sham-Op n=5), at 6 weeks of 100% AF burden (AFB; AF, n=9/Sham-Op, n=5) and at the time of LL PsAF (or equivalent follow-up time in controls; AF, n=12/ Sham-Op, n=5). **G**, Sample in vivo median instantaneous frequency modulation (iFM_median_) map to identify driver regions in LL PsAF. Driver regions are color-coded according to their hierarchy within the top 30% of iFM_median_ values. Nondriver regions are represented in dark gray. Sample unipolar signals (first row, black tracing) to calculate the iFM signals (second row, blue tracing) and their median values from driver and nondriver regions. **H**, Percentage of successful AF termination after ablation at driver and nondriver regions in pigs (8 and 4, respectively) with lone PsAF. **I**, Anterior and posterior atrial views with regional segmentation. **J**, Driver prevalence location in lone PsAF animals (N=25). The one-way ANOVA with Tukey posthoc test was used to assess statistical significance in **B**. The two-way ANOVA was used in **C** and **D**, followed by the Šídák (unpaired groups) test for multiple comparisons. In **E** and **F**, the multiple Mann-Whitney *U* test was used with the Holm-Šídák correction for multiple testing. **A** created with BioRender software (https://www.biorender.com). AVN indicates atrioventricular node; LAA, left atrial appendage; CS, coronary sinus; PM, pacemaker; PLA, posterior left atrium; RA/A, right atrium/atrial appendage; and SVC, superior vena cava.

Yucatan-Large White crossbred pigs were used to generate a lone PsAF model (N=27). Two of 27 animals died during the follow-up due to intercurrent pneumonia (Figure 1A). A group of sham-operated controls was used for comparison (N=9). A dual-chamber pacemaker was surgically implanted with intracardial leads positioned in the right atrial appendage and the right ventricle. Ten days after pacemaker implantation, a second procedure was performed to ablate the atrio-ventricular node. Then, an atrial pacing protocol was initiated to induce AF using a 30-second burst pacing at 20 Hz, followed by a 6-second sensing period. Sinus rhythm detection during the sensing period automatically triggered a repeat of the high-rate atrial pacing to reinduce AF. AF history was classified as follows: (1) baseline paroxysmal AF episodes, detected early after atrio-ventricular node ablation, (2) PsAF, when self-sustained AF episodes lasted >7 days, and (3) long-lasting PsAF, when episodes lasted >6 months. The same procedures were performed in sham-operated controls, but the high-rate atrial pacing protocol was not activated, and the pacemaker was programmed in DDDR mode.

### Porcine Model of Long-Lasting Persistent AF With Underlying Infarct-Related Substrate

Yucatan-Large White crossbred pigs underwent 3 hours of ischemia via balloon-expansion in the proximal left circumflex artery, followed by 1 hour of continuous monitoring during reperfusion, similar to other ischemia-reperfusion models reported elsewhere (N=27).^17^ Refractory ventricular fibrillation occurred in 2 animals, which died during the procedure. A further 2 pigs died during the 2-month subacute period after the infarction and before the initiation of the AF protocol. Eight weeks after the infarction, the surviving animals (n=19) underwent the same procedures and protocol as animals without myocardial infarction (MI) to induce PsAF (MI-PsAF). Four of these animals died during the AF protocol due to sudden cardiac death. Electrophysiological changes associated with the infarct substrate were analyzed in the left atrium of isolated Langendorff-perfused heart preparations. Optical imaging of transmembrane voltage changes was performed in 4 hearts with infarction and 4 healthy controls.

### High-Density In Vivo Electroanatomical Mapping in Pigs

All PsAF animals, with and without infarct-related substrate (MI-PsAF [n=15] and lone PsAF [n=25], respectively), underwent in vivo high-density electroanatomical mapping at the end of the follow-up period to identify animal-specific atrial regions associated with AF maintenance (ie, driver regions). Driver regions were defined as those activating faster than their surroundings and within the top 30% of median instantaneous frequency modulation values in the atria, as reported elsewhere.^7^ A subset of animals underwent catheter-based ablation (8 with lone PsAF and 6 with MI-PsAF). Radiofrequency applications aimed to ablate all driver regions by creating coin-like sets of lesions, unless a specific region was located in high-risk areas for catheter-based ablation (e.g., left and right atrial appendages). Ablation was stopped after conversion to sinus rhythm, complete elimination of all leading-driver locations, or after 50 minutes of radiofrequency energy delivery, even if not all driver sites had been ablated. Four additional animals with PsAF underwent ablation of nondriver locations to investigate the relevance of drivers for AF maintenance.

### Ethics of Animal Protocols

All animal procedures were approved by the Comunidad de Madrid (Ref no. PROEX097/17 & PROEX078.8/21) and conformed to the regulations outlined in EU Directive 2010/63EU and Recommendation 2007/526/EC regarding the protection of animals used for experimental and other scientific purposes. Extended details of the animal models, anesthesia, follow-up studies, invasive mapping, and ablation procedures are described in the Supplemental Material. A schematic chart with all animal groups is shown in Figure S1. Specific animal characteristics are provided in the Supplemental Material and Tables S1 through S4.

### Clinical Studies in Patients

An initial prospective pilot study was conducted in patients (N=10) with symptomatic PsAF refractory to at least 1 antiarrhythmic drug, and a history of at least 1 ablation procedure, including pulmonary vein isolation. The study included consecutive patients from January 2018 to June 2021 who were considered for a redo ablation procedure according to current recommendations.^18^ The invasive mapping procedure aimed to identify specific atrial regions potentially associated with AF maintenance based on instantaneous frequency modulation maps. Driver regions were defined using the same criteria as in pig models, and the ablation protocol followed the strategy used in animals with PsAF. In cases with incomplete ablation of driver regions, the patients were invited to undergo a thoracoscopic-guided procedure to ablate the remaining drivers and isolate the left atrial appendage (LAA), both electrically and mechanically, if detected as an AF driver during the mapping procedure. All patients underwent clinical follow-up to assess recurrences at 1, 3, 6, 12, 18, and 24 months after the procedure.

A second prospective study was conducted in patients ≤75 years old with symptomatic PsAF, refractory to antiarrhythmic drugs, and with a history of at least 2 ablation procedures. The patients were recruited from March 2022 to May 2025. All patients underwent the interventional mapping and ablation procedure described in the pilot series to identify driver regions. After the percutaneous procedure, we selected a subgroup of patients who required a thoracoscopic-guided procedure to complete the ablation of all driver regions, provided that the LAA was one of the driver locations (N=10). LAA samples were collected after its mechanical and electrical isolation.

Finally, a third prospective series was conducted in patients without a previous history of AF undergoing open-chest cardiac surgery for coronary artery disease or ascending aortic aneurysm (N=7). Only patients ≤75 years old and without overt signs of structural cardiac damage were included. Patients were recruited from April 2022 to May 2025. LAA samples were collected at the time of surgery.

LAA samples were used for flow cytometry and scRNA-seq analysis. All patients were recruited at the Cardiovascular Institute of the Hospital Clínico San Carlos (Madrid, Spain). The ethics committee of the institution approved each one of the clinical series, and all patients provided written informed consent. Extended details are described in the Supplemental Material.

### Euthanasia and Atrial Tissue Collection in Pigs

At the end of the follow-up and after invasive mapping procedures, all animals with PsAF underwent open-chest cardiac surgery for heart excision and euthanasia. Tissue samples from driver and nondriver regions were used for histopathology and immunohistochemistry, real-time quantitative polymerase chain reaction, microRNA analysis, quantitative high-throughput proteomics, and immunoblotting studies. Fresh atrial tissue samples were harvested and enzymatically digested for nonmyocyte cell isolation (see Supplemental Material for details).

### Single-Cell Preparation and CD45^+^ Enrichment

Viable and nucleated (Sytox Blue^−^ DRAQ5^+^) endothelial cells (CD31^+^ CD45^−^), leukocytes (CD45^+^), and other nonmyocytes (CD31^−^ CD45^−^) were sorted using the fluorescence-activated cell sorting Aria II cell sorter (BD Biosciences). Details are described in the Supplemental Material.

### Chromium 10X Library Preparation and Analysis of ScRNA-Seq Data

ScRNA-seq experiments were performed at the genomics unit of the CNIC. ScRNA-seq libraries were prepared using the Chromium 10× Genomics platform and sequenced on a NextSeq 2000 (Illumina). Data were processed with the CellRanger v6.1.1 pipeline and analyzed in R using Seurat 4.0.5 for clustering and downstream analyses. As part of the differential expression analyses, raw counts were aggregated per gene and sample to generate pseudo-bulk profiles and analyzed with DESeq2. To capture regional effects, the DESeq2 design matrix included condition (sham-operated, PsAF, and MI-PsAF), anatomic region (driver and nondriver), and their interaction. This model (1) controls for baseline regional transcriptional differences observed in sham-operated tissue and (2) identifies genes whose driver versus nondriver differences are specifically modified in PsAF or MI-PsAF relative to sham-operated samples. Multiple-testing correction was performed using the Benjamini–Hochberg method (α=0.05). Details are described in the Supplemental Material.

### Analysis of Cytokines in Plasma and Atrial Tissue Samples of Pig Models

Quantification of IL (interleukin)-1β, IL-6, and TNF (tumor necrosis factor)-α was performed in plasma samples and tissue homogenates using an immunoassay with an ad hoc personalized MILLIPLEX MAP Porcine Cytokine/Chemokine Magnetic Bead Panel (PCYTMAG-23K, Merck Millipore, Darmstadt, Germany). The analysis was conducted using a Luminex 200 system.

### Statistical Analyses

All data are presented as median and interquartile range unless otherwise stated. Normal data distribution was assessed with the Shapiro-Wilk test. For variables showing a normal distribution, statistical significance between 2 groups was evaluated using a 2-sided paired or unpaired Student *t* test, as appropriate. For comparisons among more than 2 groups, a one-way ANOVA followed by the Tukey posthoc test, or a two-way ANOVA followed by the Šídák multiple comparisons test was used, as appropriate. For variables without a normal distribution, the Mann-Whitney *U* test or the Wilcoxon signed-rank test (paired samples) was used for 2-group comparisons, and the multiple Mann–Whitney *U* tests with Holm–Šídák correction were applied for multiple comparisons. For categorical variables, the Pearson χ^2^ test was used to assess differences when the expected frequencies were ≥5; otherwise, the Fisher exact test was applied. Statistical significance for 2-group comparisons in proteomic data was assessed using Limma. A *P*<0.05 was considered statistically significant. Statistical analysis was performed using GraphPad Prism version 9.0.0 (GraphPad Software, San Diego, CA) or Stata/IC 15.1.

## Results

### Atrial Remodeling After Long-Lasting Persistent AF Shows Overt Signs of Local Inflammation

Pigs without infarct-related substrate developed PsAF within 5.20 months (2.97–8.50) of initiation of the high-rate atrial pacing protocol. Sham-operated controls were assessed at matching follow-up times (Figure 1A). AF progression showed an increase in atrial activation rates from baseline paroxysmal AF episodes to PsAF (6.5 Hz [5.7–6.7] versus 7.7 Hz [6.9–9.7], respectively; *P*=0.003), without further significant changes during the follow-up (Figure 1B). AF was associated with progressive atrial dilation from baseline to PsAF and long-lasting PsAF. Left atrial dilation was significantly larger in AF animals than in sham-operated controls (Figure 1C). Right atrial biopsies at baseline and at the time of long-lasting PsAF also showed a significant increase in interstitial atrial fibrosis (11.8% [7.8–11.8] versus 15.6% [13.8–19.2], respectively; *P*<0.001), which was not documented in sham-operated controls (Figure 1D). Gene expression analysis of key proinflammatory cytokines in atrial biopsies showed upregulation of IL-1β and TNF-α at the end of the follow-up compared with sham-operated controls (IL-1β, 21.7-fold versus control, *P*=0.005; TNF-α, 7.16-fold versus control, *P*=0.04; and IL-6, 2.67-fold versus control, *P*=0.63; Figure 1E and 1F; Figure S2A). At the time of long-lasting PsAF, plasma samples from the coronary sinus confirmed significantly higher concentrations of IL-1β and TNF-α in PsAF animals compared with sham-operated controls (Figure S2B). No differences between PsAF and controls were documented in peripheral blood samples (Figure S2C). The latter suggests local atrial inflammatory changes in pigs with long-lasting lone PsAF rather than systemic inflammation.

### Individual-Specific Atrial Regions Can Sustain Persistent AF

In vivo high-density instantaneous frequency modulation maps (4541 points [3580–5837]) were used to identify driver regions at the end of the follow-up in all pigs with lone PsAF (N=25). Mapping procedures were performed after a median of 7.77 months (4.83–9.00) in self-sustained PsAF (Figure 1G). In a subset of 8 animals with lone PsAF, catheter-based ablation of driver locations terminated AF after 35.8 minutes (17.2–44.2) of radiofrequency energy application (Figure 1H). Driver ablation also rendered AF episodes nonsustained (lasting <10 minutes) after ≥3 reinduction attempts (a sample case is shown in Figure S3). Conversely, ablation at nondriver locations failed to terminate PsAF in any of the 4 cases within the per-protocol maximum of 50 minutes of radiofrequency energy application (Figure 1H; Figure S4). In 13 pigs, driver regions were identified but not ablated to investigate the differential cellular and molecular makeup of these regions compared with nondriver areas. One of these 13 pigs had undergone driver ablation in a first procedure (Figure S3). Then, high-rate atrial pacing was reinitiated the day after ablation to allow for the progressive development of a new PsAF episode. The second mapping procedure, performed 63 days after the index ablation, identified a new driver location which was left unablated to allow for scRNA-seq analyses after euthanasia. Overall, mapping data identified 77 drivers with a median of 4 driver regions (3–4 driver regions) per pig. Driver regions were predominantly located in the coronary sinus and its adjacent tissue on the posterior left atrium, the superior vena cava, and the LAA (Figure 1I and 1J).

### Persistent AF Is Characterized by the Emergence of 2 Fibroblast Clusters

**Figure 2.**
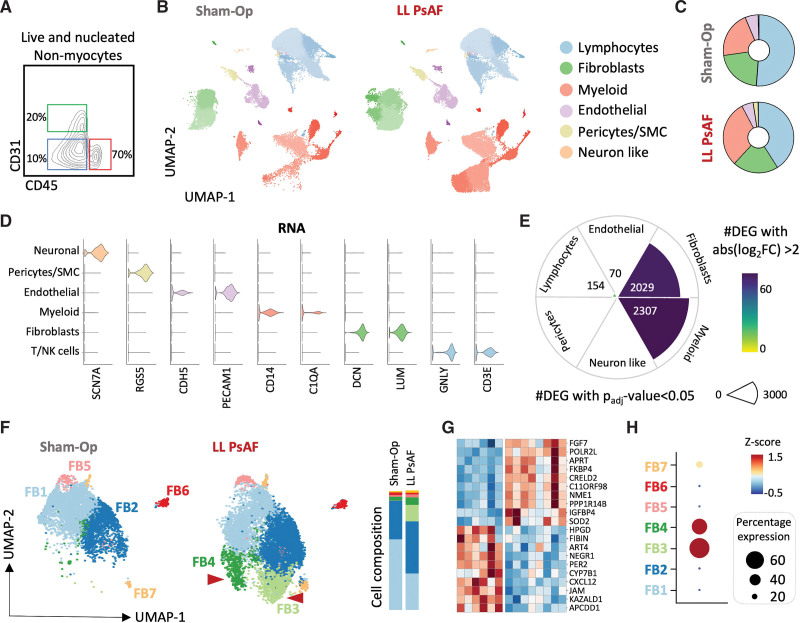
**Lone persistent atrial fibrillation (PsAF) is characterized by the expansion of 2 emergent fibroblast clusters. A**, Isolated viable cells were enriched for CD45^+^ (70%). Abundant cell populations such as CD31^+^ endothelial cells and other nonmyocytes (CD45^−^CD31^−^) were limited to 20% and 10%, respectively. **B**, Single-cell RNA sequencing (scRNA-seq) and unsupervised clustering identified 6 major cell populations based on differential expression of canonical markers in atrial specimens of animals with long-lasting lone PsAF (LL PsAF, n=4) and sham-operated controls (n=3). Atrial specimens included samples from both atria: the right atrial free wall, the coronary sinus with its adjacent posterior left atrium wall, and the superior vena cava (2–3 independent regions were included per animal, yielding a total of 9 and 6 samples for LL PsAF and sham-operated controls, respectively). Data are displayed as uniform manifold approximation and projection (UMAP) plots, where each dot represents a single cell. Distinct cell clusters are color-coded. **C**, Distribution of cell populations per model. Color coding corresponds to the colors from UMAP in **B**. **D**, Violin plots displaying characteristic marker genes of each major cell population in **B**. **E**, Number of differentially expressed genes (DEG; *P*_adj_ <0.05) with an absolute log_2_-fold change (FC) >2 per major cell population in LL PsAF compared with sham-operated animals. Pie size indicates the number of DEG with *P*_adj_ <0.05. The pie size is color-coded based on the number of DEG with log_2_-FC >2 (blue: high; yellow: low). **F**, High-resolution clustering identifies up to 7 cardiac fibroblast (FB1–FB7) clusters in LL PsAF animals and sham-operated controls with equivalent follow-up times. Red arrowheads indicate 2 emergent fibroblast clusters in LL PsAF. The bar graph shows the proportion of each cluster in sham-operated and LL PsAF samples. **G**, Heat map displaying the top upregulated and downregulated DEG ranked by log_2_-FC when comparing LL PsAF and sham-operated samples. DEGs were derived from pseudo-bulk (DESeq2) analysis. **H**, Z-score dot plot showing the expression of upregulated genes from **G**. NK indicates natural killer; and SMC, smooth muscle cells.

ScRNA-seq analysis was performed in atrial samples (n=9) from driver and nondriver regions of 4 pigs with long-lasting lone PsAF (Table S1). Six additional samples from anatomically matched tissue of potential driver and nondriver regions were harvested from 3 sham-operated pigs. Relative cell numbers were limited to prevent oversampling of abundant cell populations, such as fibroblasts or endothelial cells (Figure 2A). The integrated data set comprised a total of 76 072 cells, which represented a wide array of cell types, including fibroblasts, pericytes, neuronal cells, endothelial cells, myeloid cells, and other immune cell populations (T cells and natural killer cells; Figure 2B through 2D; Figure S5). Cell abundance examination revealed a 1.5-fold larger myeloid cluster in the atria of animals with PsAF compared with sham-operated controls (Figure 2C). Pseudo-bulk analysis showed that fibroblasts and myeloid cells had the largest number of differentially expressed genes (|log_2_-fold change|>2, adjusted *P*<0.05, Figure 2E) in the atria of PsAF animals compared with controls.

Higher-resolution clustering revealed 2 fibroblast clusters (FB3 and FB4) that were predominantly present in samples from animals with PsAF (Figure 2F; Figure S6A and S6B). FB3 and FB4 exhibited unique transcriptional signatures, comprising 321 and 220 differentially expressed genes, respectively, while sharing 561 overlapping differentially expressed genes (Figure S6C and S6D). Pseudo-bulk analysis of aggregated cells from the same individual across experimental conditions identified a marked upregulation of *SOD2* (superoxide dismutase-2), *TIMP1* (tissue inhibitor of metalloproteinases 1), and *NME1* (nucleoside diphosphate kinase 1) in PsAF samples compared with sham-operated controls (Figure 2G). Upregulated genes were concentrated in FB3 and FB4 clusters (Figure 2H). Gene set enrichment analysis^19^ using Hallmark and Kyoto Encyclopedia of Genes and Genomes MSigDB gene sets showed enrichment in proteasome, oxidative phosphorylation, MYC targets V1 and glycolysis processes (see also data set no. 1). Based on their distinctive transcriptional profiles, FB3 and FB4 were defined as FB3-PTX3 (pentraxin 3 positive) and FB4-ACTA2 (actin alpha-2 positive), respectively (Figure S6C and S6D). Immunostaining confirmed significantly higher levels of α-SMA (ACTA2) and PTX3 in PsAF samples compared with sham-operated controls (Figure S6E and S6F).

### Persistent AF Shows Overt Shifts in Macrophage Composition and Monocytes Favoring Inflammatory Populations

Differential expression analysis in myeloid cells revealed an upregulation of genes associated with inflammation and monocyte recruitment (*ARG1* [arginase 1], *VCAN* [versican], or *S100A8*, *S100A12* [S100 calcium binding protein A8 and A12]) in PsAF samples compared with sham-operated controls (see also data set no. 2). In contrast, genes associated with antigen presentation functions (*HLA-DRA*, *SLA-DQ1*, *CD74*) and attenuation of macrophage activation (*PLAC8*) were downregulated in myeloid clusters during PsAF.

**Figure 3.**
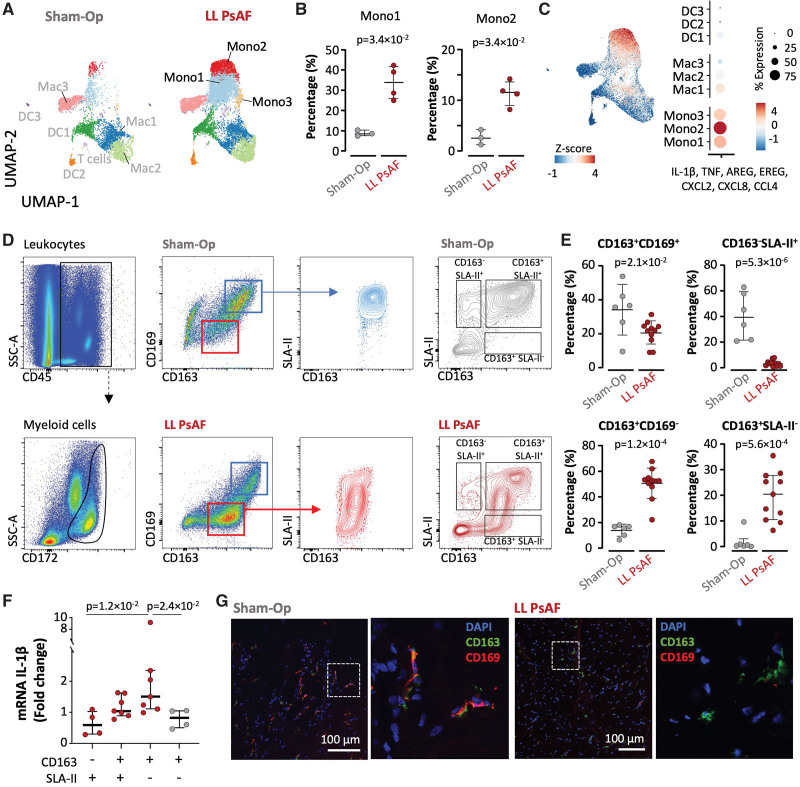
**Single-cell RNA sequencing (scRNA-seq) reveals distinguishing clusters of myeloid cells associated with lone persistent atrial fibrillation (PsAF). A**, High-resolution unsupervised clustering of immune cells identified 9 distinct myeloid cell clusters based on differential expression analysis in atrial specimens (from both atria, as indicated in Figure 2) from animals with long-lasting lone PsAF (LL PsAF) and sham-operated controls. Data are displayed as a uniform manifold approximation and projection (UMAP) plot where each dot represents a single cell and clusters are color-coded. **B**, Examination of cell proportions revealed a higher presence of monocyte clusters (Mono1, Mono2) in LL PsAF specimens compared with sham-operated samples. The Mann-Whitney *U* test was used to assess differences. **C**, **Left**, Z-score feature plot showing the expression of inflammatory genes on the UMAP projection. **Right**, Dot plot representing the Z-score of the inflammatory gene expression signature per macrophage, monocyte, and dendritic cell cluster. **D**, **Left**, representative gating strategy for flow cytometry analyses of myeloid cell populations in right atrial samples from LL PsAF animals and sham-operated controls. Live cells were gated, doublets excluded, and CD45^+^CD172^hi^ cells identified. CD172^hi^ cells were further classified into 2 distinct populations according to their relative expression of CD163, CD169, and SLA-II markers. **E**, Quantification of the myeloid cell populations identified in **D** for sham-operated controls (**top** row) and LL PsAF animals (**bottom** row; AF n=11/Sham-Op n=6). The 2-sided unpaired Student *t* test and the Mann-Whitney *U* test were used to assess differences, as appropriate. **F**, mRNA expression of IL (interleukin)-1β in the populations identified in **D**. The Mann-Whitney *U* test was used for comparisons. **G**, Representative immunofluorescence images of CD163^+^ and CD169^+^ myeloid populations in right atrial samples from sham-operated controls and LL PsAF animals. Original magnification ×20; scale bars are as indicated, including zoomed-in details (white boxes).

Examination of myeloid cells revealed 3 macrophage clusters, 3 monocyte clusters, and 3 clusters of dendritic cells (Figure 3A; Figure S7A through S7C). Cell abundance analysis showed an increase in 2 monocyte clusters (Mono1 and Mono2) in PsAF animals compared with sham-operated controls (Figure 3B). Mono1 and Mono2 clusters expressed high levels of inflammatory mediators (Figure 3C). The monocyte cluster (Mono3) expressing lipid metabolism markers (*SPP1* [secreted phosphoprotein 1], *TREM2* [triggering receptor expressed on myeloid cells 2], and *APOE* [apolipoprotein E]) resembled a recently described population known to secrete osteopontin (encoded by *SPP1*), a matricellular signaling protein implicated in the promotion of fibrosis and AF pathophysiology.^11^ The Mac2 cluster displayed a transcriptional profile enriched in tissue-resident macrophage markers (*TIMD4* [T-cell immunoglobulin and mucin domain containing 4], *LYVE1* [lymphatic vessel endothelial hyaluronan receptor 1], and *FOLR2* [folate receptor 2]; Figure S7D).^20^

Flow cytometry analysis showed 2 different populations based on their relative membrane expression of CD163 (associated with monocytes and macrophage heterogeneity in pigs)^21^ and CD169 (expressed by distinct populations of tissue macrophages but not monocytes)^22,23^ (Figure 3D). The analysis was performed after gating live myeloid cells and doublet exclusion (CD45^+^CD172^hi^). The atria of PsAF animals showed a significantly higher proportion of CD163^+^CD169^-^ cells compared with sham-operated controls (49.9±3.6% versus 13.2±1.9%, respectively; *P*<0.0001; Figure 3E), consistent with the increase in Mono1 and Mono2 clusters documented in the scRNA-seq analysis (Figure 3B and 3C). Furthermore, CD163^+^CD169^+^ cells displayed increased forward scatter and side scatter properties, and high SLA-II expression, whereas CD163^+^CD169^−^ cells expressed varying intensities of SLA-II, indicating additional heterogeneity (Figure 3D; Figure S7E). Interestingly, CD163^+^SLA-II^−^ cells were significantly more frequent in PsAF samples compared with sham-operated controls. Conversely, CD163^−^SLA-II^+^ cells were significantly more frequent in controls compared with PsAF (Figure 3E). These differences were associated with higher expression of IL-1β in CD163^+^SLA-II^−^ cells compared with CD163^−^SLA-II^+^ cells (Figure 3F), which was consistent with the higher IL-1β expression in monocyte clusters (Figure S7F). Moreover, immunofluorescence staining confirmed the presence of CD163^+^CD169^+^ cells in atrial samples from sham-operated controls and CD163^+^CD169^−^ cells in PsAF samples (Figure 3G). These results indicate an increase in proinflammatory monocytes in the atria of long-lasting PsAF animals compared with sham-operated controls.

### Fibroblasts and Macrophages Show Distinctive Transcriptional Hallmarks in Driver Regions of Pigs With Lone PsAF

**Figure 4.**
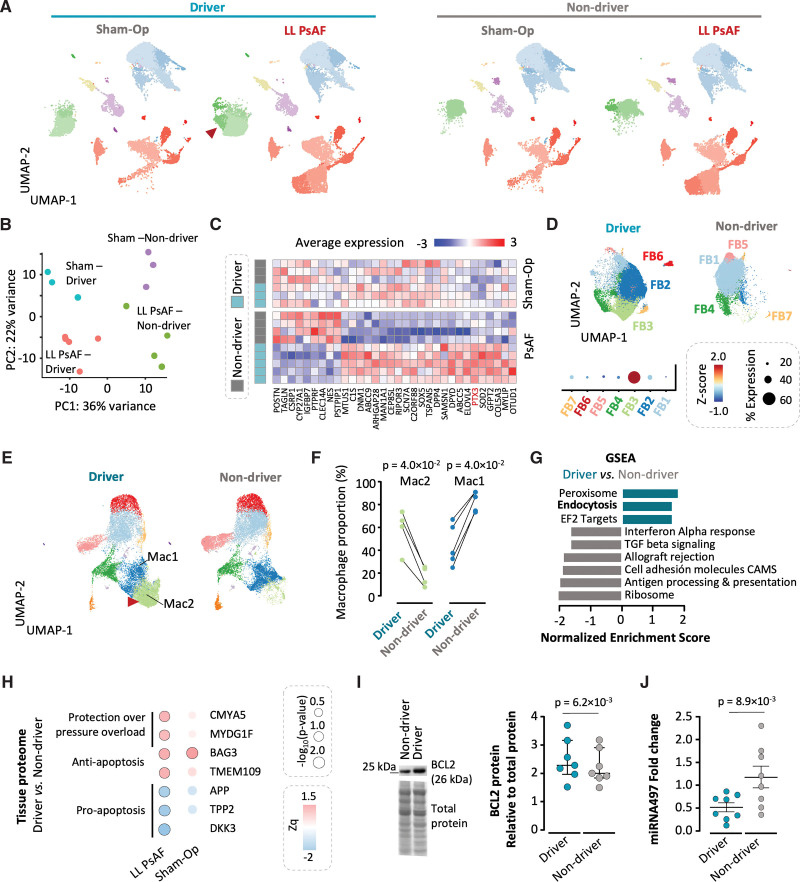
**Persistent atrial fibrillation (PsAF) shows regional phenotypic shifts in cardiac fibroblasts and macrophages. A**, Uniform manifold approximation and projection (UMAP) plots of driver and nondriver regions from animals with long-lasting lone persistent atrial fibrillation (LL PsAF) and sham-operated controls. **B**, Principal component analysis (PCA) plot of pseudo-bulk single-cell RNA sequencing (scRNA-seq) data from fibroblasts colored by cardiac condition (sham-operated or LL PsAF) and region (driver or nondriver). Potentially driver and nondriver regions in sham-operated animals were selected based on the functional relevance (driver prevalence) of equivalent anatomic regions in LL PsAF animals. **C**, Heatmap of differentially expressed genes from pseudo-bulk data for fibroblast populations between driver and nondriver regions in LL PsAF samples and sham-operated controls. **D**, **Top**, high-resolution unsupervised clustering of fibroblasts from driver and nondriver atrial regions of animals with LL PsAF. Bottom, dot plot showing the Z-score per fibroblast cluster of upregulated genes in driver regions of the LL PsAF samples presented in **C**. **E**, High-resolution unsupervised clustering of immune cells from driver and nondriver atrial regions of animals with LL PsAF. **F**, The Mac2 cluster, expressing a protective tissue resident signature (see also Figure S12), was more predominant in driver regions, whereas the opposite was true for the Mac1 cluster (driver regions n=5/ nondriver regions n=4; more than 1 driver region was included in a pig with LL PsAF). **G**, Gene set enrichment analysis (GSEA; hallmarks and Kyoto Encyclopedia of Genes and Genomes [KEGG]) showing upregulated pathways on genes ranked by log_2_-FC between driver and nondriver regions of animals with LL PsAF. **H**, Heatmap of the atrial tissue proteome from driver and nondriver regions of animals with LL PsAF and sham-operated controls. Zq values are shown on the color-coded scale. Point size reflects statistical significance, based on the −log_10_ (*P* value), and significant proteins are outlined in black. **I**, **Left**, representative Western blot of Bcl-2 (B-cell leukemia/lymphoma 2) expression in driver and nondriver regions of a pig with LL PsAF. The full Western blot is avaliable in the Supplemental Material (page 52). **Right**, quantification and comparison of Bcl-2 expression between driver and nondriver regions of animals with LL PsAF (n=7). **J**, Quantification and comparison of miRNA-497-5p levels from driver and nondriver regions of animals with LL PsAF (n=8). The Wilcoxon signed-rank test was used to assess differences in **F**, the 2-sided paired Student *t* test was used in **I** and **J**, and the Limma test was applied in **H**.

The analysis of nonmyocyte populations in the atria as a whole is not sufficient to identify adaptive regional changes, which could be crucial for the functional relevance of driver regions. Unsupervised clustering revealed distinct cell populations, with relative abundances that varied between driver and nondriver regions (Figure 4A). Pseudo-bulk analysis comparing these regions showed that fibroblasts exhibited the largest number of differentially expressed genes. Principal component analysis further revealed that both the experimental group (long-lasting lone PsAF versus sham-operated) and the functional region (driver versus nondriver) were the dominant sources of transcriptional variance in fibroblasts (Figure 4B). More specific gene expression analysis demonstrated adaptive changes in driver regions from PsAF animals that were not observed in the corresponding anatomic locations of sham-operated controls (Figure 4C). Reclustering of fibroblasts in PsAF samples showed a redistribution of subpopulations, where FB2, FB3, and FB6 clusters predominantly localized within driver regions, while FB1 was enriched in nondriver regions (Figure 4D). Notably, upregulated genes within driver regions (Figure 4C) were concentrated in the FB3-PTX3 cluster (Figure 4D). Individual replicates showed consistent patterns of cell-type distribution within each experimental group (Figures S8–S11).

Regional analysis in atrial samples from PsAF animals demonstrated that the Mac2 cluster, expressing tissue-resident markers (*TIMD4*, *LYVE1*, and *FOLR2*), was predominantly enriched in driver regions (Figure 4E and 4F; Figure S12A and S12B). This suggests a critical role for tissue-resident macrophages in maintaining cardiomyocyte homeostasis, specifically within driver regions.^14^ In fact, functional enrichment analysis of PsAF samples showed that driver regions were associated with pathways related to peroxisome activity and endocytosis, indicative of the homeostatic and metabolic functions characteristic of tissue-resident macrophages.^20^ In contrast, macrophages in nondriver regions displayed an inflammatory gene signature, with upregulation of interferon signaling, complement activation, and antigen presentation pathways (Figure 4G). Further proteomic analysis and immunoblots of atrial samples from PsAF animals confirmed the differential regulation of cardiac proteins with adaptive protective and antiapoptotic functions in driver regions compared with nondriver regions (Figure 4H and 4I; Table S5). Interestingly, equivalent anatomic regions in sham-operated controls showed a similar trend in Mac2 cells, although this was not statistically significant (Figure S12C). In controls, the transcriptional program of Mac2 did not show the functional enrichment indicative of homeostatic functions (Figure S12D).^24^ Moreover, unlike PsAF samples, proteomic analysis in sham-operated controls showed no regional differences in anti-apoptotic proteins or those associated with protection from pressure overload, with the exception of BAG3 (Bcl-2 associated athanogene 3; Figure 4H). These results indicate that atrial regions maintaining PsAF may be less prone to cell death. In fact, miR-497-5p levels (a miRNA targeting *BCL2*; reduction in miR-497-5p indicates upregulation of its target gene)^25^ were significantly lower in driver regions than in nondriver regions (Figure 4J). This was consistent with a higher number of TUNEL+ (terminal deoxynucleotidyl transferase–6 mediated deoxyuridine triphosphate nick end labeling) cells in immunohistochemistry images of nondriver regions compared with driver regions in PsAF samples (Figure S12E).

ScRNA-seq analysis was also performed in atrial samples from 1 pig with lone PsAF that underwent 2 mapping procedures (details are shown in Figure S3). A de novo driver in the LAA exhibited a fibroblast and myeloid cellular composition and transcriptional programs that were concordant with the driver locations of the rest of the lone PsAF animals (Figure S13).

Regional differences in fibroblast and macrophage populations are expected to contribute to heterogeneities in paracrine factors released by nonmyocytes, potentially modulating the function of driver regions. Immunoassays revealed significantly higher levels of IL-6 within driver regions compared with nondriver regions (0.28 ng/mL [0.04–0.35] versus 0.06 ng/mL [0.03–0.11], respectively; *P*=0.037). Notably, IL-6 was identified as the most upregulated IL in the FB3-PTX3 cluster, linking cellular transcriptional profiles to regional cytokine secretion. Conversely, the exact comparisons found no differences in IL-1β and IL-18 concentrations (Figure S14A). Furthermore, optical mapping of voltage membrane fluorescence in monolayers of human-induced pluripotent stem cell-derived cardiomyocytes showed that application of 200 picomolar (pM) IL-6 (similar to concentrations in driver regions of AF animals) decreased conduction velocity and favored reentry formation compared with control monolayers exposed to IL-6 solvent only (Figure S14B through S14G). Complementary single-nuclei RNA sequencing analysis further suggested increased cardiomyocyte–nonmyocyte crosstalk in driver regions, with cardiomyocyte-enriched clusters acting as major signal receivers and sources in driver regions (Figure S15).

### Persistent AF in Animals With Infarct-Related Substrate Shows Similar Phenotypic and Cellular Changes to Lone Persistent AF

**Figure 5.**
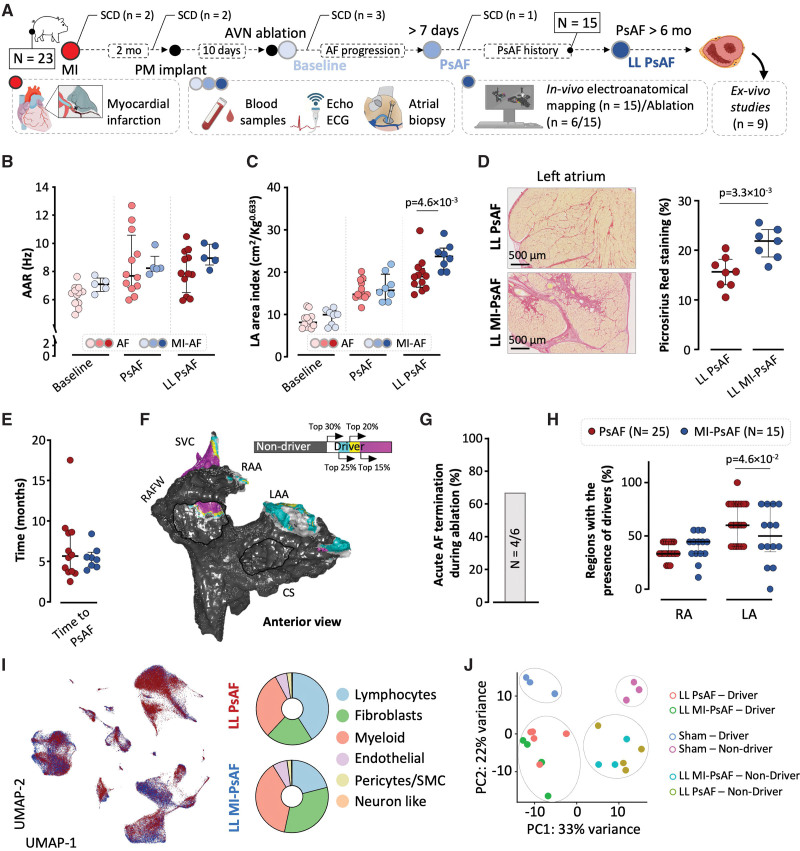
**Phenotypic features of persistent atrial fibrillation (PsAF) in a porcine model with underlying infarct-related substrate. A**, Schematic outline of the experimental protocol in animals with atrial fibrillation (AF) and established myocardial infarction (MI). **B**, Atrial activation rates (AAR) progressively increased in pigs with lone AF and those with AF and underlying infarct-related substrate (MI-AF) from paroxysmal (ie, baseline) to long-lasting (LL) PsAF (lone AF n=12/ MI-AF n=5). Multiple Mann-Whitney *U* tests were used with the Holm-Šídák correction for multiple testing. **C**, Left atrial (LA) area index during AF progression and comparisons in both models (lone AF n=12/ MI-AF n=8). A two-way ANOVA was used, followed by Šídák test for multiple comparisons. **D**, **Left**, representative micrographs of picrosirius red-stained sections of the left atrium from animals with long-lasting lone PsAF (LL PsAF) and long-lasting PsAF with underlying infarct-related substrate (LL MI-PsAF). **Right**, quantification and comparison of interstitial atrial fibrosis between LL PsAF (n=7) and LL MI-PsAF (n=8) animals. **E**, Time to reach PsAF in pigs with MI-AF compared with lone AF animals. In **D** and **E**, the Mann-Whitney *U* test was used to assess statistical significance. **F**, Sample in vivo driver map from a pig with LL MI-PsAF. Driver regions are color-coded according to their hierarchy within the top 30% of median instantaneous frequency modulation values. Nondriver regions are shown in dark gray. **G**, Percentage of successful AF termination after catheter-based ablation of driver regions in 6 pigs with LL MI-PsAF. **H**, Percentage of regions in the right and left atria (RA and LA, respectively) containing driver regions in pigs with LL lone PsAF and pigs with LL MI-PsAF. A two-way ANOVA was used, followed by Šídák test for multiple comparisons. **I**, Left, unsupervised clustering of cell populations in atrial specimens of animals with LL lone PsAF (n=4, as presented in Figure 2) and LL MI-PsAF (n=3). Atrial specimens included samples from both atria: the right atrial free wall, the coronary sinus (CS) with its adjacent posterior left atrial wall, and the superior vena cava (2-3 independent regions were included per animal, making a total of 9 and 7 samples for LL PsAF and LL MI-PsAF, respectively). Data are displayed as uniform manifold approximation and projection (UMAP) plots, where each dot represents a single cell (red for LL lone PsAF and blue for LL MI-PsAF). **Right**, examination of cell proportions per major populations in each model. **J**, Principal component analysis (PCA) plot of pseudo-bulk single-cell RNA sequencing data, colored by model (LL lone PsAF, LL MI-PsAF, and sham-operated controls) and region (driver and nondriver). Regional and model effects were assessed using 2 different driver regions and additional nondriver regions from AF models, and equivalent samples from sham-operated controls (Figure S11). Figure **A** was created with BioRender. LAA indicates left atrial appendage; PM, pacemaker; RAA, right atrial appendage; RAFW, right atrial free wall; SCD, sudden cardiac death.

Ischemia-reperfusion yielded pronounced atrial and ventricular scarring before AF induction in MI-PsAF animals (Figure S16A). Optical mapping experiments in isolated Langendorff-perfused hearts revealed significantly slower conduction velocities in the left atrium of animals with infarct-related substrate compared with healthy controls (Figure S16B and S16C). Conversely, no differences were documented in the action potential duration of the left atrium in infarcted animals compared with healthy controls (Figure S16D). Despite these intrinsic baseline differences, in vivo characterization of AF progression in animals with MI-PsAF (Figure 5A) did not show significant differences in atrial activation rates or coronary sinus plasma concentrations of inflammatory biomarkers during the follow-up, compared with lone PsAF animals (Figure 5B; Figure S17). However, at the time of long-lasting PsAF, animals with MI-PsAF showed significantly larger left atrial areas and a higher degree of atrial fibrosis compared with lone PsAF animals (Figure 5C and 5D). Interestingly, the time to reach PsAF was not statistically different between MI-PsAF and lone PsAF animals (Figure 5E).

All surviving animals with MI-PsAF (n=15) underwent in vivo high-density electroanatomical mapping to identify AF driver regions after 8.0 months (4.9–9.4) in self-sustained PsAF (Figure 5F). In a subset of 6 pigs, catheter ablation of driver regions terminated AF in 4 cases (Figure 5G; Figure S18). This lower termination rate in MI-PsAF pigs compared with lone PsAF animals (Figure 1H) suggests a more complex atrial substrate (Figure 5D) that might not be amenable to catheter-based ablation. The percentage of regions within the left atrium containing drivers decreased from 64% in lone PsAF animals to 50% in those with MI-PsAF (Figure 5H). The latter suggests that some infarcted regions cannot sustain the wave propagation patterns of PsAF drivers.

Atrial samples (n=16) from driver and nondriver regions of animals with MI-PsAF (n=3) and animals with lone PsAF (n=4) were used for scRNA-seq analysis (Table S6). Analysis of 44 447 cells from MI-PsAF animals and 43 706 from lone PsAF animals demonstrated a greater proportion of fibroblasts in MI-PsAF atrial samples (Figure 5I). Principal component analysis showed that atrial samples from lone PsAF and MI-PsAF animals clustered together based on their classification as driver and nondriver regions (Figure 5J), indicating a prevalent role for the functional relevance of the selected regions rather than the comorbidity background of the AF model itself. Comparative atrial samples from sham-operated controls also showed overt clustering differences with PsAF models (Figure 5J). Examination of individual scRNA-seq experiments (separated by anatomic area, model, and biological replicate) demonstrated consistent cluster changes in specific subpopulations of fibroblast and myeloid cells in atrial samples from lone PsAF and MI-PsAF animals (Figures S8 through S11). Driver regions from lone PsAF and MI-PsAF animals showed consistently higher levels of tissue resident macrophages compared with nondriver tissue (Figure S19), further supporting the role of these cells in regions functionally relevant to PsAF maintenance.

### Driver Ablation in Patients With PsAF Is Associated With Long-Term Effective Rhythm-Control

**Figure 6.**
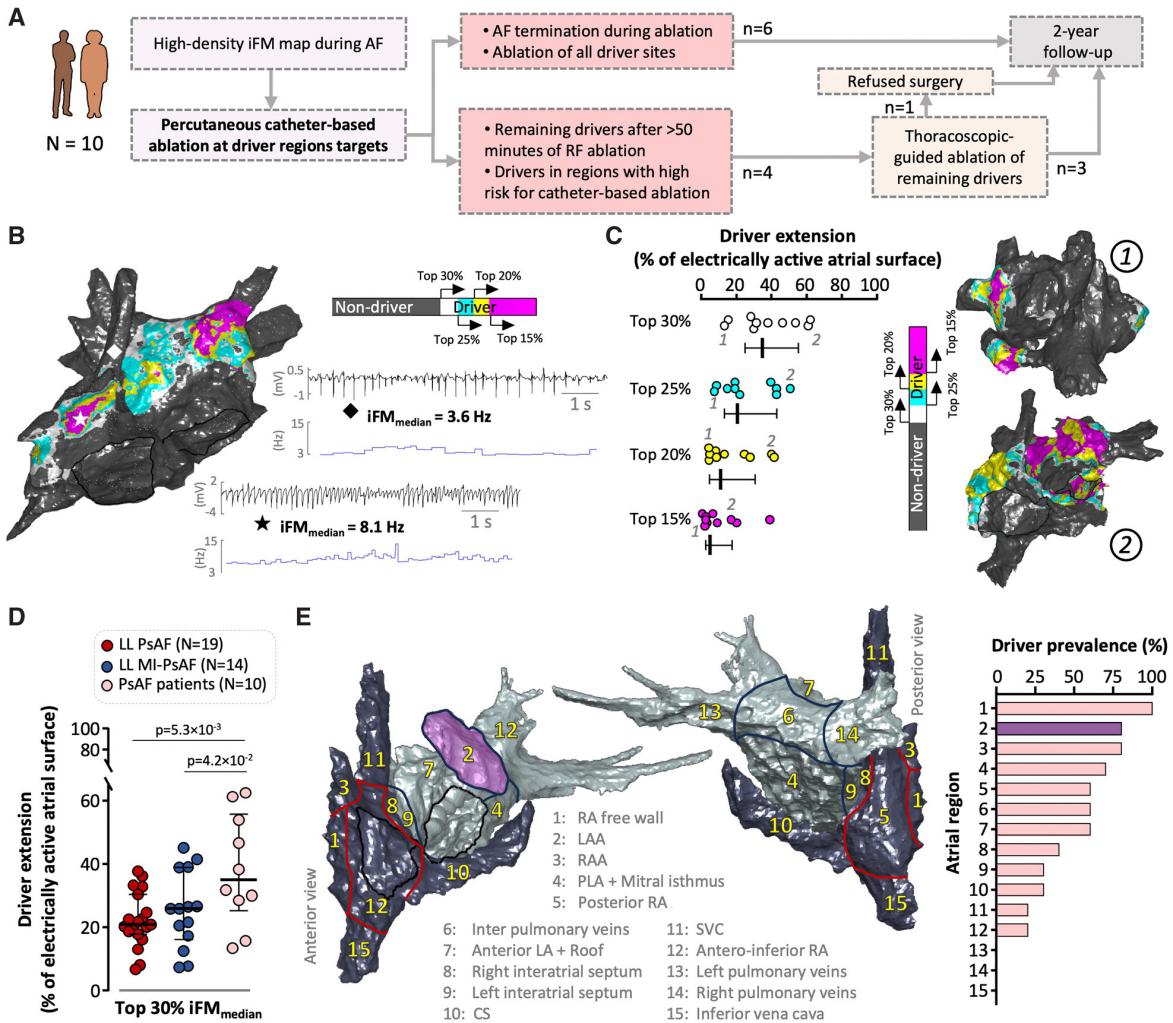
**Driver identification and functional characterization in patients with persistent atrial fibrillation (PsAF) despite previous pulmonary vein isolation. A**, Schematic flow-chart of the pilot study in patients. **B**, Sample driver map derived from median instantaneous frequency modulation (iFM_median_) values. Driver regions are color-coded according to their hierarchy within the top 30% of iFM_median_ values in the atria. Sample unipolar signals (in black) to calculate the iFM (blue tracing) and their median values from 2 driver (asterisk) and nondriver sites (diamond). **C**, **Left**, Quantification of driver extension in all cases of the series (N=10) according to their hierarchy within the top 30% of atrial iFM_median_ values. Right, representative patients (1 and 2) with small (1) and large (2) driver extensions. **D**, Driver extension and comparisons between patients (flesh-colored dots), pigs with long-lasting lone PsAF (LL PsAF; red-colored dots), and pigs with long-lasting PsAF and underlying infarct-related substrate (LL MI-PsAF; blue-colored dots). One-way ANOVA and Tukey posthoc analysis were used to assess statistical significance. **E**, Left, anterior and posterior atrial views including regional anatomic segmentation. **Right**, Driver prevalence (percentage) for each of the anatomic regions in patients. CS indicates coronary sinus; LA/A, left atrium/atrial appendage; PLA, posterior left atrium; RA/A, right atrium/atrial appendage; RF, radiofrequency; and SVC, superior vena cava.

The study included 10 patients with PsAF (female/male, 2/8, age 57.8±9.1 years old) and long-term AF history (6.5±4.9 years) without any significant underlying cardiac disease or relevant systemic comorbities. All patients underwent invasive mapping and ablation after a median of 2 (1–2) prior ablation procedures (Figure 6A). High-density instantaneous frequency modulation maps were used to define the location and extent of atrial driver regions (Figure 6B and 6C). In patients with refractory PsAF, driver regions were significantly larger than in animals with lone PsAF and MI-PsAF (Figure 6D), indicating a complex underlying functional substrate. Notably, acute AF termination during catheter-based ablation was documented in only 1 of the 10 cases. Driver regions were predominantly located in the RA free wall, LAA, and RAA (100%, 80%, and 80% of maps, respectively; Figure 6E). Thoracoscopic-guided ablation to complete the ablation of driver regions was indicated in 4 patients and performed in 3 of them (1 opt-out). Acute AF termination was documented during the procedure upon completing the ablation of all drivers. One patient declined further procedures, and PsAF was documented during the follow-up. Overall, after a 3-month blanking period, 9 of the 10 patients remained free of any documented AF episode during the follow-up visits. The use of antiarrhythmic drugs and oral anticoagulation therapy significantly decreased from baseline until the end of follow-up after 2 years (Table S7).

### Driver Regions in Patients With Persistent AF Show Differential Transcriptomic Signatures

**Figure 7.**
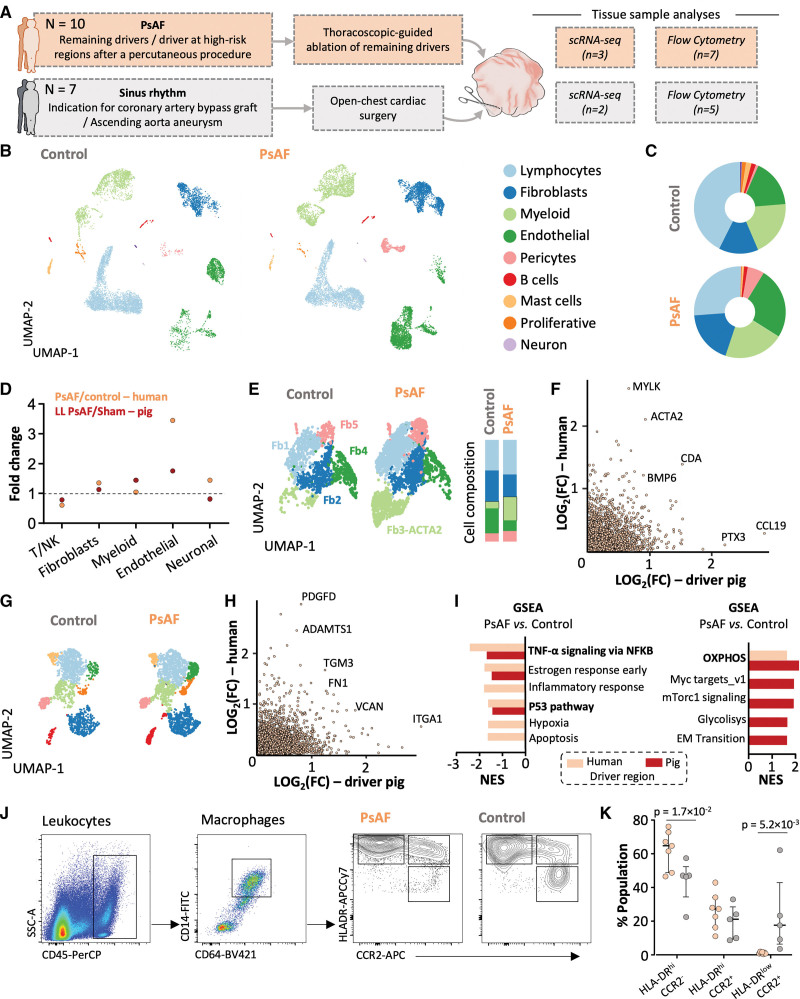
**Single-cell RNA sequencing (scRNA-seq) reveals cellular and transcriptional changes in the human atria with persistent atrial fibrillation (PsAF). A**, Schematic flow-chart of the study in patients with PsAF and controls in sinus rhythm with available tissue samples from the left atrial appendage (LAA). **B**, Unsupervised clustering of major nonmyocyte populations in patients with PsAF and controls. Data are displayed as a uniform manifold approximation and projection (UMAP) plot, where each dot represents a single cell. **C**, Examination of nonmyocyte cell proportions in patients with PsAF and controls. **D**, Changes in nonmyocyte populations in humans with PsAF and pigs with long-lasting lone PsAF. **E**, **Left**, high-resolution unsupervised clustering of human fibroblasts based on differential expression analysis in LAA specimens from patients with PsAF and controls. **Right**, cell composition in each fibroblast cluster. **F**, Genes in fibroblast clusters ranked according to their log_2_-fold change (FC) in human and pig (long-lasting lone PsAF animals) samples from driver regions. **G**, High-resolution unsupervised clustering of human myeloid cells in LAA specimens from patients with PsAF and controls. **H**, Same as in (**F**) for myeloid clusters. **I**, Gene set enrichment analysis (GSEA; hallmarks and Kyoto Encyclopedia of Genes and Genomes [KEGG]) showing upregulated and downregulated pathways in pigs and humans based on genes ranked by log_2_-FC in PsAF vs control samples. NES, Normalized Enrichment Score. **J**, Flow cytometry gating scheme used to identify and characterize cardiac macrophage populations. Live cells were gated (doublets were excluded), and CD45^+^CD64^+^CD14^+^ cells were identified and further classified into 3 distinct populations according to their relative expression of CCR2 and HLA-DR. **K**, Quantification of the myeloid cell populations identified in **J** for patients with PsAF and controls. Two-way ANOVA was used, followed by the Šídák test for multiple comparisons. EM indicates endothelial to mesenchymal; NK, natural killer; OXPHOS, oxidative phosphorylation; and SMC, smooth muscle cells.

Flow cytometry analysis was performed on 7 atrial samples from driver regions located in the LAA of 7 patients with PsAF undergoing thoracoscopic-guided ablation. The same analysis was conducted on LAA samples from 5 controls in sinus rhythm undergoing open-chest surgery. Further samples from 3 patients and 2 controls were used for scRNA-seq (Figure 7A). Baseline characteristics of both populations are shown in Table S8.

Unsupervised clustering identified a total of 20 575 cells and 9 main clusters (Figure 7B; Figure S20; Table S9). Differences in the relative proportions of the main clusters (Figure 7C) followed trends similar to those observed in porcine samples when compared with their respective controls (Figure 7D). Driver regions in humans with PsAF were characterized by the emergence of fibroblast cluster Fb3, which showed the profibrogenic marker ACTA2 (Figure 7E), also documented in the FB4-ACTA2 cluster of pigs with PsAF (Figure 7F). Further comparison of fibroblast clusters across the 2 species revealed that each human cluster significantly matched at least 1 pig cluster (Figure S21). Analysis of myeloid clusters within driver regions of humans with PsAF showed an upregulation of genes such as *ADAMTS1* (a disintegrin and metalloproteinase with thrombospondin motifs 1), *SPP1*, and *FN1* (fibronectin 1) (Figure 7G and 7H). More specific functional enrichment analysis of driver regions in pigs and humans with PsAF revealed common upregulated pathways related to oxidative phosphorylation and downregulated pathways related to inflammation (Figure 7I). Further flow cytometry analysis of the macrophage composition in human LAA samples showed that CD45^+^CD14^+^CD64^+^ cells were divided into 3 different populations based on the expression of HLA-DR and CCR2.^26^ More importantly, and consistent with functional analyses of scRNA-seq data, driver regions in the LAA of humans with PsAF showed lower numbers of CCR2^+^HLA-DR^low^ recruited macrophages, and an elevated presence of CCR2^-^HLA-DR^hi^ resident cardiac macrophages, compared with the same region in controls (Figure 7J and 7K).

## Discussion

This study highlights that atrial changes during PsAF involve differential regional remodeling in nonmyocyte populations, with distinctive and differing gene signatures in areas that drive the overall arrhythmia in the long term. Driver regions are characterized by overt compositional shifts in fibroblasts and myeloid populations, which are highly consistent across pig models and humans with PsAF. Atrial samples from pigs with PsAF showed a phenotypic shift towards ACTA2-fibroblasts and PTX3-fibroblasts compared with sham-operated controls. In humans, ACTA2-fibroblasts were also highly preserved in atrial samples of patients with PsAF. Animal-specific paired comparisons between driver and nondriver regions of pigs with PsAF showed that PTX3-fibroblasts were enriched only in driver regions. In both pigs and humans, myeloid populations within driver regions showed cellular and transcriptional data supporting an increase in tissue-resident macrophages, with a tissue-protective proteome profile in driver regions of pigs with PsAF, which favors cardiomyocyte homeostasis and survival.

Over the past decade, cardiovascular research has been revolutionized by scRNA-seq, providing insights into the cellular diversity, dynamics, and complexity of the myocardium in different pathological conditions.^27^ Changes in specific fibroblast populations and their associated gene expression signatures have been observed in various pathological scenarios, such as dilated or ischemic cardiomyopathy, which exhibit characteristic expression of periostin and fibroblast activation protein.^28,29^ Similarly, in LAA samples, activated myofibroblasts,^30^ fibroblast states with impaired calcitonin signaling, and less-studied fibroblast clusters that regulate key fibrotic responses and express *NR4A1* (nuclear receptor subfamily 4 group A member 1) have been recently described.^31,32^ Here, we show that long-lasting PsAF is associated with 2 distinct fibroblast clusters; one resembling myofibroblasts (FB4-ACTA2) and a second with elevated PTX3 expression (FB3-PTX3). PTX3-expressing fibroblasts were enriched in driver regions of animals with PsAF, suggesting a role for this population in modulating the pathophysiology of PsAF. PTX3 is an innate immunomodulator that has been associated with the activation and proliferation of fibroblasts, as well as the regulation of various infiltrating immune cells. PTX3 has also been associated with immune escape in various cancer subtypes.^33^ In the heart, PTX3 overexpression can attenuate myocardial injury by downregulating apoptosis and autophagy, thereby decreasing the activation of the PI3K/AKT/mTOR pathway.^34^ These data suggest that PTX3 fibroblasts in driver regions may play a relevant role in long-term cardiomyocyte survival and the modulation of myeloid cells’ response.

Few studies have investigated transcriptional changes of myeloid cells during AF.^11,30,35^ Recently, Hulsmans et al^11^ showed a large-scale expansion of recruited macrophages in the atria of patients with AF. These cells expressed *SPP1* among the top upregulated genes promoting fibrosis, enhancing inflammation, and demonstrating a potential causal contribution to AF inducibility in mice. In agreement with this, our results in pigs with long-lasting PsAF (prior to considering the functional relevance of specific atrial regions for AF maintenance) showed an emergence of monocyte populations expressing inflammatory mediators. We also documented upregulation of myeloid cells expressing *SPP1* in atrial tissue samples from patients with PsAF. However, our results go one step further and demonstrate that individual-specific atrial driver regions, relevant for long-term AF maintenance, exhibit distinct functional, cellular, and transcriptional profiles compared with nondriver regions. More specifically, we documented a significant shift towards resident cardiac macrophages in driver regions compared with remote nondriver areas. These findings contrast with the decrease in resident cardiac macrophages often reported in the fibrotic human heart and mouse models of cardiac injury.^11,24,29^ Nevertheless, our data are consistent with the supportive role of resident cardiac macrophages under tissue stress and injury.^24,36^ The latter suggests that resident macrophages contribute to the adaptation and homeostasis of cardiomyocytes within driver regions,^14^ which are subject to sustained high activation rates. This is further supported by proteomics and immunoblotting analyses, indicating mitigated cell damage in driver regions.

Overall, our study provides new insight into the differential adaptive changes of nonmyocyte populations linked to the functional relevance of individual-specific atrial regions in perpetuating AF. Atrial regions associated with AF maintenance are characterized by the emergence of fibroblast and myeloid populations that may contribute to cardiomyocyte homeostasis and survival, which may be a precondition for sustaining high activation rates in the longterm.

## Article Information

### Acknowledgments

The authors thank the members of the animal facility, microscopy, genomics, cytometry, and bioinformatic units at the Centro Nacional de Investigaciones Cardiovasculares.

### Sources of Funding

This work was supported by the European Union Horizon 2020 research and innovation program (grant agreement no. 965286), the Ministry of Science and Innovation (MCIN; PID2023-150456OB-I00 and PGC2018-097019-B-I00) funded by MCIN/AEI (Agencia estatal de Investigación)/10.13039/501100011033, the Instituto de Salud Carlos III (ISCIII; Fondo de Investigación Sanitaria grant PRB3; PT17/0019/0003- ISCIII-SGEFI/FEDER, ProteoRed), the Fundación Interhospitalaria para la Investigación Cardiovascular, Sociedad Española de Cardiología, and la Caixa Banking Foundation (HR17-00247). The Centro Nacional de Investigaciones Cardiovasculares (CNIC) is supported by the ISCIII, MCIN, and the Pro CNIC Foundation, and is a Severo Ochoa Center of Excellence (CEX2020-001041-S funded by MICIN/AEI/10.13039/501100011033). Microscopy experiments were performed at the Microscopy and Dynamic Imaging Unit, CNIC, Infraestructuras Cientificas y Técnicas Singulares (ICTS)-Red Distribuida de Imagen Biomédica (ReDib), cofunded by MCIN/AEI/10.13039/501100011033 and Fondo Europeo de Desarrollo regional, (FEDER) Una manera de hacer Europa (ICTS-2018-04-CNIC-16). A. Simón-Chica was supported by a PhD fellowship from La Caixa (LCF/BQ/DR19/11740029). A. Simon-Chica, J. Greiner, and P. Kohl are members of the German Center of Research Excellence 1425 (Deutsche Forschungsgemeinschaft, DFG): 422681845. M. Couselo-Seijas was supported by MICIU/AEI/10.13039/501100011033 and by FSE+ (grant no. JDC2023-050723-I).

### Disclosures

P. Lee is both an owner and employee of Essel Research and Development Inc. The other authors report no conflicts.

### Supplemental Material

Supplemental Methods

Tables S1–S9

Figures S1–S22

Unedited immunoblots

Major Resources Table

References 37–56

## Supplementary Material
