# Supplementary material for: Cardiac Macrophages and Fibroblasts Modulate Atrial Fibrillation Maintenance
Source: Circ Res. 2026 Feb 10;138(6):e326291. doi: 10.1161/CIRCRESAHA.125.326291 (PMC12986039; doi:10.1161/CIRCRESAHA.125.326291)
Supplement: Supplementary file 1 [file res-138-e326291-s001.pdf]

SUPPLEMENTARY MATERIALS

Cardiac macrophages and fibroblasts modulate atrial fibrillation maintenance

Authors: Ana Simon-Chica[1,*], Jorge G. Quintanilla[1,2,3], Carlos Torroja[4], Marinela Couselo-Seijas[1,2], Haruka Toda[1], Peter Lee[5], Alberto Benguria[6], Concepción Revilla[7], Andrés Redondo-Rodríguez[1,2], José Manuel Alfonso-Almazán[1], Alba García Escolano[1], Manuel Marina-Breysse[1,2], Carlos Galán-Arriola[8], María Linarejos Vera-Pedrosa[9], Giulio La Rosa[1], Ana Dopazo[2,6], Fátima Sánchez-Cabo[2,4], María Jesús García-Torrent[10], Adriana Ortega-Hernández[11], Borja Ibáñez[2,8,12], Estefanía Núñez[2,13], Dulcenombre Gómez-Garre[2,11], Carlos Morillo[1,14], Joachim Greiner[15], Peter Kohl[15], Julián Pérez-Villacastín[2,3,16], Nicasio Pérez-Castellano[2,3,16], José Jalife[2,9], Javier Domínguez[7], Jesús Vázquez[2,13], Manuel Carnero-Alcázar[17], David Filgueiras-Rama[1,2,3,*]

Affiliations:

[1]Novel Arrhythmogenic Mechanisms Program, Centro Nacional de Investigaciones Cardiovasculares (CNIC), Madrid, Spain.

[2]Centro de Investigación Biomédica en Red de Enfermedades Cardiovasculares (CIBERCV), Madrid, Spain.

[3]Cardiovascular Institute, Instituto de Investigación Sanitaria del Hospital Clínico San Carlos (IdISSC), Madrid, Spain.

[4]Bioinformatics Unit, Centro Nacional de Investigaciones Cardiovasculares (CNIC), Madrid, Spain.

[5]Essel Research and Development Inc., Toronto, Canada.

[6]Genomics Unit, Centro Nacional de Investigaciones Cardiovasculares (CNIC), Madrid, Spain.

[7]Departamento de Biotecnología, Centro Nacional Instituto de Investigación y Tecnología Agraria y Alimentaria (INIA-CSIC), Madrid, 28040, Spain.

[8]Myocardial Homeostasis and Cardiac Injury Program, Centro Nacional de Investigaciones Cardiovasculares (CNIC), Madrid, Spain.

[9]Cardiovascular Regeneration Program, Centro Nacional de Investigaciones Cardiovasculares (CNIC), Madrid, Spain.

[10]Universidad Complutense de Madrid, Department of Medicine, Madrid, Spain.

[11]Instituto de Investigación Sanitaria del Hospital Clínico San Carlos (IdISSC), Laboratorio de Microbiota y Biología Vascular. Madrid, Spain.

[12]IIS-University Hospital Fundación Jiménez Díaz, Cardiology department. Madrid, Spain.

[13]Laboratory of Cardiovascular Proteomics, Centro Nacional de Investigaciones Cardiovasculares (CNIC), Madrid, Spain

[14]Department of Cardiac Sciences, Libin Cardiovascular Institute, Cumming School of Medicine, University of Calgary, Calgary, Alberta, Canada.

[15]Institute for Experimental Cardiovascular Medicine, University Heart Center Freiburg – Bad Krozingen, Medical Center – University of Freiburg and Faculty of Medicine, University of Freiburg, Freiburg, Germany.

[16]Fundación Interhospitalaria para la Investigación Cardiovascular (FIC), Madrid, Spain.

[17]Cardiac Surgery Department, Hospital Clínico San Carlos, Madrid, Spain.

This PDF includes:

Expanded Materials and Methods

Suppl. Figs. S1 to S22

Suppl. Tables S1 to S9

Unedited immunoblots

Major Resources Table

Non-standard Abbreviations and acronyms

AF: atrial fibrillation

BSA: bovine serum albumin

CS: coronary sinus

FACS: fluorescence activated cell sorting

GSEA: gene set enrichment analysis

hiPSC: human induced pluripotent stem cells

iFM: instantaneous frequency modulation

IL: interleukin

LAA: left atrial appendage

MACS: magnetic-activated cell sorting

miR: micro-RNA

PBS: phosphate-buffered saline

PsAF: persistent atrial fibrillation

RPMI: Roswell Park Memorial Institute

SUPPLEMENTARY MATERIALS

Expanded materials and methods

Pig model of self-sustained long-lasting lone persistent atrial fibrillation

Yucatan-Large White crossbred pigs were used to generate a model of lone persistent atrial fibrillation (N=27, Figure S1) (PsAF). A dual chamber pacemaker (Accent DR-RF, St. Jude Medical, CA, USA) was surgically implanted in the subcutaneous tissue of the right side of the neck. Atrial and ventricular leads (Tendril STS, Abbott, MN, USA) were inserted through the jugular vein and then positioned in the right atrial appendage and the right ventricle using active fixation electrodes. Ten days after pacemaker implantation and animal recovery, a second procedure was performed to ablate the atrio-ventricular node. The latter aimed to prevent rapid atrio-ventricular node conduction and heart failure shortly during the atrial fibrillation (AF) induction protocol.[37] The ablation procedure was performed using an 8-mm tip ablation catheter (Blazer II XP, Boston Scientific Corporation, Marlborough, USA), which was introduced through an 8-French introducer (ENGAGE Introducer Sheaths, Abbot, MN, USA) in the right femoral vein. The ablation catheter was positioned at the atrio-ventricular node under fluoroscopic guidance and radiofrequency energy was delivered using a temperature-controlled mode with power set to 70W and a cut-off temperature of 70ºC. Complete atrio-ventricular block was documented using intracardiac electrograms and surface ECG recordings. Then, a high-rate atrial pacing protocol was initiated to induce AF. The atrial pacing protocol consisted of 30-second burst pacing at 20 Hz and twice the diastolic threshold, followed by a 6-second sensing period. If the pacemaker detected sinus rhythm restoration during the sensing period, high-rate atrial pacing was automatically reinitiated to induce a new AF episode. Ventricular pacing was programmed using a rate modulation mode between 60-110 beats per minute. Atrial electrograms during AF episodes were recorded and stored using the automatic switch-mode algorithm of the pacemaker device, which enabled us to generate AF burden curves and follow the progression from the initial self-terminating AF episodes to long-lasting self-sustained PsAF episodes. Two animals died during the follow-up due to an intercurrent computed tomography-confirmed pneumonia. The same procedures were performed in sham-operated controls (N=9), but the high-rate atrial pacing protocol was not activated, and the pacemaker was programmed in DDDR mode after atrio-ventricular node ablation.

The procedures were performed after pre-medication (intramuscular ketamine 20 mg/kg and midazolam 0.5 mg/kg) and anesthesia induction (intravenous fentanyl 0.010 mg/kg). After endotracheal intubation, pigs were mechanically ventilated using a volumetric ventilator (tidal volume: 10 mL/kg, respiratory rate: 14 breaths per minute, maximum inspiratory pressure: 25 mm H_2O, intermittent positive end expiratory pressure: 4 mm H_2O). Anesthesia was maintained using 2% sevoflurane. Electrocardiogram, oxygen and carbon dioxide levels were monitored throughout the procedure.

Porcine model of long-lasting persistent AF with underlying infarct-related substrate

Yucatan-Large White crossbred pigs underwent ischemia-reperfusion (3-hour ischemia followed by 1-hour of continuous monitoring during reperfusion) at the proximal left circumflex artery (N=27). The procedure was performed using percutaneous access thorough an 8-French introducer (ENGAGE Introducer Sheaths, Abbot) in the right femoral artery. A bolus of intravenous heparin was administered (300 IU/kg) at the beginning of the procedure. Then, a 5-French AL-2 guide catheter (Cordis, FL, USA) was progressed until the left coronary trunk to assess the coronary anatomy with

two angiographic projections. Then, the catheter tip was positioned in the proximal circumflex artery with the help of a 0.014-inch guidewire. Thereafter, an angioplasty balloon (3.5-mm diameter balloon, Medtronic, MN, USA) was positioned in the proximal circumflex artery, just before the left atrial branch, and inflated to generate ischemia both in the latero-basal part of the left ventricle and the left atrium. Appropriate balloon inflation and artery occlusion were confirmed by coronary angiogram. The artery was occluded for 3 hours before reperfusion. Intravenous amiodarone (300 mg/h) was administered throughout the procedure to decrease the risk of acute ventricular arrhythmia (30% incidence). Refractory ventricular fibrillation was documented in 2 animals which died during the ischemia-reperfusion procedure. Further 2 pigs died during the 2-month subacute period after the infarction and before the initiation of the AF protocol. The ischemia-reperfusion procedure was performed using the same pre-medication and anesthesia protocol described for pacemaker implantation and atrio-ventricular node ablation. Eight weeks after ischemia-reperfusion in the circumflex artery a group of surviving animals (n=19) underwent the same protocol described above for induction of PsAF. All animals in this group with AF and infarct-related substrate (MI-PsAF) also underwent *in-vivo* high-density electroanatomical mapping at the end of the follow-up. Explanted hearts of a subgroup of the surviving animals with infarction (n=4) was used to study the electrophysiological effects of the infarct-related substrate in the left atrium, which excluded the effect of the additional atrial remodeling associated with PsAF. Control animals (N=4) with healthy atria were used for comparisons (Figure S1). These group of eight hearts were Langendorff-perfused to optically map transmembrane voltage changes on the epicardial surface of the left atrium (see details below in the specific *ex vivo* optical mapping section).

Follow-up of animals with atrial fibrillation and sham-operated controls

All animals (with lone PsAF and MI-PsAF) underwent sequential follow-up visits to monitor AF progression and remodeling. During the first four months, the follow-up visits were scheduled at 3-week intervals, and extended to 6-week intervals thereafter. Each follow-up visit included: *(i)* assessment of the voltage threshold for myocardial capture with the pacemaker leads, and adjustment to twice the threshold if needed, *(ii)* assessment of left atrial size using transthoracic echocardiographic imaging, *(iii)* exportation of stored atrial electrograms during AF episodes for offline signal processing and electrical remodeling characterization.[38] In the MI-PsAF group, four more animals died suddenly during the follow-up and the pacemaker device registered a ventricular fibrillation event as the cause of dead.

Exported atrial electrograms of stored AF episodes were processed using a custom-made Java-based software to calculate local right atrial activation rates using spectral analysis and Fast Fourier Transform, as reported elsewhere.[38]

Transthoracic echocardiography studies were performed with an iE33 ultrasound system (Philips Healthcare, USA) equipped with a X5-1 probe. Left atrial cross-sectional area was measured on a parasternal long-axis view, at end-systole on the frame preceding the opening of the mitral valve. Left atrial cross-sectional area was obtained outlining the inner border of the atrium, excluding the area under the mitral valve annulus and the inlet of the pulmonary veins. Atrial dimensions in pigs were indexed to animal weight as follows:

$$\text{Left atrial area index} = \frac{area_{left\ atrium}}{\frac{970}{1000} * weight^{0.633}} \left(\frac{cm^2}{Kg^{0.633}}\right)$$

In the formula, animal weight limit for indexation was set at 80 kg, since over such weight Yucatan-Large White crossbred pigs mainly gain fat tissue rather than actual grow in animal size.

Atrial biopsies and plasma samples collection

Blood samples from the coronary sinus (CS) and a peripheral vein were obtained at baseline (during the atrio-ventricular node ablation procedure), after 6 weeks of 100% AF burden, and at the end of the follow-up in long-lasting PsAF. Biopsies from the right atrial appendage were taken at baseline and at the end of the follow-up at the time of long-lasting PsAF (i.e., >6 months in self-sustained PsAF). Atrial tissue samples were used for histopathology and real time quantitative polymerase chain reaction.

Blood samples from the CS were taken using an 82-cm length steerable sheath (8.5-French Agilis NxT Steerable Introducer, St. Jude Medical, MN, USA) which was positioned into the CS under fluoroscopy guidance. Blood samples were collected in VACUETTE 2 mL Lithium Heparin tubes (Greiner Bio-One 454089, North Carolina, USA) and centrifuged at 400 × g for 30 minutes at 4ºC. Then, samples were aliquoted and stored at -80ºC for further analysis of biomarkers. The same steerable sheath was used to take biopsies from the right atrial appendage after positioning the sheath in the appendage. The biopsies were taken at baseline and at the end of the follow-up at the time of long-lasting persistent AF (PsAF) (>6 months in self-sustained PsAF). Proper positioning of the sheath was confirmed with an ablation catheter (Blazer II XP, Boston Scientific Corporation) that showed electrograms consistent with the right atrial appendage. Then, 5.5-French biopsy forceps (Cordis) was introduced through the long sheath to take endomyocardial biopsies at the preselected atrial location. Atrial tissue biopsies were used for histopathology and real time quantitative polymerase chain reaction

High-density in-vivo electroanatomical mapping in pigs

All animals from the AF protocol (25 with lone PsAF and 15 with MI-PsAF) underwent *in-vivo* high-density electroanatomical mapping at the end of the follow-up. The procedure was performed under general anesthesia with the same protocol described for pacemaker implantation. Percutaneous right venous femoral access was used to reach the right atrial chamber. Left atrial access was performed after atrial transeptal puncture using an 82-cm long steerable introducer (Agilis NxT Steerable Introducer, St. Jude Medical). Intravenous heparin (140 IU/kg) was administered to prevent thrombus formation after transeptal puncture. A continuous flow (200 mL/h) of heparinized saline (1000 IU/500 mL) was administered through the long sheath lumen throughout the procedure to prevent intraluminal thrombi formation inside the long sheath. A 5-French introducer in the right femoral artery was used to monitor blood pressure throughout the procedure. The Ensite Precision cardiac mapping system (Abbott, NJ, USA) was used for 3D electroanatomical guidance. A decapolar catheter (Dynamic XT, Boston Scientific, MA, USA) was positioned into the superior vena cava as an internal reference for the mapping system. Intracardiac signals and blood pressure were continuously recorded with the LabSystem-Pro electrophysiology recording system (Boston Scientific, MA, USA). Atrial electroanatomical mapping was performed with a PentaRay (20 poles) catheter (Biosense Webster, CA, USA). The multipolar catheter was introduced through the long sheath and systematically positioned at different locations of the endocardium to map electrical activity and generate the anatomical mesh of both atria and the CS. Eight-second-long unipolar signals were acquired at each mapping location. After mapping, electroanatomical data were exported intraprocedure for further signal processing and identification of driver regions. Driver regions were defined as those activating

faster than their surroundings and within the top 30% median instantaneous frequency modulation (iFM) values of the atria, as reported elsewhere (Figure 1G, Figure S3 and Figure S4). A subset of animals underwent catheter ablation of driver regions (8 with lone PsAF and 6 with MI-PsAF) using an open-irrigated tip catheter (FlexAbility, Abbott, MN, USA) which was positioned at target driver sites for radiofrequency energy delivery. Ablation was performed using 30 W at the posterior left atrium and 35W at other atrial regions, with a temperature-controlled mode at maximally 40°C, saline irrigation at 17 mL/min for 30W applications or 30 mL/min for 35W applications. Radiofrequency applications aimed at ablating all driver regions by creating coin-like sets of lesions, unless the target region or some of the target regions were located at high-risk areas for catheter-based ablation (left and right atrial appendages, cardiac conduction system). Ablation was stopped after conversion to sinus rhythm, complete elimination of all leading-driver locations or after 50 minutes of radiofrequency energy delivery even if not all driver sites have been targeted. The latter situation was considered to represent an extensive driver substrate not suitable for complete elimination with catheter-based ablation. In case sinus rhythm conversion during the ablation protocol, high-rate atrial pacing at 20 Hz was attempted at least 3 times to assess AF maintenance. AF episodes were considered non-sustained, if lasted <10 minutes. If reinduced AF episodes lasted more than 10 minutes, the ablation protocol was resumed until targeting all driver locations, unless ablation time exceeded the maximum of 50 minutes of radiofrequency energy delivery. A subset of 4 animals with lone PsAF underwent catheter ablation of non-driver regions to compare acute termination rates with the group of animals with lone PsAF undergoing ablation of driver sites. In animals undergoing ablation at non-driver sites, radiofrequency energy was applied until a maximum of 50 minutes of radiofrequency energy delivery (Figure S4).

Euthanasia and atrial tissue collection in pigs

At the end of the follow-up all animals underwent open-chest cardiac surgery for heart excision and euthanasia. Anesthesia was maintained with 3% sevoflurane concentrations (a higher concentration than during the rest of invasive procedures). An intravenous bolus of fentanyl (0.005 mg/kg) was administered for analgesic control during sternotomy. An intravenous bolus of heparin (300 IU/kg) was also administered to prevent clot formation in the coronary arteries in the explanted heart. Then, euthanasia was performed by exsanguination after heart excision. In animals with AF, atrial tissue samples from driver and non-driver regions were flushed with cold (4°C) cardioplegic solution (Custodiol, Dr. Franz Köhler Chemie GmbH, Bensheim, Germany). Samples from equivalent regions in sham-operated controls were also processed as in the group of AF animals. Investigators were blinded during the analysis of subsequent experiments, including histopathology and immunohistochemistry, real time quantitative polymerase chain reaction, micro-RNA analysis, quantitative high-throughput proteomics and immunoblotting studies. Blinding was not possible for *in vivo* electroanatomical mapping and ablation.

Clinical studies in patients

An initial prospective and pilot study was conducted in patients (N=10) with symptomatic PsAF refractory to antiarrhythmic drugs, and previous history of at least one ablation procedure including pulmonary vein isolation (Table S7). The study included consecutive patients from January 2018 to June 2021 who had been evaluated at the arrhythmia unit of the Hospital Clínico San Carlos (Madrid, Spain) and were considered candidates for a redo ablation procedure according to the corresponding expert consensus statement on catheter and surgical ablation of AF.[17] The study included patients ≤75

years old, with left atrial diameter <55 mm in the parasternal long-axis view, left ventricular ejection fraction ≥50% and without other significant systemic and cardiac comorbidities. More specifically, patients with moderate or severe valvular heart disease, previous history of cardiac surgery, hypertrophic cardiomyopathy, coronary artery disease (with or without history of myocardial infarction), non-ischemic dilated cardiomyopathy, infiltrative cardiac disease (e.g., cardiac sarcoidosis) or other causes of reduced ventricular ejection fraction were excluded from the study. Exclusion criteria also included severe systemic conditions that may have a causal relationship with AF episodes (e.g., hyperthyroidism, cancer, systemic inflammatory disease) or patients with contraindications for AF ablation (e.g., intracavitary thrombi, pregnancy). The invasive mapping procedure aimed to identify specific atrial regions potentially associated with AF maintenance (i.e., driver regions) based on iFM maps. Driver regions were defined using the same criterium as in the experimental pig model of long-lasting PsAF. Data acquisition was performed during AF using the Ensite Precision System and a 16-pole catheter (HD Grid, Abbott), which was sequentially positioned at different right and left atrial locations and the CS. A 71-cm long steerable introducer (Agilis NxT Steerable Introducer) was used to improve the multipolar catheter positioning and stability at all mapping locations. The long steerable sheath was also used to optimize the transeptal puncture position for left atrial access after completing the mapping in the right atrium and CS. Transeptal puncture was monitored with intracardiac echocardiography guidance. A decapolar catheter positioned in the left renal vein was used as internal positional reference for the mapping system and unipolar reference for the 16-pole HD Grid catheter. Intravenous heparin (140 IU/kg) was administered to maintain an activated clotting time >300 seconds throughout the procedure. After completing the mapping of both atria, data were exported and processed intra-procedure to identify driver region targets for ablation. An open-irrigated tip catheter (FlexAbility, Abbott) was used to apply radiofrequency energy at leading-driver locations (30-35W, saline irrigation: 17-30 mL/min). Catheter-based ablation was stopped after conversion to sinus rhythm, complete elimination of all leading-driver locations or, in cases with extensive driver substrate (>15% of atrial surface), after 50 min of radiofrequency delivery despite not completing the ablation of all driver sites. Ablation was applied to create a coin-like set of lesions aiming to cover the entire driver region. Left and right atrial appendages were considered high-risk areas for catheter-based ablation. In cases with incomplete ablation of driver regions, the patients were invited to undergo a thoracoscopic-guided procedure. The thoracoscopic-guided procedure was performed to ablate the remaining drivers and isolate the left atrial appendage (LAA), both electrically and mechanically, if detected as an AF driver during the mapping procedure. The LAA was isolated with the AtriClip device (AtriCure, Inc, OH, USA). Only driver regions were targeted with ablation, without any additional empirical lines. All patients underwent clinical follow-up to assess recurrences at 1, 3, 6, 12, 18 and 24 months after the procedure. Symptomatic AF recurrences were documented at any time during the follow-up. The protocol was approved by the ethics committee of the Hospital Clínico San Carlos (ref#17/334-R_P).

A second prospective study was conducted in patients ≤75 years old with symptomatic PsAF, refractory to at least one antiarrhythmic drug and previous history of at least two ablation procedures including pulmonary vein isolation (Table S8). The protocol (NCT05169320) was approved by the ethics committee of the Hospital Clínico San Carlos (ref#21/140-E). In this series, we excluded patients with 3D left atrial volume index >73 mL/m^2, any severe underlying cardiac disease with left ventricular ejection fraction ≤35%, severe non-corrected valvular disease or coronary artery disease, hypertrophic cardiomyopathy, and other inherited arrhythmia syndromes. Like in the pilot series, patients with severe systemic conditions that may have a causal relationship with AF episodes or patients with contraindications for AF ablation were also excluded. All patients underwent the

interventional mapping procedure reported in the pilot series to identify driver regions. After the percutaneous procedure, we selected the subgroup of patients within the trial that required a thoracoscopic-guided procedure to complete the ablation of all driver regions, provided that the LAA was one of the driver locations (N=10). LAA samples were taken after mechanical and electrical isolation of the appendage. The tissue was used for further flow-cytometry and single-cell RNA sequencing (scRNA-seq) analyses.

Finally, a third clinical prospective series was conducted in patients without previous history of AF undergoing open-chest cardiac surgery due to coronary artery disease or ascending aortic aneurysm (Table S8). Patients were recruited from April 2022 to May 2025 at the cardiac surgery department of the Hospital Clínico San Carlos. Only patients ≤75 years old, with left ventricular ejection fraction ≥50%, and without signs of significant valvular heart disease (moderate or severe dysfunction) or cardiac structural damage on cardiac imaging were included in the series (N=7). During the surgical procedure LAA samples were taken for further flow cytometry and scRNA-seq analyses aiming to understand the underlying non-myocyte substrate in patients in sinus rhythm without overt structural heart damage. After hospital discharge, all patients underwent regular clinical visits (1, 3 and 6 months) at the cardiac surgery outpatient clinic and a specific protocol visit at 1 year of follow-up to assess cardiac rhythm and uncover any possible incident AF during the follow-up. In fact, none of patients developed any detected AF episode neither during the postoperative hospitalization period nor the follow-up. The study was approved by the ethics committee of the Hospital Clínico Universitario San Carlos (ref#22/058-E). All patients gave written informed consent before atrial tissue sample collection. Tissue data results from this series were used as comparative controls against driver region samples from the LAA of patients with PsAF.

Together, these three clinical datasets provide a translational framework that links clinical outcomes, functional mechanistic insights, and cellular and molecular profiling of non-cardiomyocyte populations in humans.

Cell isolation of non-cardiomyocytes

Porcine hearts were harvested and dissected, and cells were isolated by enzymatic digestion. Tissue was minced to ~1 mm^3 cubes and digested in 3 mL of Dulbecco's Modified Eagle Medium (DMEM) containing 450 U/mL collagenase I, 60 U/mL DNase I, and 60 U/mL hyaluronidase (all Sigma-Aldrich, Munich, Germany) for 60 min at 37ºC. The resulting cell suspension was filtered through a 70-µm nylon mesh (Corning, USA) to remove undigested tissue fragments, and pelleted by centrifugation (400 × g for 5 min at 4ºC). Cells were re-suspended in fluorescence activated cell sorting (FACS) buffer (phosphate-buffered saline [PBS] with 1% foetal calf serum + 0.1% bovine serum albumin [BSA]) before antibody labeling and FACS sorting. For human samples, atrial tissue was dissected and placed in cold cardioplegia (Custodiol) to stop beating during transportation from the Hospital Clínico San Carlos to the research facilities at the Centro Nacional de Investigaciones Cardiovasculares (CNIC). Next, cell isolation from human tissue samples followed the same protocol as for atrial tissue samples from animals.

Multicolor flow cytometry

We specifically conjugated and labelled anti-porcine antibodies with different fluorescence dyes using the DyLight protein labelling kit according to manufacturer's instructions (Thermo Fisher Scientific, MA, USA). Human cardiac macrophages were isolated using the enzymatic digestion protocol

described for porcine tissue. Antibodies directed against membrane molecules of porcine CD172a (BA1C11, IgG1), CD45 (2A5, IgG1), SLA-II (2E9/13, IgG2b), CD163 (2A10, IgG1) and CD169 (1F1, IgG2a) were produced and provided by CR and JD (Instituto Nacional de Investigación y Tecnología Agraria y Alimentaria, INIA, Madrid, Spain) under an official research agreement between CNIC (DFR) and INIA investigators (www.boe.es/diario_boe/txt.php?id=BOE-A-2021-5387).

Human cardiac macrophages were isolated using the enzymatic digestion protocol described for porcine tissue. Human myeloid cells were identified using CD45-PerCP (2D1, IgG1), CD64-BV421 (10.1, IgG1), CD14-Fitc (M5E2, IgG2a). Cardiac macrophages were further separated into different subpopulations based on the relative expression level of HLADR-APCCy7 (L243, IgG2a) and CCR2-APC (K036C2, IgG2a). Detailed antibody information and conjugation are provided in the Major Resource Table.

Single-cell preparation and CD45$^+$ enrichment

After cell sorting, cells were resuspended in PBS supplemented with 0.04% BSA. Cell viability was checked using an automated Countess III cell counter with software v1.0.296.782 (Thermo Fisher Scientific) in all single cell suspensions composed of approximately 20% endothelial cells, 70% leukocytes and 10% other non-myocytes. For porcine samples, scRNA-seq analysis was performed in a total of 10 pigs: sham-operated controls (n=3), long-lasting lone PsAF (n=4), and MI-PsAF (n=3). For each animal, 2-3 different atrial regions were dissected: one or two driver regions with the highest median iFM values, and one non-driver region (lowest median iFM values). In total, 22 datasets were generated, ensuring at least 3 biological replicates per condition and atrial region (Table S6). For human samples, scRNA-seq analysis was performed in LAA samples from 2 controls in sinus rhythm and 3 patients with PsAF (Table S9).

Chromium 10X library preparation

Single cells were encapsulated into emulsion droplets utilizing the Chromium Controller (10x Genomics). The analysis followed the specific protocols outlined in the 10X Genomics Chromium Single Cell 3′ Reagent kits v3 or v3.1 user guide. Single-cell suspensions were loaded into a Chromium Next GEM Chip G (10x Genomics) to capture a maximum of 10,000 single cells per port. Then, scRNA-seq libraries were prepared using the Chromium Next GEM Single-Cell 3′ Kit v3.1 (10x Genomics). Individual libraries were diluted to a concentration of 10 nM and pooled for sequencing. Library pool was sequenced using one P3 flow cell (100 cycles) on a NextSeq 2000 (Illumina). FastQ files for each sample were obtained using cellranger mkfastq pipeline (10x Genomics).

Analysis of single-cell RNA sequencing data

The CellRanger v6.1.1 pipeline was used to generate a digital gene expression matrix starting from raw data. For alignment and quantification of gene expression, the reference transcriptome was built using genome pig Sus scrofa 11.1 with ensembl gene build version 109 (feb2023.archive.ensembl.org). The raw digital gene expression matrix (unique molecular identifiers [UMI] counts per gene per cell) was imported in R (https://www.R-project.org/, version 4.1.1) and processed using scater[39] for quality control and filtering purposes. We obtained data from 167,070 cells and 31,781 genes that passed quality control steps implemented in *Cell Ranger*. Then, we filtered out cells meeting any of the following criteria: <400 unique genes expressed, <1,000 or >40,000 unique molecular identifiers, >25% of reads mapping to mitochondria, <0.2% cell counts fraction in the sample, cells with high levels of reads in just a few genes, >65% of reads in the top 50 genes and >0.1% of reads in hemoglobin

subunits (Figure S22A). Doublets were detected and filtered out using *scDblFinder* with default settings by sample.[40] Filtered cells were then log-normalized and a total of 1,000 most variable genes (*vst* method) were selected for subsequent dimensionality reduction and clustering steps. After Principal Component Analysis (PCA) dimensionality reduction, a nearest-neighbor graph followed by Louvain clustering was applied using the first 20 components of the PCA. Uniform Manifold Approximation and Projection Plot (UMAP) dimensionality reduction on the same 20 components was applied for visualization. For human scRNA-seq analysis, the same methodology was applied with the following modifications: *i)* gene expression was quantified using Human reference GRCh38 with ensembl gene build version 84*, ii)* quality-control filtering criteria: <400 unique genes expressed, <1,000 or >35,000 unique molecular identifiers, >15% of reads mapping to mitochondria, <0.4% cell counts fraction in the sample and >65% of reads in the top 50 genes (Figure S22B). For clustering, both samples were integrated using robust PCA methods as implemented in Seurat package using 2000 variable genes and 30 PCA. Markers for each cluster were obtained using Wilcox test as implemented in Seurat package and differences between conditions were obtained for each cluster using MAST model. Functional analyses on gene lists were obtained using EnrichR databases.[41] Cell types were defined based on an initial cell classification using SingleR[42] followed by manual characterization of the marker genes for each cluster. Some cell types or groups of cells were reclustered independently to capture better their diversity using the same approach as the global clustering described above. Pseudo-bulk RNA sequencing analysis was conducted using the DESeq2 package in RStudio and gene set enrichment analysis (GSEA) was conducted for functional enrichment using the Bioconductor package fgsea.

Pseudo-bulk RNA-sequencing

Pseudo-bulk RNA-sequencing analysis was conducted using the DESeq2 package in RStudio. A gene expression matrix was extracted from the Seurat object using the *GetAssayData* Seurat function to obtain a matrix containing the raw sequencing counts for each gene and cell. Counts were then aggregated per gene for each sample, resulting in a new matrix. The resulting matrix was normalized to obtain a matrix consistent to the output of a traditional bulk sequencing experiment. The DESeq2 function was then utilized to calculate differential gene expression based on negative binomial distribution. Pairwise comparisons were completed using the Benjamini-Hochberg test with an α-value of 0.05.

Gene set enrichment analysis and interactome analysis

GSEA was conducted for functional enrichment using the Bioconductor package fgsea (version 1.10.1). The analysis utilized ranked gene expression markers as input and was executed with the GSEAPreranked tool from GSEA version 4.2.0, employing collections of gene sets sourced from the molecular signatures database MSigDB. Interactome analysis was performed inferring significant cell–cell communication using the R package CellChat (version 2.1.0).[43] The communication probability among interacting cardiomyocytes, fibroblasts and myeloid cells was calculated using the computeCommunProb function with population.size = TRUE, and the default ‘triMean’ method for the calculation of the average gene expression per cell group. Significant alterations in outgoing and incoming signaling between driver and non-driver conditions were determined and visualized using the netVisualcircle function.

Single-nuclei RNA sequencing

Frozen cardiac tissues were homogenized in 2 mL of chilled lysis buffer (292 mM NaCl, 42 mM $MgCl_2$, 20 mM Tris-HCl, pH 7.5, 2 mM $CaCl_2$ supplemented with 0.2 μL^{-1} RNase inhibitor, 0.01% BSA, and 0.3% Tween-20 in nuclease-free water) using a gentleMACS Octo Dissociator (Miltenyi Biotec). Lysate was gently filtered through 40-µm in salt-Tris buffer (146 mM NaCl, 21 mM $MgCl_2$, 10 mM Tris-HCl, pH 7.5, 1 mM $CaCl_2$ supplemented with 0.2 μL^{-1} RNase inhibitor in nuclease-free water). Nuclei were then centrifuged at 500 × g for 5 min at 4 °C, followed by resuspension in 1 mL nuclei wash buffer (2% BSA and 0.2 U μL^{-1} RNase inhibitor in PBS) and filtered through a 20-µm strainer. Centrifugation was repeated following the same parameters. Supernatant was then removed and nuclei were resuspended in 300 µL nuclei wash buffer and transferred to a 5-mL tube for flow sorting. Then, 1 µL DRAQ5 (5 mM solution; Thermo Fisher, cat. no. 62251) was added and incubated for 5 min before sorting. $DRAQ5^{+}$ nuclei were sorted on a FACS Aria II cell sorter (BD biosenses) using a 70-µM nozzle. Recovered nuclei were centrifuged again using the same parameters and were gently resuspended in nuclei wash buffer to a target concentration of 1,000 nuclei μL^{-1}. After sorting, samples were immediately loaded on the 10× Chromium controller (10× Genomics) following the same protocol described for scRNA-seq.

Histopathology and immunohistochemistry analyses

Cross-sections of the pig atrial myocardium were dehydrated, embedded in paraffin, and cut into 5-µm thick sections. Tissue slices were stained with Hematoxylin & Eosin and Picrosirius Red, and digitized using a NanoZoomer S360 Digital slide scanner (Hamamatsu Photonics, Shizuoka, Japan) for analysis. A total of 10 randomly selected 20x insets per slide were analyzed (excluding endocardial, epicardial, and peri-vascular regions). The same procedure was performed to analyze fibrosis in atrial biopsies, although with an augmentation of 40x due to the smaller sample size. Interstitial fibrosis was quantified using ImageJ with a modified version of a color deconvolution-based plugging.[44]

Frozen atrial tissue samples from pigs were sectioned using a cryostat microtome (Leica CM3050 S). Ten-µm-thick cryosections were collected on microscope glass slides and were left at room temperature for 10 minutes to dry. A circle was drawn around cryosections with a hydrophobic barrier pen. Then, cryosections were fixed by adding 2% paraformaldehyde into this circle for 10 minutes. Samples were washed three times with PBS. Slides were dried and incubated with blocking solution (0.05% Triton-X, 5% BSA and 5% goat serum in PBS) for 30 minutes. Blocking solution was removed (without washing). Primary unconjugated antibodies (rabbit-anti-SMA 1:500, polyclonal [Sigma-Aldrich], rabbit-anti-PTX3 1:100, polyclonal [Proteintech] were diluted in antibody diluent solution (0.02% Triton-X, 1% BSA and 2.5% goat serum in PBS). Slides were incubated with primary antibodies at 4 °C overnight in a humidified chamber in the dark. The day after, tissue slices were washed with PBS supplemented with Tween 20 (PBST; 0.1% Tween 20 in PBS) three times for 20 minutes. Then, the samples were incubated with secondary antibodies (Alexa Fluor 488 goat-anti-Rabbit IgG) at room temperature in the dark for 1-2 h. After the incubation time, secondary antibodies were washed off with PBST three times for 10 minutes. For the case of conjugated antibodies, the staining protocol was slightly different: conjugated antibodies were diluted in antibody diluent solution. Slides were incubated for 2 h with conjugated antibodies and washed three times with PBST.

The *in situ* cell death detection kit (Sigma-Aldrich) was used for the detection and quantification of apoptosis according to manufacturer's instructions. Thereafter, samples were incubated with wheat germ agglutinin (1:400, Thermo Fisher Scientific) and 4',6-diamidino-2-phenylindole (DAPI) (1:500, Thermo Fisher Scientific) for 10 minutes. Finally, the samples were mounted in VECTASHIELD

mounting medium (Vector Laboratories). Fluorescence images of labelled tissue slices were acquired using a confocal microscope (Leica SP8X, 405 nm laser, white light laser, glycerol immersion objective) with suitable laser lines. Sensitive hybrid detectors were used to image fluorescence.

Real time quantitative polymerase chain reaction and microRNA analysis

In atrial tissue samples, total RNA was extracted by using TRIzol Reagent (Thermo Fisher Scientific). Reverse transcription was processed from total RNA using the Applied Biosystems High-Capacity cDNA Reverse Transcription kit (Thermo Fisher Scientific). Real time quantitative polymerase chain reaction was performed with SYBR Green (Thermo Fisher Scientific) where cDNA was amplified by using custom DNA primers based on the pig genome (interleukin-6 (*IL-6*): forward *GACCCTGAGGCAAAAGGGAA*, reverse *TGGACGGCATCAATCTCAGG;* tumor necrosis factor α (*TNF-α*): forward *GGCCCAAGGACTCAGATCAT*, reverse *CTGTCCCTCGGCTTTGACAT;* interleukin-1β (*IL-1β*): forward *GCCAGTCTTCATTGTTCAGGTTT*, reverse *ATCTCTTTGGGGCCATCAGC; GAPDH*: forward *CCATCTTCCAGGAGCGAGAT*, reverse *AGAAGGGGCAGAGATGATGA*). GAPDH was used for gene expression normalization, and the 2-ΔΔCt method was used for relative gene expression quantification.

For micro-RNA (miR) quantification, we used the primer sets for the miR (hsa-miR-497-5p), and the hsa-miR-103a-3p as control. The plate further contains the RNA spike-ins for RNA isolation control, cDNA synthesis control and PCR amplification control. Briefly, 4 µL of cDNA template (diluted 1:20) was amplified with the miRCURY SYBR Greem PCR kit using a StepOnePlus™ RT-PCR System (Thermo Fisher Scientific, CA, USA). The amplification curves were analyzed using the StepOne™ software (Thermo Fisher Scientific) for determination of threshold cycles (Ct). A threshold cycle value <35 was considered appropriate and the miR expression was normalized using the hsa-miR-103a-3p. Fold changes were then calculated using the 2^-ΔΔCt method.[45]

Western Blotting

Atrial tissue proteins from pig samples were extracted with Radio-Immunoprecipitation Assay (RIPA) buffer supplemented with complete protease inhibitor cocktail (Roche-Diagnostics) and PhosSTOP phosphatase inhibitor tablet (Roche-Diagnostics), according to the manufacturer's instructions. Total protein concentration was determined using the Pierce bicinchoninic acid protein assay kit (Thermo Fisher Scientific). Ten µg of total protein was resolved by sodium dodecyl-sulfate polyacrylamide gel electrophoresis and transferred to nitrocellulose membranes (Bio-Rad Laboratories, Hercules, California, UE). Total protein was visualized with Pierce™ Reversible Protein Stain Kit (Thermo Fisher Scientific) and used for protein band normalization. Membranes were washed with 0.2% Tween Tris-buffered saline solution and blocked with 5% BSA for 1 h. Primary antibody anti-BCL2 (E-AB-22004, Elabscience, Texas, USA) was incubated (1:1,000 dilution) at 4°C overnight. The next day, membranes were washed and incubated with a corresponding horseradish peroxidase-conjugated anti-mouse secondary antibody. The protein bands were visualized by enhanced chemiluminescence with Immobilon Forte Western horseradish peroxidase substrate (Merck Millipore) in an iBright™ FL1500 Imaging System (Thermo Fisher Scientific). Protein band analysis was performed with iBright™ Analysis Software (Thermo Fisher Scientific).

Quantitative high-throughput proteomics

Driver and non-driver atrial tissue protein extracts from pig samples, or equivalent anatomical locations from sham-operated controls, were obtained by tissue homogenization. Extracted proteins were then subjected to filter-aided digestion (Nanosep Centrifugal Devices with Omega Membrane-10K, PALL) and the resulting peptides were tandem mass tags-labeled and fractionated using the high pH reversed-phase peptide fractionation kit (Thermo Fisher Scientific), according to the manufacturer's instructions. Each fraction of labeled peptides was subjected to liquid Chromatography Mass Spectrometry analysis, and peptide and protein identification were performed as described elsewhere.[4] Protein quantification was performed according to the weighted spectrum, peptide, and protein (WSPP) model[46] and the Generic Integration Algorithm (GIA),[47,48] with the iSanXoT software package.[48,49] Quantitative protein values were expressed using the standardized variable Zq (i.e., normalized log_2-ratios expressed in units of standard deviation according to the estimated variances). For functional analysis, proteins were annotated using DAVID[50] and annotations were subsequently refined manually. The mass spectrometry proteomics have been deposited to the ProteomeXchange Consortium via de PRIDE[18] partner repository with the dataset identifier PXD053398 by logging into the PRIDE website with the credentials: username reviewer_pxd065452@ebi.ac.uk and password Sckg4zU2Es4P. Among the proteins that showed statistically significant changes (p-value <0.05), the analysis was focused on the proteins related to apoptosis and cell survival processes.

Human induced pluripotent stem cell-derived cardiomyocyte generation and culture

Human induced pluripotent stem cells (hiPSC) were obtained following the methodology described elsewhere.[51-53] Colonies were cultured on 6-well plates coated with Matrigel (100 µg/mL, Sigma-Aldrich) for 7 days in StemMACs iPSC Brew XF medium (Miltenyi Biotec), with daily medium replacement. After 7 days, hiPSC were dissociated using 1 mL/well Versene solution (Life Technologies) at 37°C for 5 minutes and reseeded as monolayers on Matrigel-coated 6-well plates in StemMACs iPSC Brew XF medium. After 2 days, on day 0 of differentiation (when monolayers reached 90% confluence), cells were washed with Hanks' Balanced Salt Solution Ca^{2+}, magnesium, no phenol red (Thermo Fisher Scientific). The medium was changed to Roswell Park Memorial Institute (RPMI) supplemented with B27 minus insulin (Thermo Fisher Scientific) containing 12 µM CHIR99021 (Miltenyi Biotec). On day 1, the medium was changed to RPMI supplemented with B27 minus insulin. On day 3, the medium was changed to RPMI supplemented with B27 minus insulin, containing 5 µM the small molecule Wnt inhibitor IWP4 (Stemgent). On day 5, the medium was changed to RPMI supplemented with B27 minus insulin. Finally, from day 7 onwards, the medium was changed to RPMI supplemented with B27 complete supplement, RPMI+B27 medium (Thermo Fisher Scientific).

On day 31 of directed differentiation, cell cultures were trypsinized and hiPSC-cardiomyocytes (CM) were purified using magnetic-activated cell sorting (MACS) to target and deplete non-myocytes from the cell suspension, resulting in cardiomyocyte enriched to ~98% purity (Miltenyi Biotec, iPSC-CM isolation kit, human). Purified hiPSC-CM were washed with a Ca^{2+}-free, magnesium-free, phenol red-free solution (Thermo Fisher Scientific) and dissociated using 1 mL of 0.25% Trypsin/EDTA (Thermo Fisher Scientific) per well. Dissociated cells were then mixed with 6 mL of EB20 medium containing 80% DMEM/F12) (Thermo Fisher Scientific), 0.1 mM Non-Essential Amino Acids (Thermo Fisher Scientific), 1mM L-Glutamine (Thermo Fisher Scientific), 0.1 mM β-mercaptoethanol (Thermo Fisher Scientific), 20% Fetal Bovine Serum (Corning), and 10 µM Blebbistatin (Sigma-Aldrich). After collecting the cells, a 70-µm strainer was used, and cells were then centrifuged at 200 × g for 5 minutes

at room temperature. After removing the supernatant, 2 mL of MACS Buffer (containing PBS, 0.5% BSA and 2 mM Ethylenediaminetetraacetic acid) was added. Cells were centrifuged again at 200 × g for 5 minutes at room temperature. The cell pellet was resuspended in 80 µL MACS Buffer, mixed with 20 µL of non-myocytes-depletion cocktail (-Biotin-conjugated) primary antibody and incubated on ice for 5 minutes. After primary antibody incubation, 2 mL of MACS Buffer was added, and cells were gently resuspended, followed by centrifugation at 200 × g for 5 minutes at room temperature. The excess primary antibody was aspirated, and cells were resuspended in 80 µL of MACS Buffer, mixed with anti-Biotin magnetic microbeads (secondary antibody), and incubated on ice for 10 minutes. After the secondary antibody incubation, cells were mixed with 1 mL of MACs Buffer and passed through a 70-µm strainer before being processed using an autoMACS Pro Separator to purify hiPSC-CM and non-myocyte populations. The purified hiPSC-CM fractions were centrifuged, the supernatant was aspirated, and the purified hiPSC-CM were resuspended in EB20 media with 5 µM of a ROCK inhibitor. Finally, cells were plated at a density of 300-350k cells in a 12-well plate on 22 mm × 22 mm cut Matrigel-coated (100 µg/mL in DMEM/F12 media) polydimethylsiloxane for 14 days.

Optical mapping of human induced pluripotent stem cell-derived cardiomyocyte monolayers

Optical mapping of transmembrane voltage changes was performed in hiPSC-CM monolayers to study the electrophysiological effects of IL-6 on conduction velocity, action potential duration and reentry formation. Optical recordings of transmembrane voltage changes were obtained after dye loading with a voltage-sensitive dye (FluoVolt, Thermo Fisher Scientific). Monolayers were excited with two blue light-emitting diodes (CBT-90 Blue; Luminus Devices Inc., MA, USA) through an excitation filter centered at 480 nm (AT480/30x; Chroma Technology Corp., VT, USA), and the emitted light was collected through an emission filter centered at 525 nm (ET525/50m; Chroma Technology Corp.). Recordings were acquired over 6 seconds at 300 frames per second using an Evolve 128 Electron Multiplying Charge-Coupled Devices (EMCCD) camera (Teledyne Photometrics, AZ, USA). Treated monolayers were exposed to 200 pM of IL-6 (Thermo Fisher Scientific) for 6 hours before optical recordings. This concentration was based on average IL-6 concentration in tissue samples from driver regions (Figure S14A). Comparisons were made with monolayers exposed to IL-6 solvent. Electrophysiological measurements were taken during programmed focal biphasic stimulation at the edge of the monolayer (2 ms duration and 7–10 V amplitude) at different cycle lengths (900, 800, 600, 550 and 500 ms).

Finally, the raw datafiles in .tiff format were imported into ElectroMap, a MATLAB-based software designed for the analysis of electrophysiological datasets.[54] Using activation maps, conduction velocity was calculated with a multi-vector approach, where the local conduction speed and direction were calculated using the polynomial method of Bayly *et al.*[55] Phase movies were obtained by means of Hilbert transformation of the optical action potentials.

***Ex vivo* optical mapping of transmembrane voltage changes in whole heart preparations**

Optical imaging of transmembrane voltage changes on the left atrial surface was performed in 4 hearts with infarct-related substrate and 4 healthy controls. After cardiac surgery and euthanasia, explanted hearts were immersed in cold (4ºC) hyperkalemic Tyrode's solution (composition in mM: NaCl 130, $NaHCO_3$ 24, NaH_2PO_4 1.2, $MgCl_2$ 1, KCl 12, Glucose 5.6, $CaCl_2$ 1.8, and albumin 0.04 g/L). Isolated hearts were cannulated on the ascending aorta and connected to a constant-flow Langendorff-perfusion system with oxygenated (O_2-CO_2, 95/5%) Tyrode's solution (composition in mM: NaCl 130, $NaHCO_3$

24, NaH_2PO_4 1.2, $MgCl_2$ 1, KCl 4, Glucose 5.6, $CaCl_2$ 1.8, and albumin 0.04 g/L) at a flow-rate of 200-240 mL/min. Heart preparation for optical imaging included an atrial trans-septal puncture and sealing of all the vein orifices except the inferior vena cava, which was connected to an open-end cannula to control the intra-atrial pressure and outflow, as reported elsewhere.[4] Trans-septal puncture and further interatrial septum opening was performed at the fossa ovalis to enable equilibration of intracavitary pressures in all chambers. Then, the heart was submerged inside a 25-litre tank filled with warm saline at 37ºC. For imaging acquisition, a high-speed Complementary Metal-Oxide-Semiconductor (CMOS) camera (IDS Imaging Development Systems GmbH, Germany) was programmed to record 120x160 superpixels at 400 frames/second. Hearts were loaded with 600 µL of di-4-ANEQ(F)PTEA stock dye solution (10 mg di-4-ANEQ(F)PTEA [University of Connecticut School of Medicine, USA] dissolved in 3 mL of pure ethanol) diluted in 10 mL of Tyrode's solution, and delivered slowly (without recirculation) over a 1-minute period through an injection port upstream the aortic cannula. Blebbistatin (Enzo Life Sciences, USA) was added into the Tyrode´s solution to get a final concentration of 10 µM. Two red light-emitting-diodes (LEDs) (CBT-90-RX; Mouser Electronics, Mansfield, TX, USA) collimated with a plano-convex lens and filtered from 632–652nm (ZET642/20x; Chroma Technology Corp, Bellows Falls, VT, USA) were mounted on the left and right side of the camera for excitation. Fluorescence emission light from the heart was passed through a custom-made emission filter (passing above 700 nm; Chroma Technology Corp.) and then collected with a fast camera lens (Fujinon DF6HA-1S; RMA Electronics Inc., Hingham, MA, USA). Before imaging, ventricular fibrillation was induced to minimize the residual ventricular motion after Blebbistatin. Optical movies were recorded during atrial pacing from the left atrium at two S1 basic drive cycle lengths (500 and 240 ms).

Action potential duration analysis was performed offline using custom-made software (MathWorks Inc., USA). We used a validated method to obtain repolarization times from optical data at the time of $(d^2V/dt^2)_{max}$.[56] Briefly, activation time of each pixel was calculated at the time of $(dV/dt)_{max}$ after suitable preconditioning (cone-shaped kernel time-space smoothing) and filtering (zero-phased 50th-order low-pass Butterworth filter). Before determining the time of $(d^2V/dt^2)_{max}$ for each pixel, we smoothed the optical action potentials with local regression using weighted linear least squares and a 2nd degree polynomial model, order 100.

Action potential duration maps were calculated as the difference between activation and repolarization times at each pixel. Conduction velocity was estimated according to a polynomial fitting method as described elsewhere.[55] Briefly, spatial coordinates (*X*, *Y*) and activation times (*t*) were adjusted to a cubic spline surface, whose gradient provided the local direction and magnitude of conduction velocity.

Supplementary Figures and Lengends

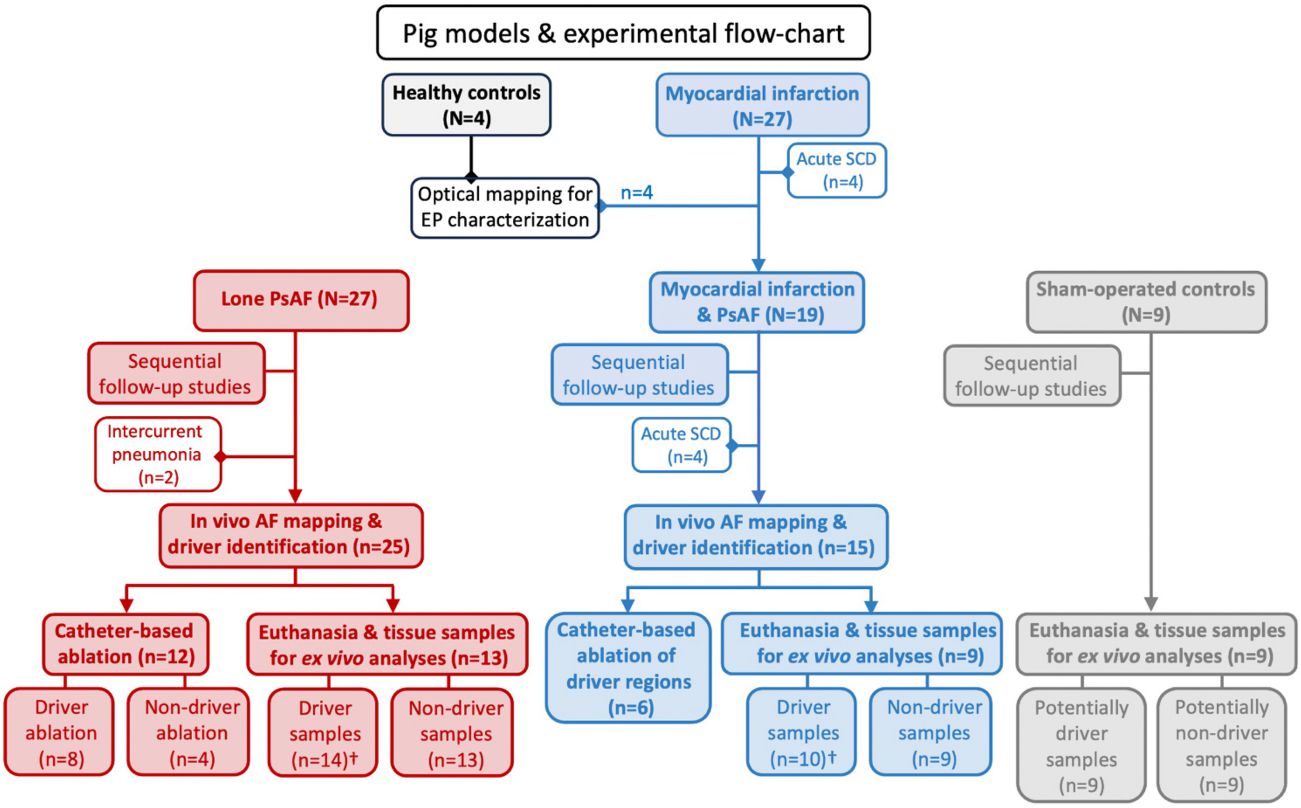

Figure S1. Experimental workflow and schematic outline of the animal models included in the study. Pigs with lone persistent atrial fibrillation (PsAF) (N=27, red boxes): two animals died during follow-up due to intercurrent pneumonia. After *in vivo* high-density electroanatomical mapping, a subgroup of twelve animals underwent catheter-based radiofrequency ablation of driver regions (n=8) or non-driver regions (n=4). Thirteen animals were used to *ex vivo* analysis after euthanasia. Pigs with underlying infarct-related substrate (N=27, blue boxes): four animals died early after myocardial infarction (MI) due to sudden cardiac death (SCD). Four surviving animals with MI were used for optical mapping to study baseline electrophysiological (EP) differences between the left atrium of infarcted animals and healthy controls (n=4). Nineteen pigs were used to generate the PsAF model in animals with the underlying infarct related substrate. In this group, four more animals died during follow-up of the AF protocol. The surviving animals with PsAF and infarct-related substrate (MI-PsAF) (n=15) were divided into a subgroup that underwent catheter-based radiofrequency ablation of driver regions (n=6) and another subgroup that was used for *ex vivo* analyses after euthanasia (n=9). Sham-operated controls (N=9, grey boxes) were used for *ex vivo* analyses after euthanasia and comparisons with the animals from the lone PsAF and MI-PsAF models. For the analysis of *ex vivo* samples after euthanasia, more than one driver region could be included per pig (†).

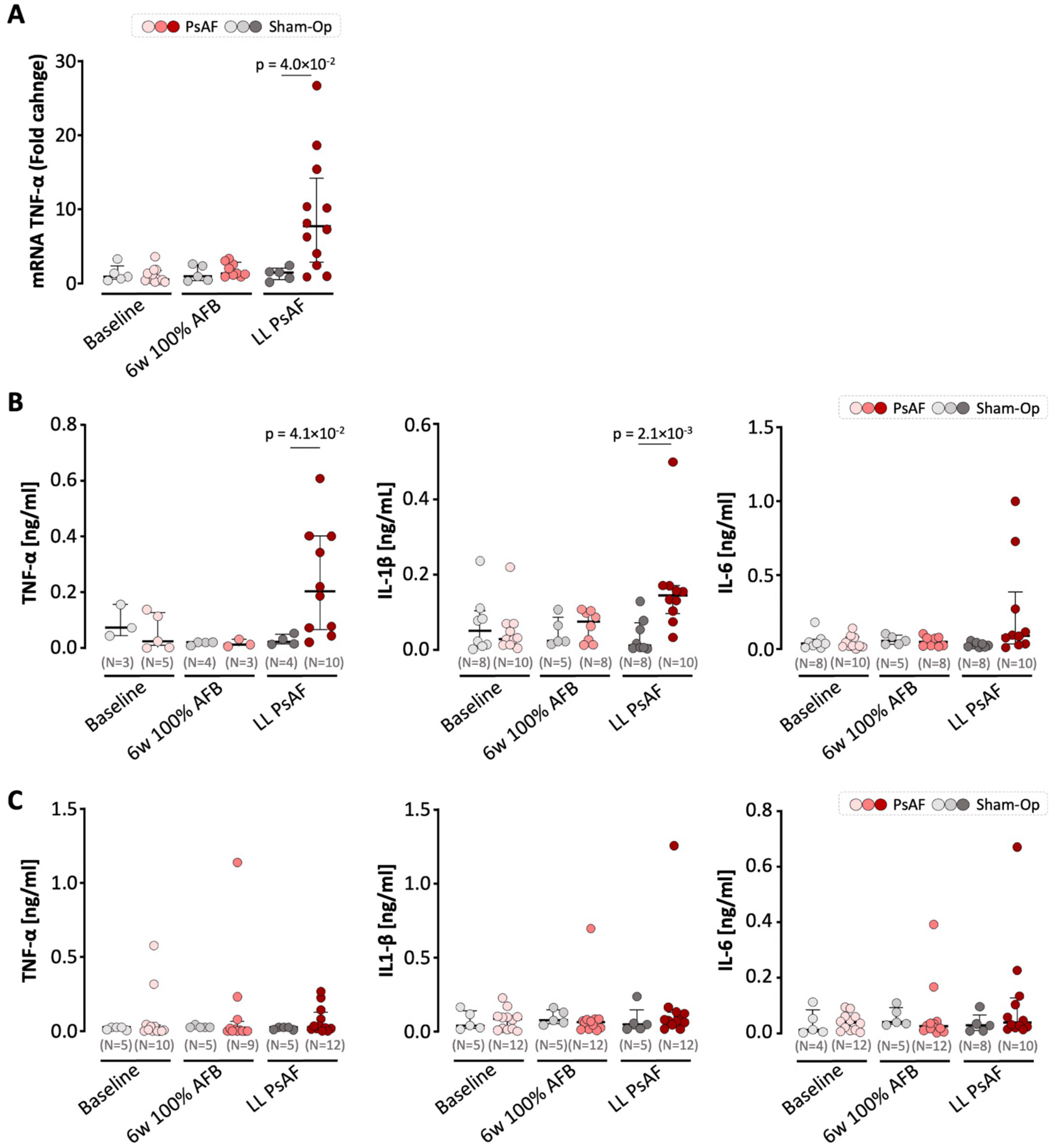

Figure S2. Analysis of inflammatory biomarkers in tissue and plasma samples during atrial fibrillation progression in pigs without infarct-related substrate. A, Quantification of mRNA expression of tumor necrosis factor alpha (TNF-α) in right atrial endocardial biopsies at baseline, at 6 weeks of 100% atrial fibrillation burden (6w 100% AFB) and at the end of follow-up during long-lasting lone persistent atrial fibrillation (LL PsAF) (n=12). Red color-graded circles indicate PsAF animals. Right atrial biopsies from sham-operated controls (grey color-graded circles, n=5) at equivalent follow-up times were used for comparisons. **B, C,** Quantification of TNF-α, interleukin-1β (IL-1β) and interleukin-6 (IL-6) in plasma samples from the coronary sinus (**B**) and the femoral vein (**C**) in pigs with lone AF at baseline, at 6w 100% AFB and at the end of follow-up during LL PsAF. Samples from sham-operated controls were also taken at equivalent follow-up times for comparisons. In **A**, **B** and **C**, the multiple Mann-Whitney *U* test was used with the Holm-Šídák correction for multiple testing

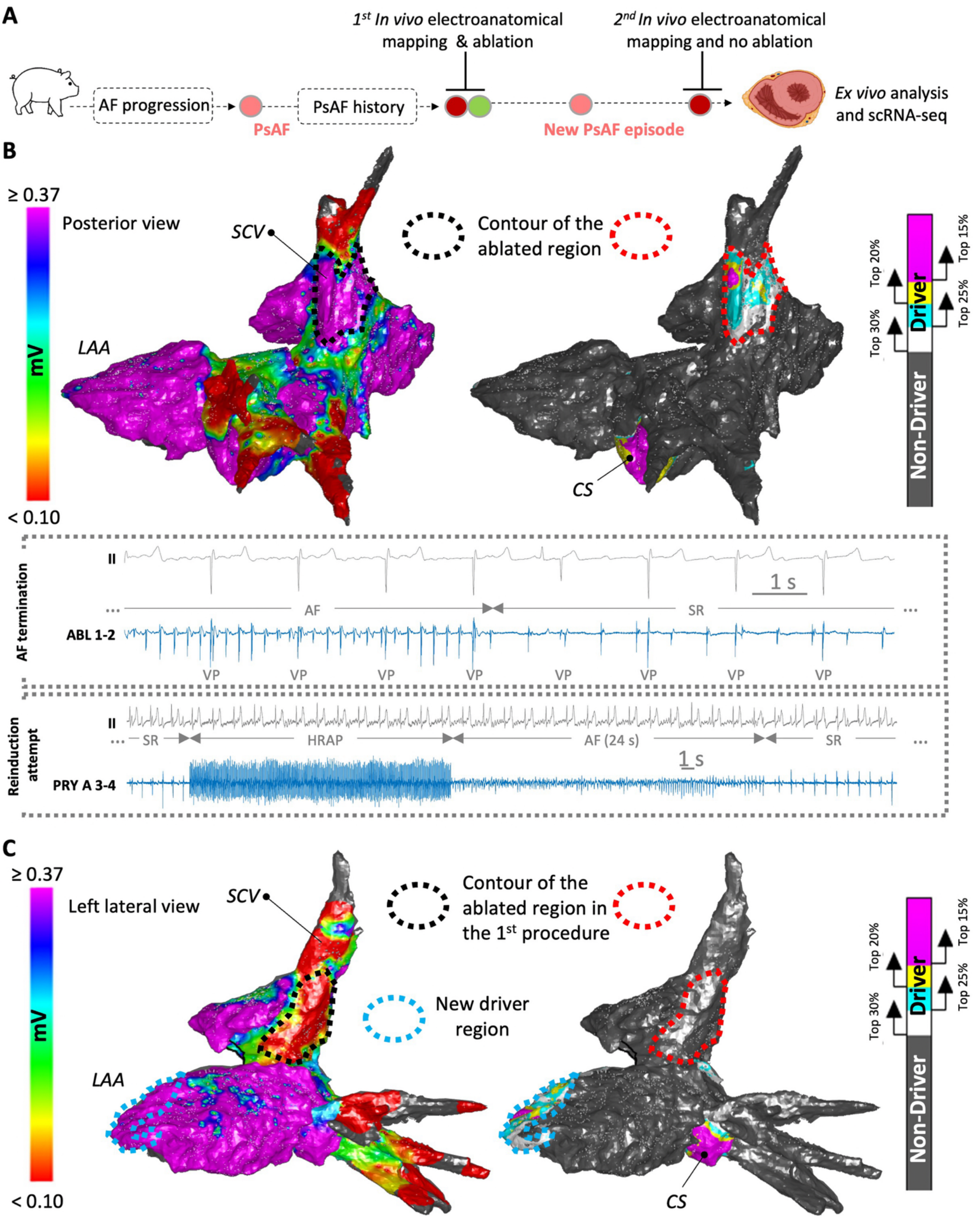

Figure S3. ***In vivo*** **mapping and driver identification in a pig with long-lasting lone persistent atrial fibrillation that underwent two invasive procedures.** **A**, Schematic outline of the experimental protocol. **B**, Top left, bipolar voltage map showing the distribution of voltage-derived scar regions (<0.10 mV), healthy regions (≥0.37 mV) and heterogenous regions (between 0.10 and 0.37 mV) in the sample case at the time of the first mapping procedure. Tissue with normal voltage values during atrial fibrillation (AF) is displayed in magenta. The rest of colors represent the transition to the lowest voltage regions, compatible with a higher

degree of underlying scar. Top right, driver map in which driver regions are highlighted in magenta/yellow/cyan/white based on their median instantaneous frequency modulation (iFM) hierarchy. Thus, the highest median iFM values were identified in regions colored in magenta, yellow, cyan and white, corresponding to top 15%, top 20%, top 25% and top 30% of median iFM values; i.e. driver regions. Conversely, the lowest median iFM values were identified in regions colored in dark grey (from 0-70% of median iFM values; i.e. non-driver regions). Persistent AF (PsAF) terminated after 48 min of radiofrequency energy delivery at the largest driver location in the junction between the posterior side of the superior cava vein (SCV) and the upper part of the posterior right atrium (the dotted red line on the driver map displays the ablated region contour). Additional ablation lesions were applied at a second driver location in the coronary sinus (CS) until a total of 50 minutes of radiofrequency energy delivery was completed (maximum ablation time established per protocol). Bottom, surface and intracardiac tracings showing AF termination and no possibility to induce sustained AF episodes (sustained for >10 minutes) after burst pacing. The day after the ablation procedure, the high-rate atrial pacing protocol was resumed aiming to progressively develop a new PsAF episode. **C**, Voltage and driver maps from the second mapping procedure 63 days after the first procedure and after 41 days in self-sustained PsAF. The maps identified a new driver region in the left atrial appendage (LAA, dotted cyan contour), not present in the first procedure. As expected, the maps also confirmed that the posterior side of SCV and the upper part of the posterior right atrium were no longer a driver and displayed low voltage values compatible with post-ablation scar (dotted black and red contours on the voltage and driver maps, respectively). We also documented the persistence of the small driver region in the CS, which received less ablation lesions. HRAP: high-rate atrial pacing. PRY A indicates signal recordings from bipoles of the spline A of multipolar mapping catheter. SR: sinus rhythm. VP: ventricular pacing.

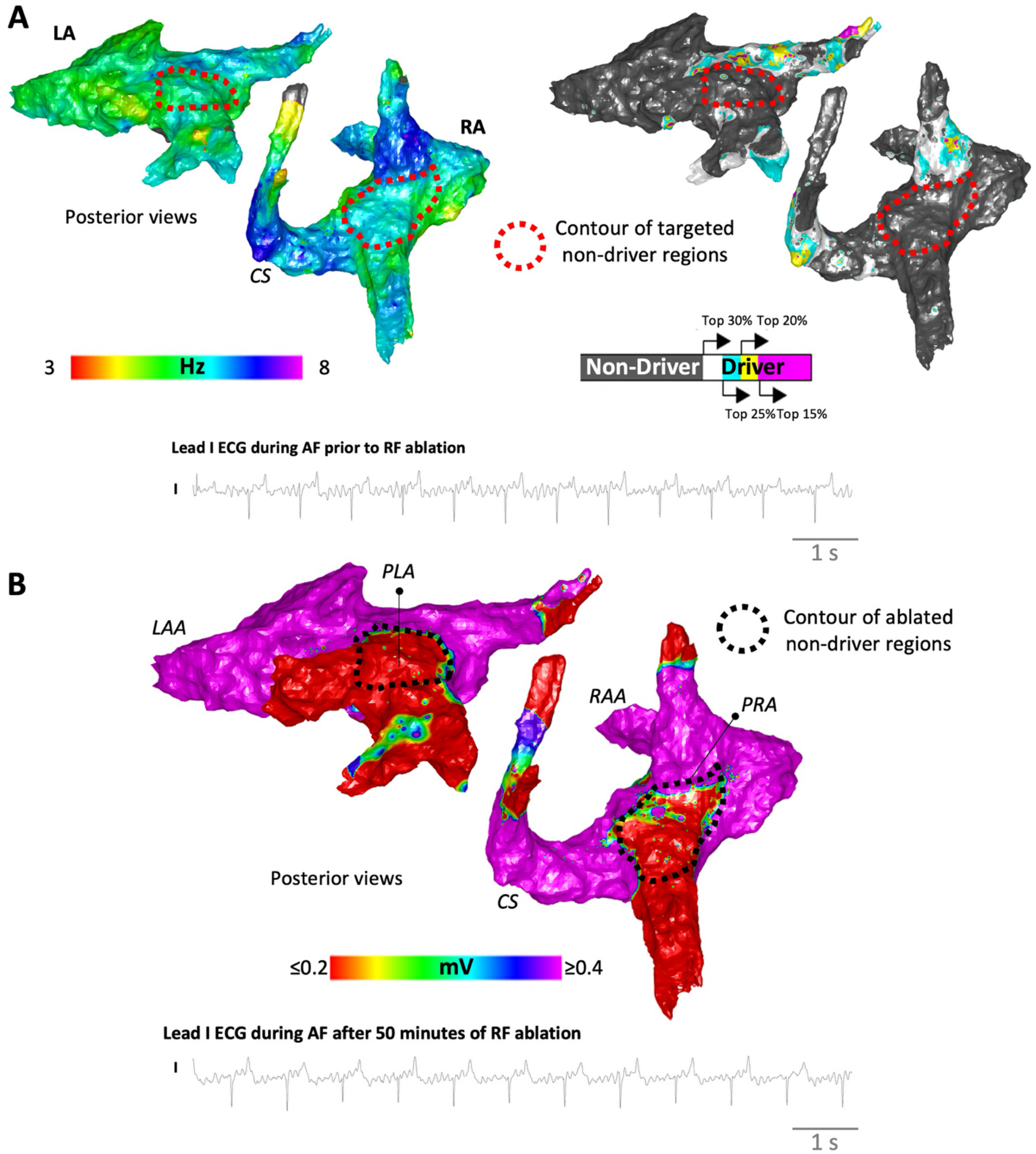

Figure S4. ***In vivo*** **mapping and ablation of non-driver regions in a pig with long-lasting lone persistent atrial fibrillation. A,** Left, median instantaneous frequency modulation (iFM) map, in which regions with higher median iFM values (driver regions) are displayed in dark blue and magenta colors. Right**,** driver map in which atrial regions activating faster than their surroundings and within the top 30% median iFM values (drivers) are color-coded in magenta, yellow, cyan and white, which correspond to top 15%, top 20%, top 25% and top 30% of median iFM values. Conversely, regions with lower median iFM values (from 0-70% of median iFM values; i.e. non-driver regions) were colored in dark grey. The targeted non-driver region for ablation is indicated with a dotted red contour. Bottom, sample surface lead I electrocardiogram (ECG) tracings showing sustained atrial fibrillation (AF) before radiofrequency (RF) ablation. **B**, Bipolar voltage map showing the distribution of voltage-derived scar regions (<0.10 mV), healthy regions (≥0.37 mV) and heterogenous regions (between 0.10 and 0.37 mV) after RF ablation at non-driver locations on the posterior left atrium (PLA) and the posterior right atrium (PRA). Atrial regions with normal voltage values during AF are displayed in magenta.

The remaining colors represent the transition to the lowest voltage regions, which are compatible with a higher degree of underlying scar. Persistent AF (PsAF) did not terminate after 50 minutes of RF energy delivery. The ablated non-driver region is indicated with a dotted black contour on the voltage map. Bottom, sample surface lead I ECG tracings showing sustained AF after ablation. CS: coronary sinus; LA: left atrium; LAA: left atrial appendage; RA: right atrium; RAA: right atrial appendage.

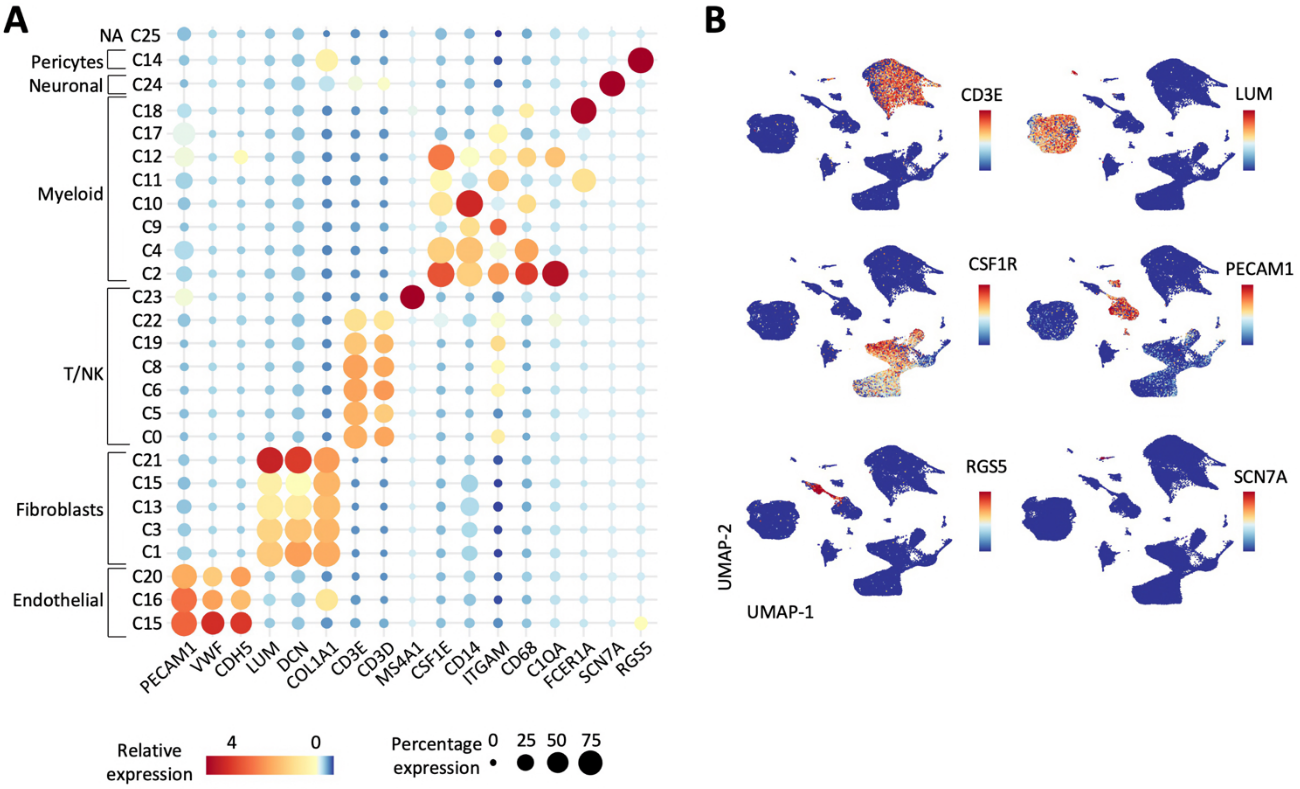

Figure S5. Expression of canonical markers on non-myocyte cell populations. A, Seurat-generated dot plot showing representative canonical genes for each major identified cell population (endothelial cells, fibroblasts, T/natural killer cells [NK], myeloid cells, pericytes and neuron-like cells) in atrial tissue samples from pigs with long-lasting lone persistent atrial fibrillation and sham-operated controls (atrial samples included myocardial tissue from both atria). **B**, Seurat-generated feature plots illustrating distinctive expression of canonical markers for the 6 cell populations in **(A)**.

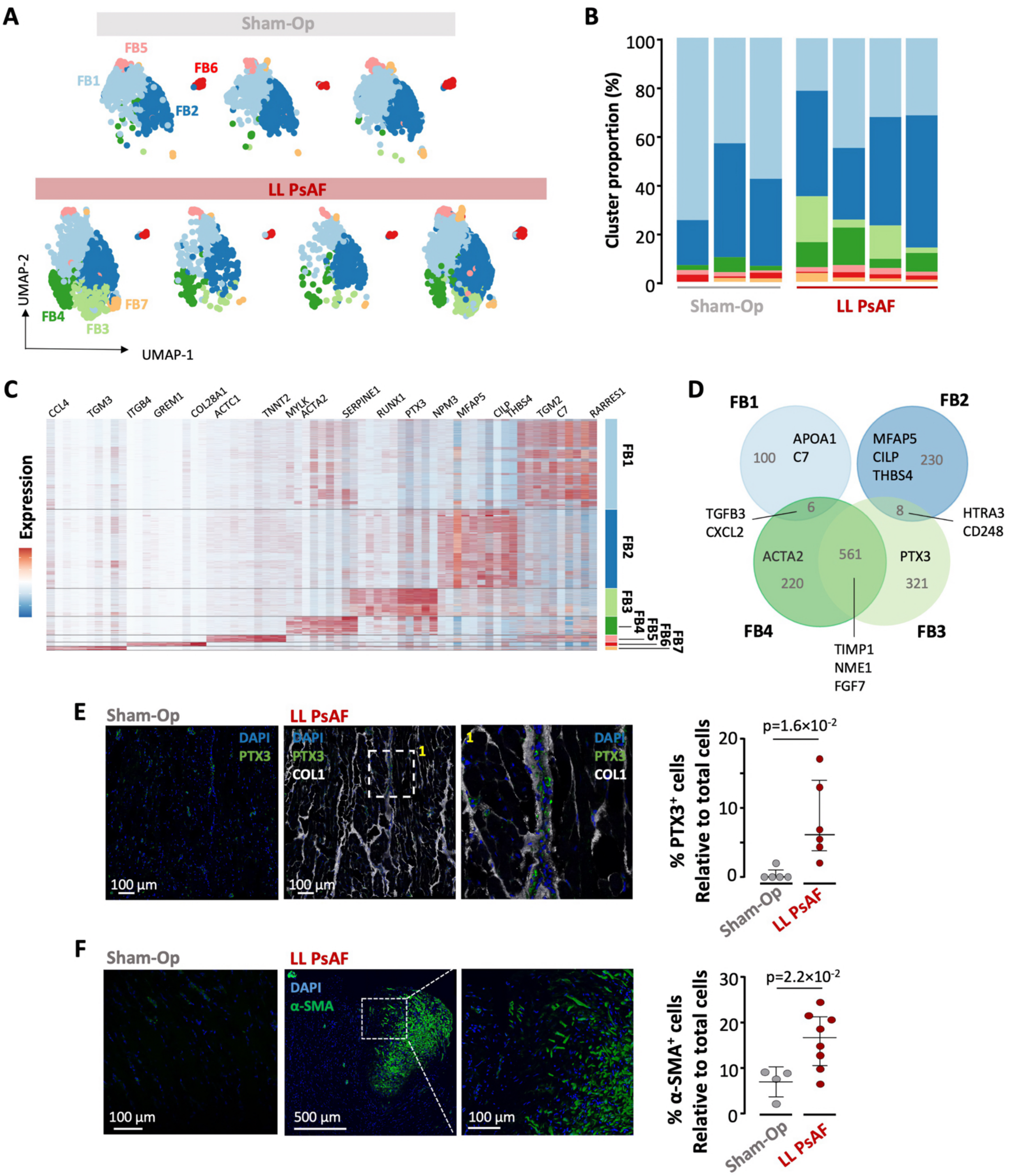

Figure S6. Distinguishing features of fibroblast clusters in the atria of pigs with lone persistent atrial fibrillation compared to sham-operated controls. A, Uniform Manifold Approximation and Projection (UMAP) plots in individual single-cell RNA sequencing (scRNA-seq) experiments from sham-operated controls (n=3) and long-lasting lone persistent atrial fibrillation animals (LL PsAF, n=4). The analysis of atrial samples included both atria. **B**, Cluster proportion analysis separated by model (sham-operated controls or LL PsAF) and biological replicates. **C**, Heat map of scaled expression of the top 15 marker genes for each fibroblast cluster. **D**, Venn diagrams showing the quantification of unique or shared differentially expressed genes (DEG) in FB1, FB2, FB3 and FB4 clusters. Selected genes are highlighted (log_2 fold change>0.5, adjusted p-value<0.05). **E**, Left, immuno-labeling showing expression of PTX3, collagen1 and DAPI (nuclei) in left atrial

LL PsAF samples. Right, quantification of PTX3-positive cells relative to total cells. **F**, Left, immuno-labeling of left atrial sections from sham-operated and LL PsAF animals stained with anti-αSMA antibody and DAPI (nuclei). Right, quantification of αSMA-positive cells relative to total cells. Data are shown as median and interquartile range. In (**E**) and (**F**), the two-sided unpaired Student´s t-test was used to assess statistical differences.

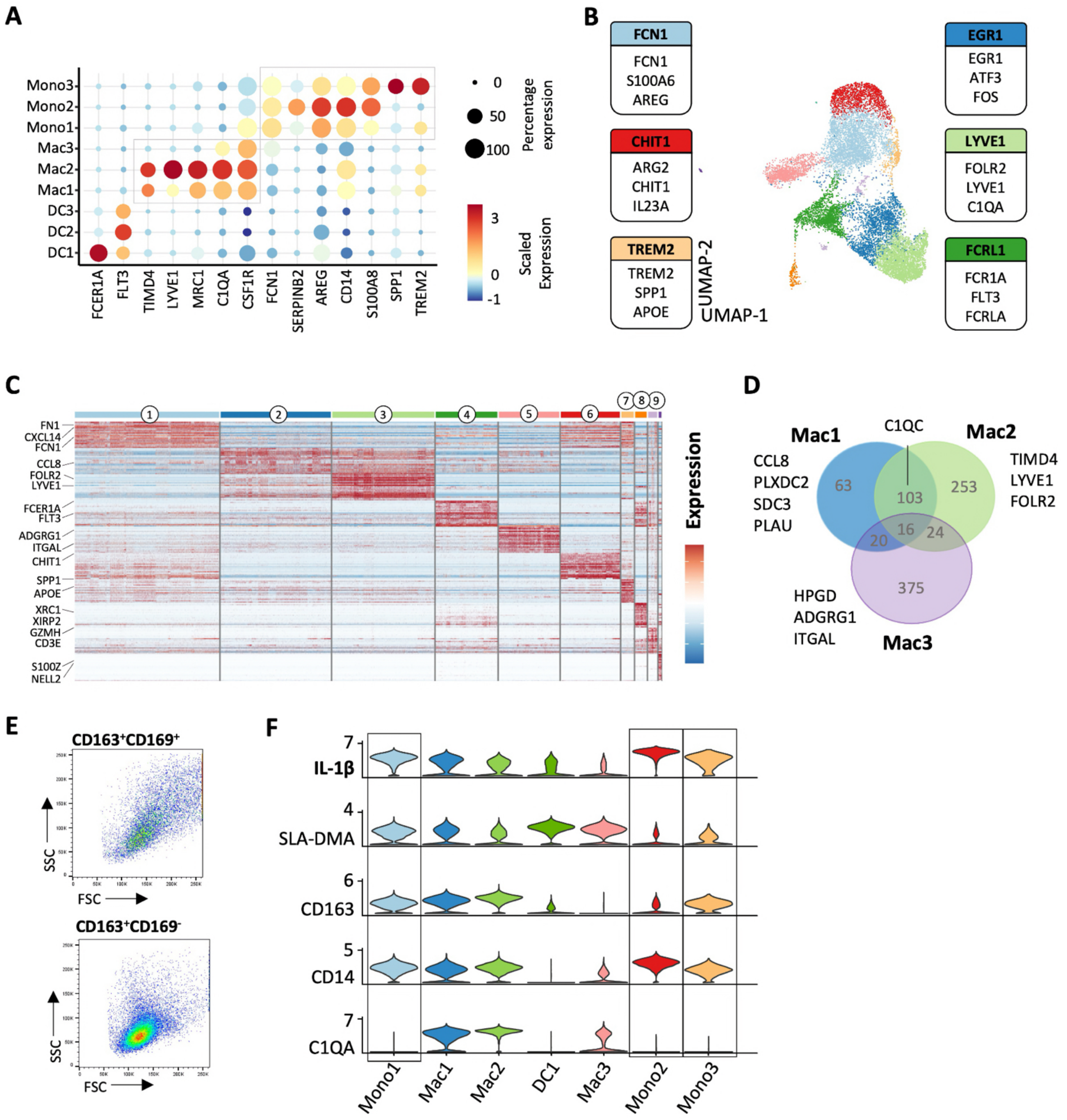

Figure S7. Characterization of myeloid clusters in the atria of animals with long-lasting lone persistent atrial fibrillation and sham-operated controls. A, Seurat-generated dot plot showing representative canonical genes in each myeloid cluster identified (3 clusters of macrophages [Mac], 3 clusters of monocytes [Mono] and 3 clusters of dendritic cells [DC]) in atrial samples from sham-operated controls (n=3) and long-lasting lone persistent atrial fibrillation animals (LL PsAF, n=4). The analysis of atrial samples included both atria; right atrial free wall, the coronary sinus with its adjacent posterior left atrium wall, and the superior vena cava. **B**, Uniform Manifold Approximation and Projection (UMAP) of single-cell RNA sequencing (scRNA-seq) data from myeloid cells. **C**, Heat map of scaled expression of the top 20 marker genes for each myeloid cluster. **D**, Venn diagrams showing the quantification of unique or shared differentially expressed genes (DEG) of Mac1, Mac2 and Mac3 clusters. Selected genes are highlighted ($\log_2$ fold change>0.5, adjusted p-value<0.05). **E**, Forward-side scatter of $CD163^{+}CD169^{+}$ cells reveals higher size and volume of cytoplasmic granules compared to $CD163^{+}CD169^{-}$ cells. **F**, Violin-plots showing gene expression levels (represented by log-transformed normalized counts) across myeloid clusters.

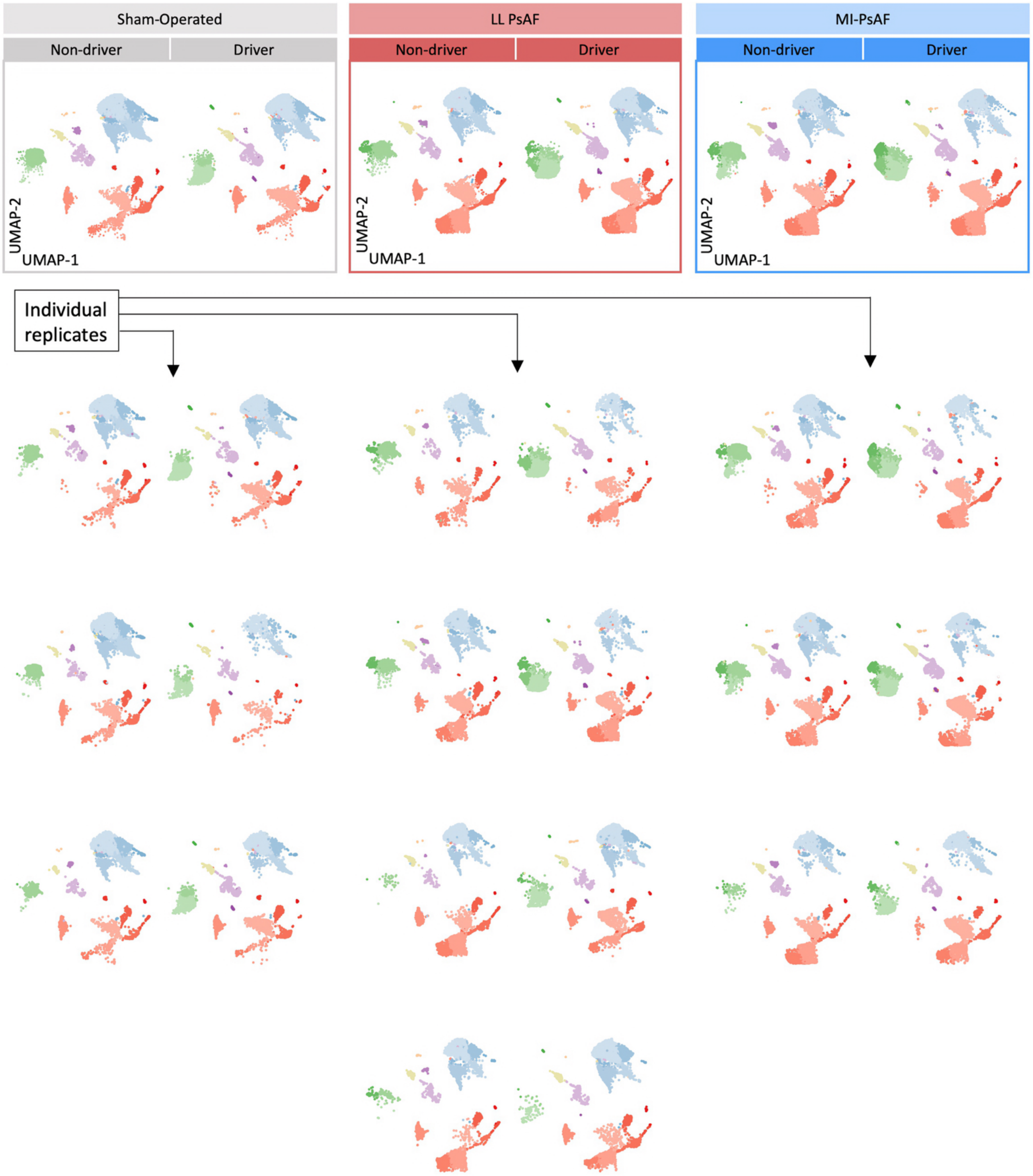

Figure S8. Consistency in single-cell RNA sequencing data across atrial regions and models. Uniform Manifold Approximation and Projection (UMAP) plots derived from individual single-cell RNA sequencing experiments of all non-myocyte cells separated by anatomical areas (driver and non-driver regions), model (sham-operated controls, long-lasting lone persistent atrial fibrillation [LL PsAF] and long-lasting PsAF with underlying infarct-related substrate [LL MI-PsAF]) and biological replicates. Potentially driver and non-driver regions in sham-operated animals were selected based on the functional relevance (driver prevalence) of the equivalent anatomical regions in LL PsAF animals (See Figure 1J).

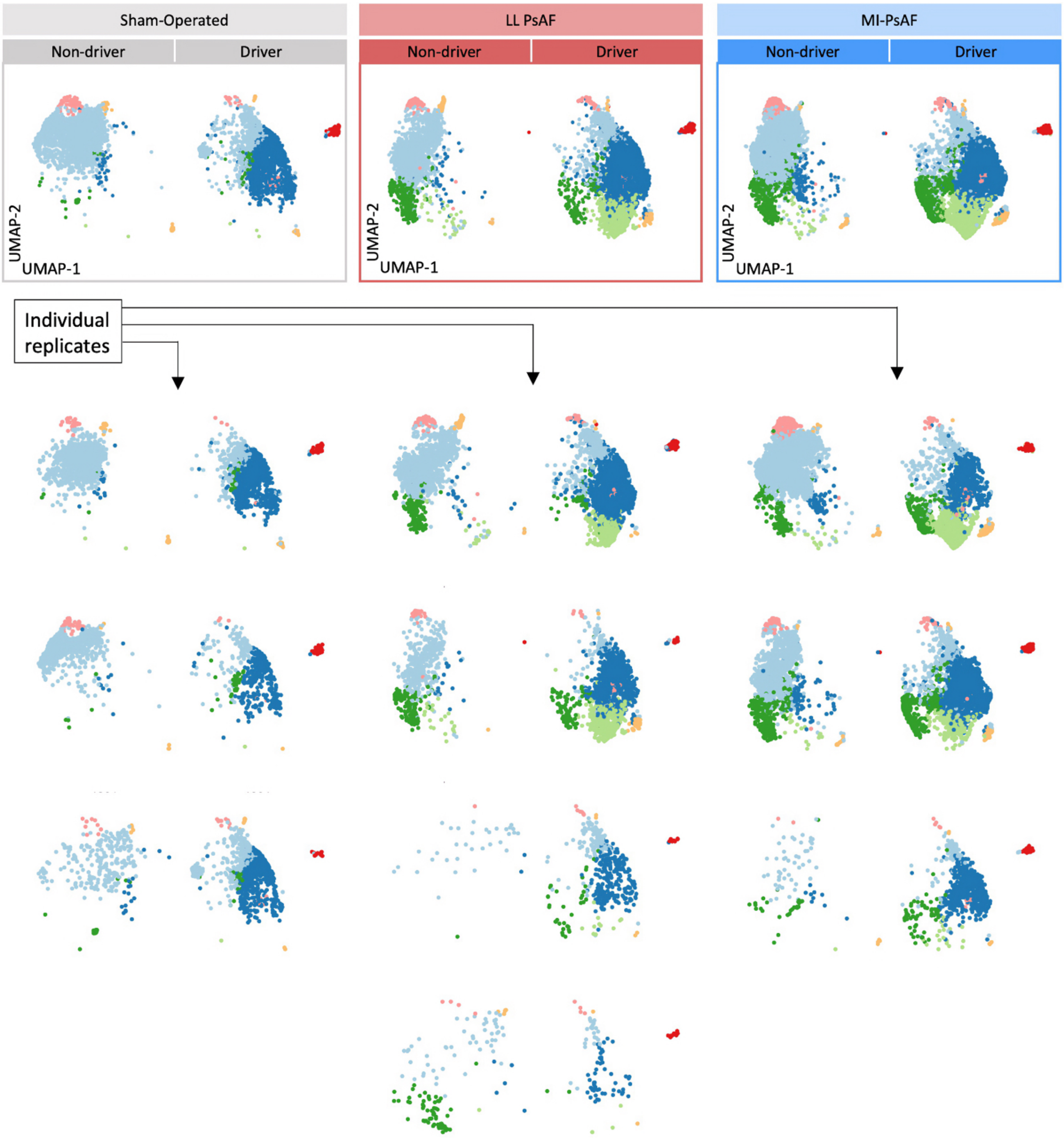

Figure S9. Consistency in single-cell RNA sequencing data across atrial regions and models for fibroblast clusters. Uniform Manifold Approximation and Projection (UMAP) plots derived from individual single-cell RNA sequencing experiments of fibroblast cells separated by anatomical areas (driver and non-driver regions), model (sham-operated controls, long-lasting lone persistent atrial fibrillation [LL PsAF] and long-lasting PsAF with underlying infarct-related substrate [LL MI-PsAF]) and biological replicates. Potentially driver and non-driver regions in sham-operated animals were selected based on the functional relevance (driver prevalence) of the equivalent anatomical regions in LL PsAF animals (See Figure 1J).

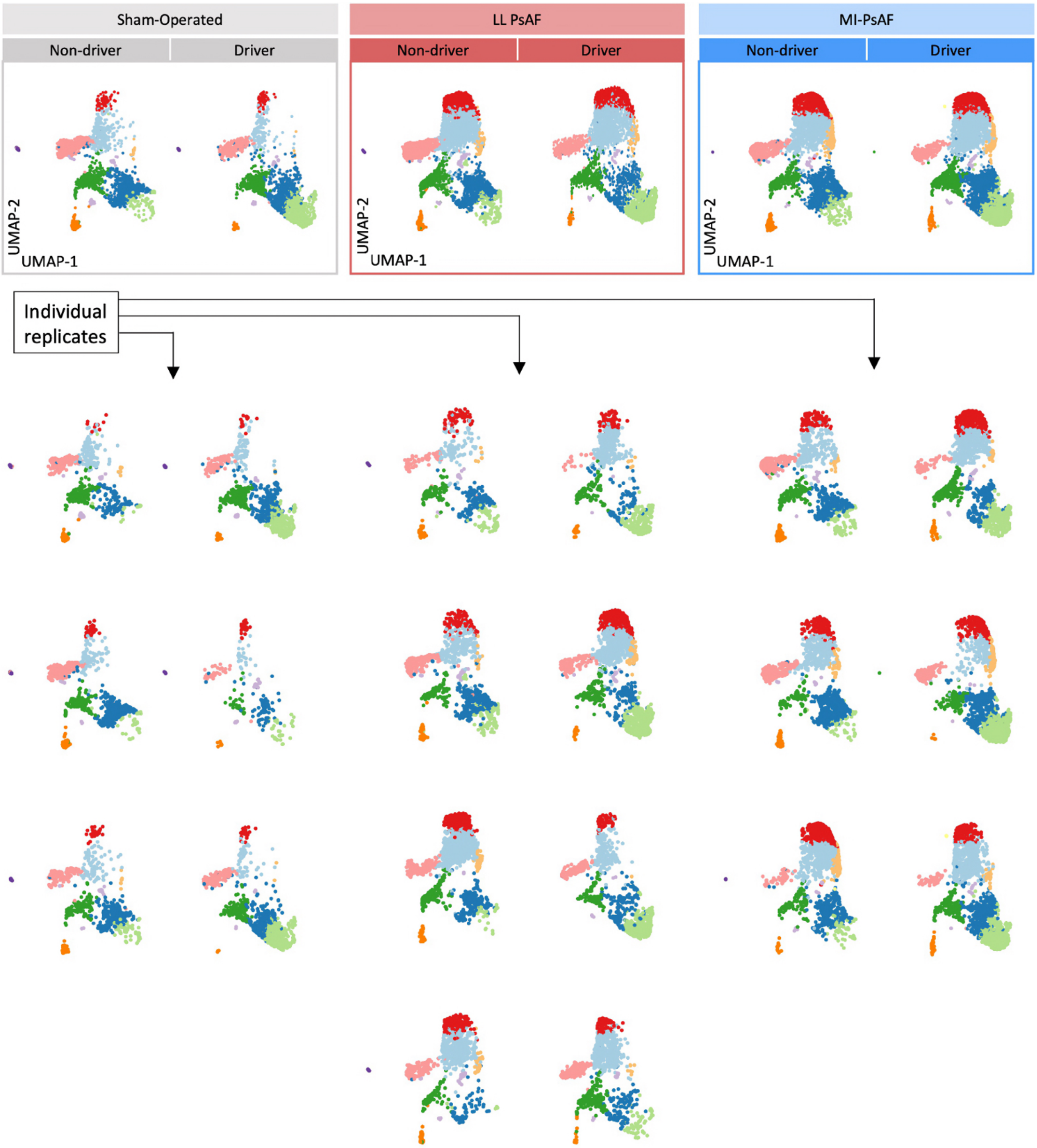

Figure S10. Consistency in single-cell RNA sequencing data across atrial regions and models for myeloid clusters. Uniform Manifold Approximation and Projection (UMAP) plots derived from individual single-cell RNA sequencing experiments of all myeloid cells separated by anatomical areas (driver and non-driver regions), model (sham-operated controls, long-lasting lone persistent atrial fibrillation [LL PsAF] and long-lasting PsAF with underlying infarct-related substrate [LL MI-PsAF]) and biological replicates. Potentially driver and non-driver regions in sham-operated animals were selected based on the functional relevance (driver prevalence) of the equivalent anatomical regions in LL PsAF animals (See Figure 1J).

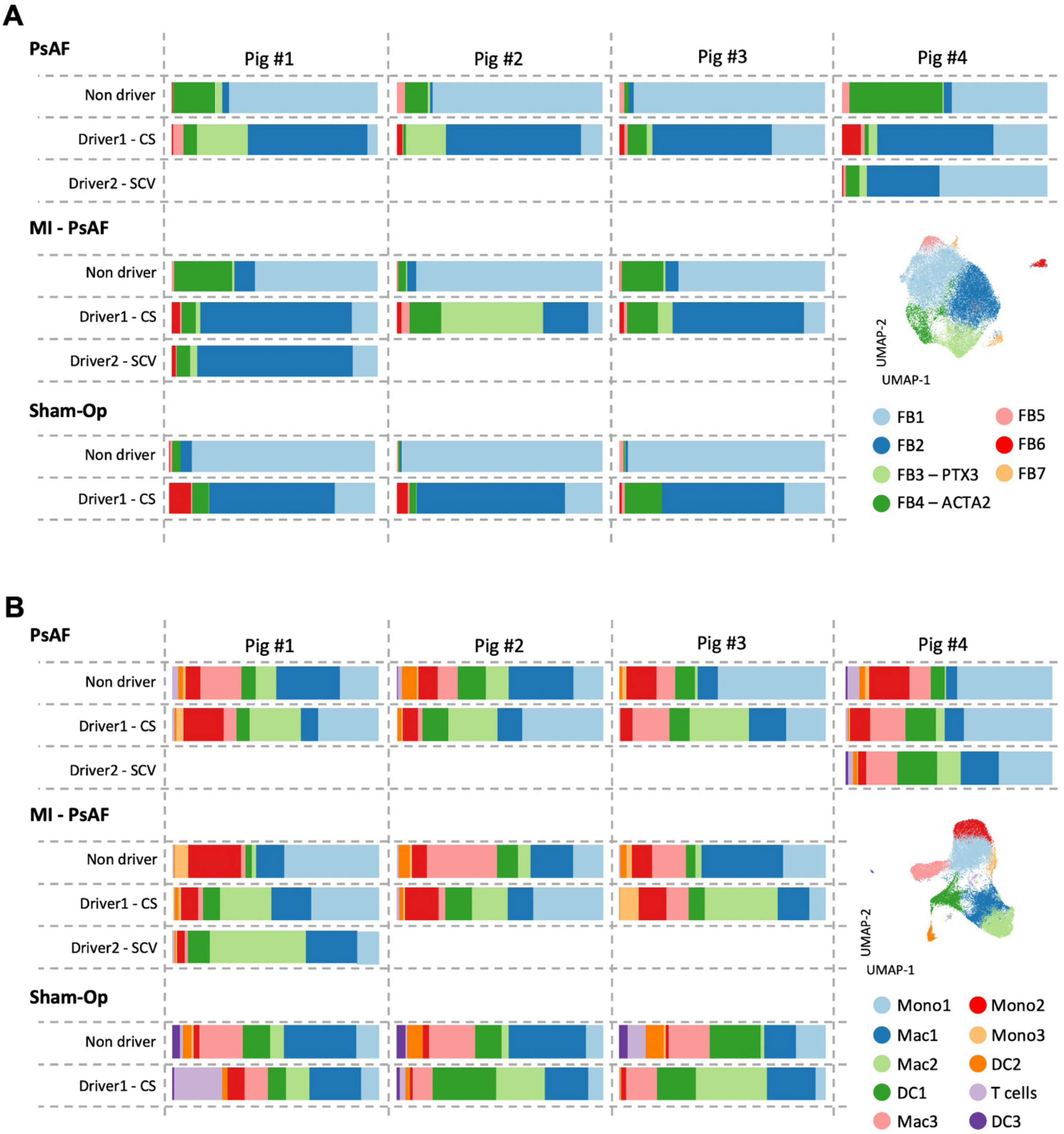

Figure S11. Distribution of cell populations across atrial regions separated by individual pigs. Data were generated from driver regions with the highest median instantaneous frequency modulation (iFM) values and a reproducible non-driver region (right atrial free wall) with the lowest median iFM values in experimental models of long-lasting lone persistent atrial fibrillation (LL PsAF) and LL PsAF with infarct-related substrate (LL MI-PsAF). Potentially driver and non-driver regions in sham-operated animals were selected based on the functional relevance (driver prevalence) of the equivalent anatomical regions in LL PsAF animals (See Figure 1J). **A**, distribution of fibroblast clusters. **B**, distribution of myeloid clusters. Additional analysis of a second driver region from the same pig is labelled as Driver2. CS: coronary sinus; SVC: superior vena cava; UMAP plots: Uniform Manifold Approximation and Projection plots.

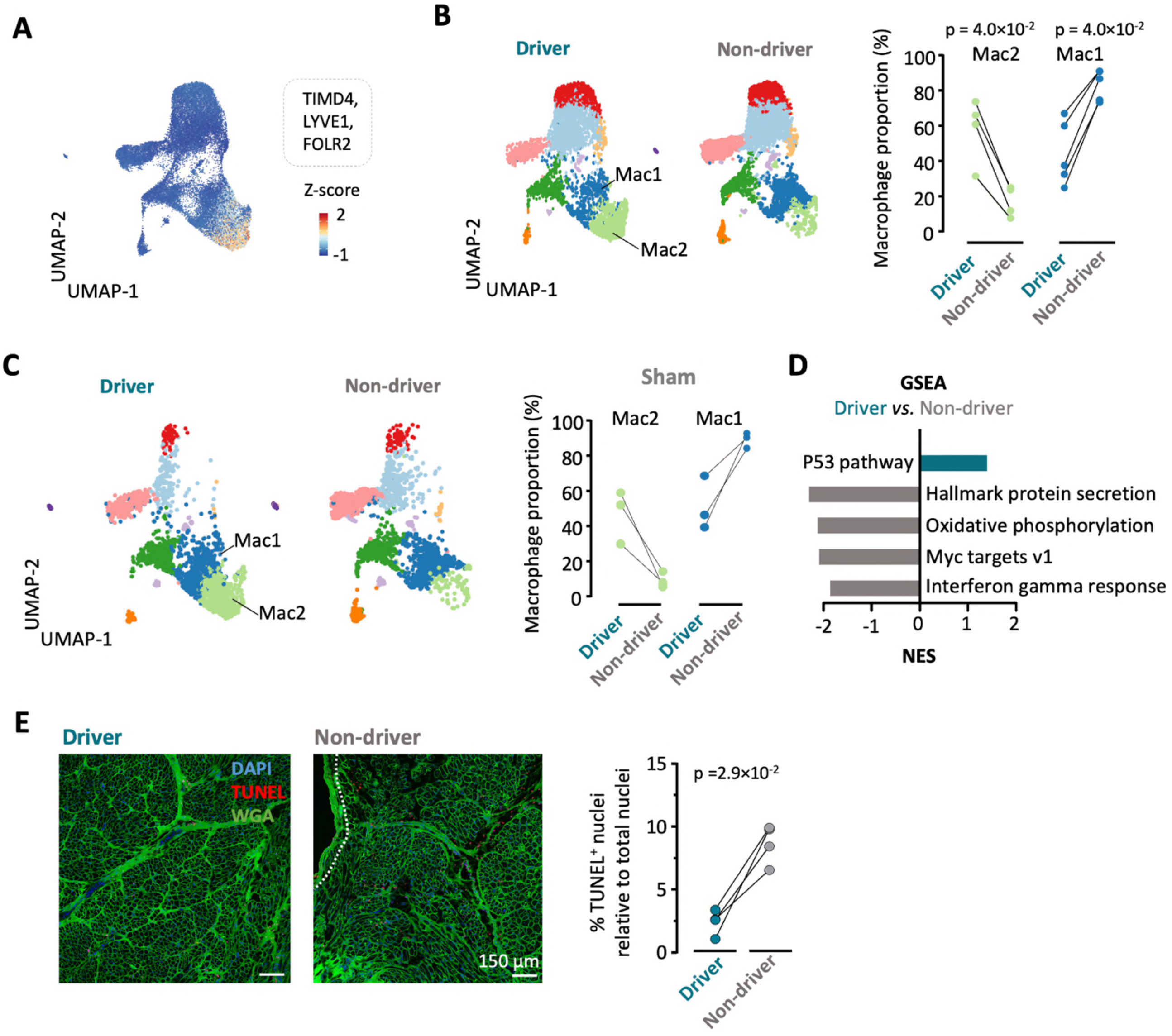

Figure S12. Expression of gene signatures associated with tissue resident macrophages and detection of TUNEL-positive cells in driver and non-driver regions of animal models with persistent atrial fibrillation. **A**, Z score feature plot of transcriptional signatures associated with tissue resident macrophages marked by the combination of TIMD4, LYVE1 and FOLR2 markers. **B**, Left, Uniform Manifold Approximation and Projection (UMAP) plots of myeloid clusters separated by anatomical areas (driver and non-driver regions) of animals with long-lasting lone persistent atrial fibrillation [LL PsAF]. Right, quantification of Mac1 and Mac2 cluster proportion relative to total macrophages showing that Mac2 cluster, expressing a protective tissue resident signature, was more predominant in driver regions of animals with LL PsAF. The opposite was true for Mac1 cluster. **C**, Left, UMAP plots of myeloid clusters separated by equivalent anatomical regions (driver and non-driver regions) in sham-operated controls. Right, quantification of cluster proportions as in **(B)**. **D**, Gene set enrichment analysis (GSEA) (Hallmarks and KEGG) showing upregulated pathways on genes ranked by log_2-FC between equivalent driver and non-driver regions of sham-operated pigs. **E**, Left, sample immunostaining of TUNEL-positive cells and confocal microscopy imaging of driver and non-driver regions of animals with LL PsAF (n=4). Right, quantification and comparison of TUNEL-positive cells between driver and non-driver regions. We systematically excluded the epicardial surface and all section margins (white dash lines) for TUNEL quantification to avoid edge artifacts. In **(B)**, **(C)** and **(E)**, the Wilcoxon signed-rank test was used to assess statistically significant differences. NES: Normalized Enrichment Score.

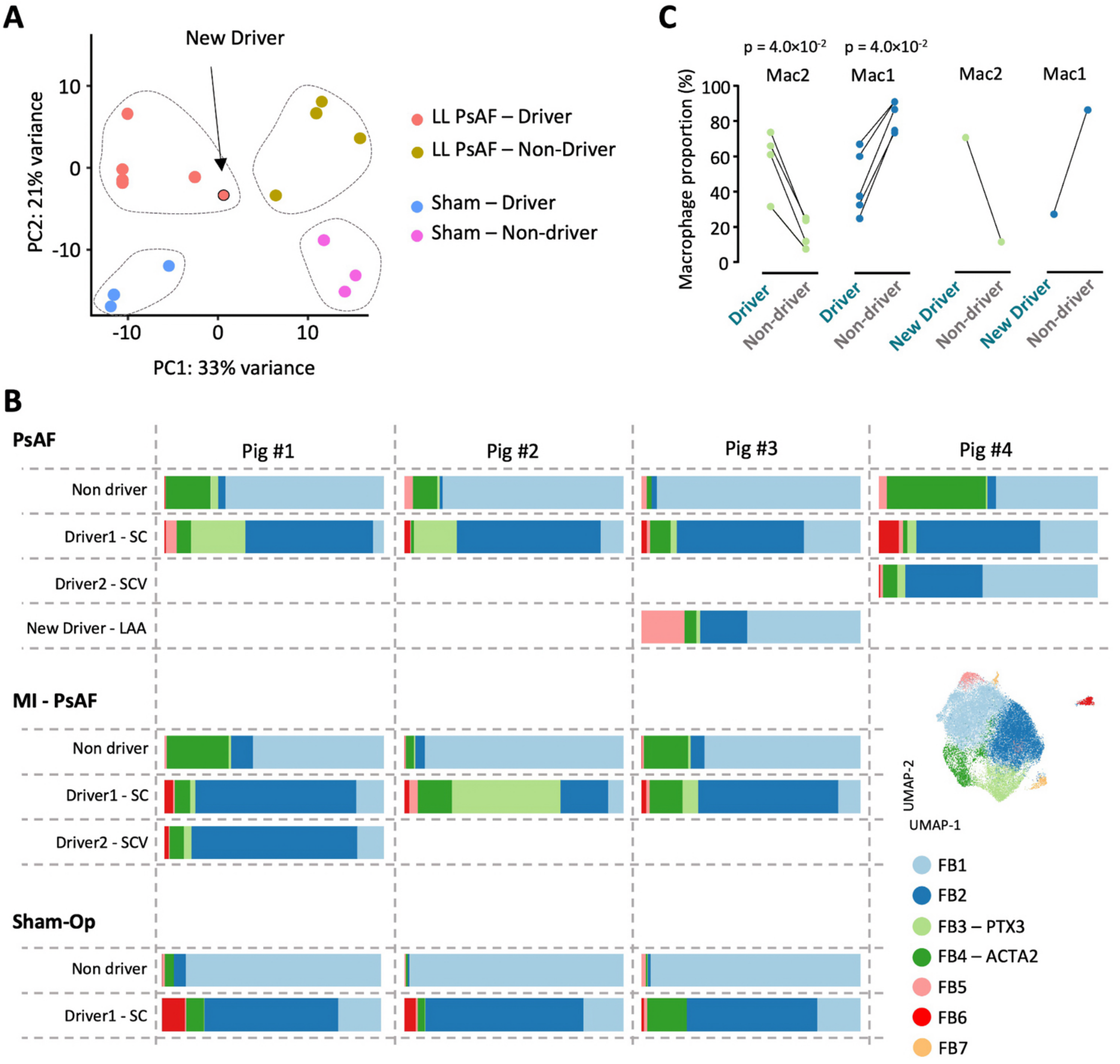

Figure S13. Single-cell RNA sequencing data of new drivers after catheter ablation of primary driver sites during persistent lone atrial fibrillation. A, Principal component analysis (PCA) plot of pseudo-bulk single-cell RNA sequencing (scRNA-seq) data colored by cardiac condition (sham-operated or long-lasting lone persistent atrial fibrillation [LL PsAF]) and region (driver, new driver and non-driver). **B**, Distribution of fibroblasts clusters across atrial regions separated by individual pigs. **C**, Quantification of Mac1 and Mac2 cluster proportions relative to total macrophages. The analysis shows that Mac2 cluster, expressing a protective tissue resident signature, was more predominant in driver regions of animals with LL PsAF. New driver region indicates a driver that appeared after a second mapping procedure (Figure S3). The Wilcoxon signed-rank test was used to assess statistically significant differences. Data were generated from driver regions with the highest median instantaneous frequency modulation (iFM) values and a reproducible non-driver region (right atrial free wall) with the lowest median iFM values in experimental models of LL PsAF and LL PsAF with infarct-related substrate (LL MI-PsAF). Potentially driver and non-driver regions in sham-operated animals were selected based on the functional relevance (driver prevalence) of the equivalent anatomical regions in LL PsAF animals (See Figure 1J). Additional analysis of a second driver region from the same pig is labelled as Driver2 or New Driver (left atrial appendage [LAA]). CS: coronary sinus; SVC: superior vena cava. UMAP plots: Uniform Manifold Approximation and Projection plots.

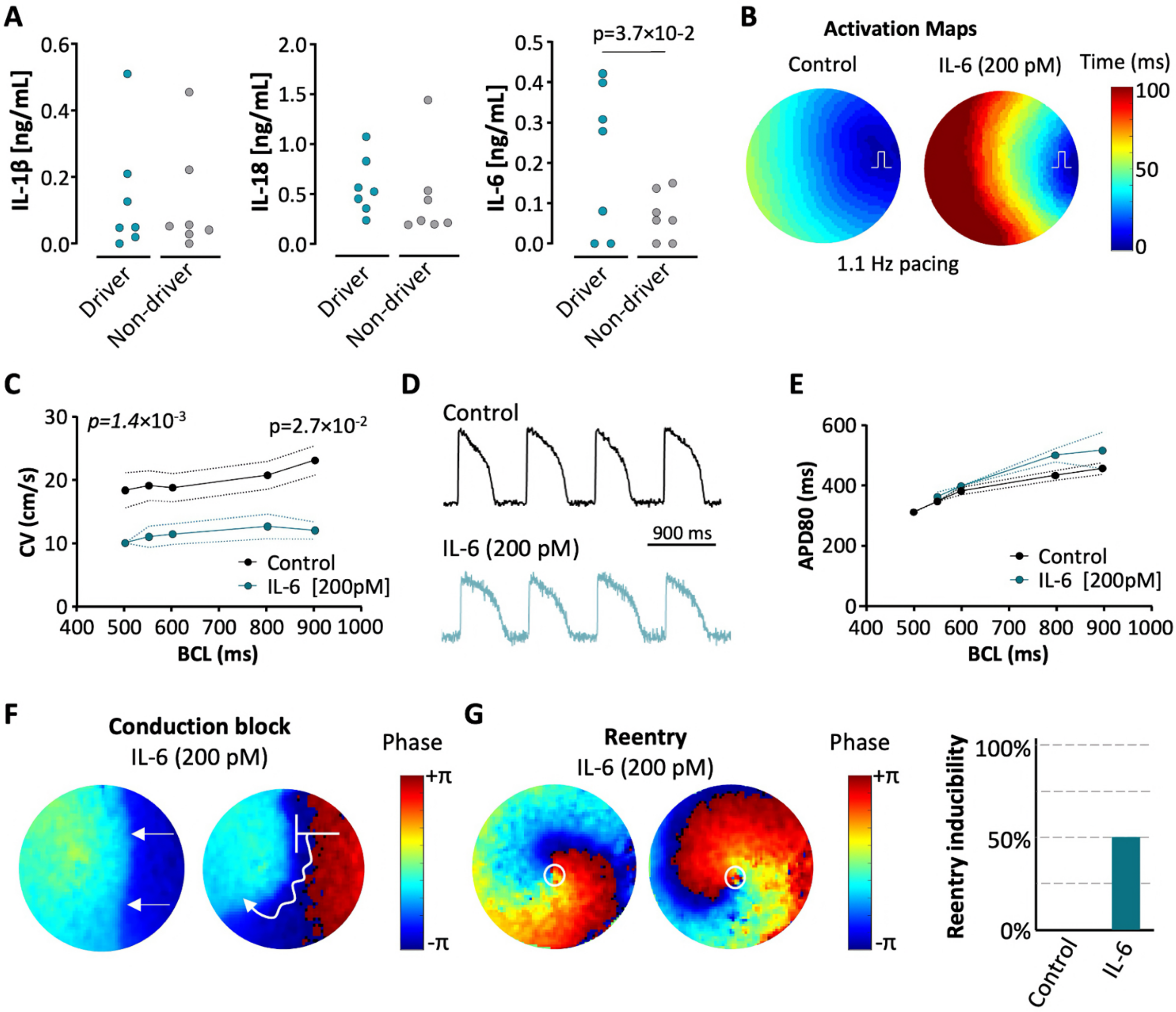

Figure S14. Electrophysiological effects in human induced pluripotent stem cell-derived cardiomyocytes of the differentially expressed interleukin-6 between driver and non-driver regions. **A**, Quantification of interleukin-1β (IL-1β), interleukin-18 (IL-18) and interleukin-6 (IL-6) protein expressions in driver *vs.* non-driver atrial homogenates from animals with long-lasting lone persistent atrial fibrillation (LL PsAF, N=7). A paired t-test was performed to assess statistical significance. **B**, Sample activation maps at 1.1 Hz of pacing cycle length in human induced pluripotent stem cell-derived cardiomyocyte (hiPSC-CM) monolayers treated with IL-6 solvent for 6 hours (control, left) and after incubation with IL-6 (200 pM) for the same time (right). IL-6 concentration was selected based on the average value detected in driver region samples of 7 animals with LL PsAF (IL-6: 212±182 pM). Colors represent sequential activation times with the earliest activation in blue. The pacing site is represented with a white square shape. **C**, Quantification and comparisons of conduction velocity (CV) values in hiPSC-CM monolayers treated with IL-6 and controls. **D**, Sample transmembrane voltage traces in optically mapped hiPSC-CM monolayers from the control (upper tracing, in black) and IL-6 groups (bottom tracing, in light blue). **E**, Quantification of action potential duration at 80% repolarization (APD80) in hiPSC-CM monolayers treated with IL-6 and controls. In **(C)** and **(E)**, the two-way analysis of variance (ANOVA) was used to assess statistical significance between IL-6 treated monolayers (n=6) and controls (n=7). Dashed lines in **(C)** and **(E)** indicate standard error of the mean values at different pacing cycle lengths for IL-6 treated monolayers (light blue dashed lines) and controls (black dashed lines). **F**, Sample pacing with unidirectional block and reentry formation in a hiPSC-CM monolayer treated with IL-6 (200 pM). **G**, Left, two snapshots of phase maps showing reentry in a hiPSC-CM monolayer treated with IL-6 (200 pM). White

circles indicate the singularity point of the reentrant pattern. Right, quantification of reentry inducibility in hiPSC-CM monolayers treated with IL-6 (200 pM) and controls. BCL: basic drive cycle length.

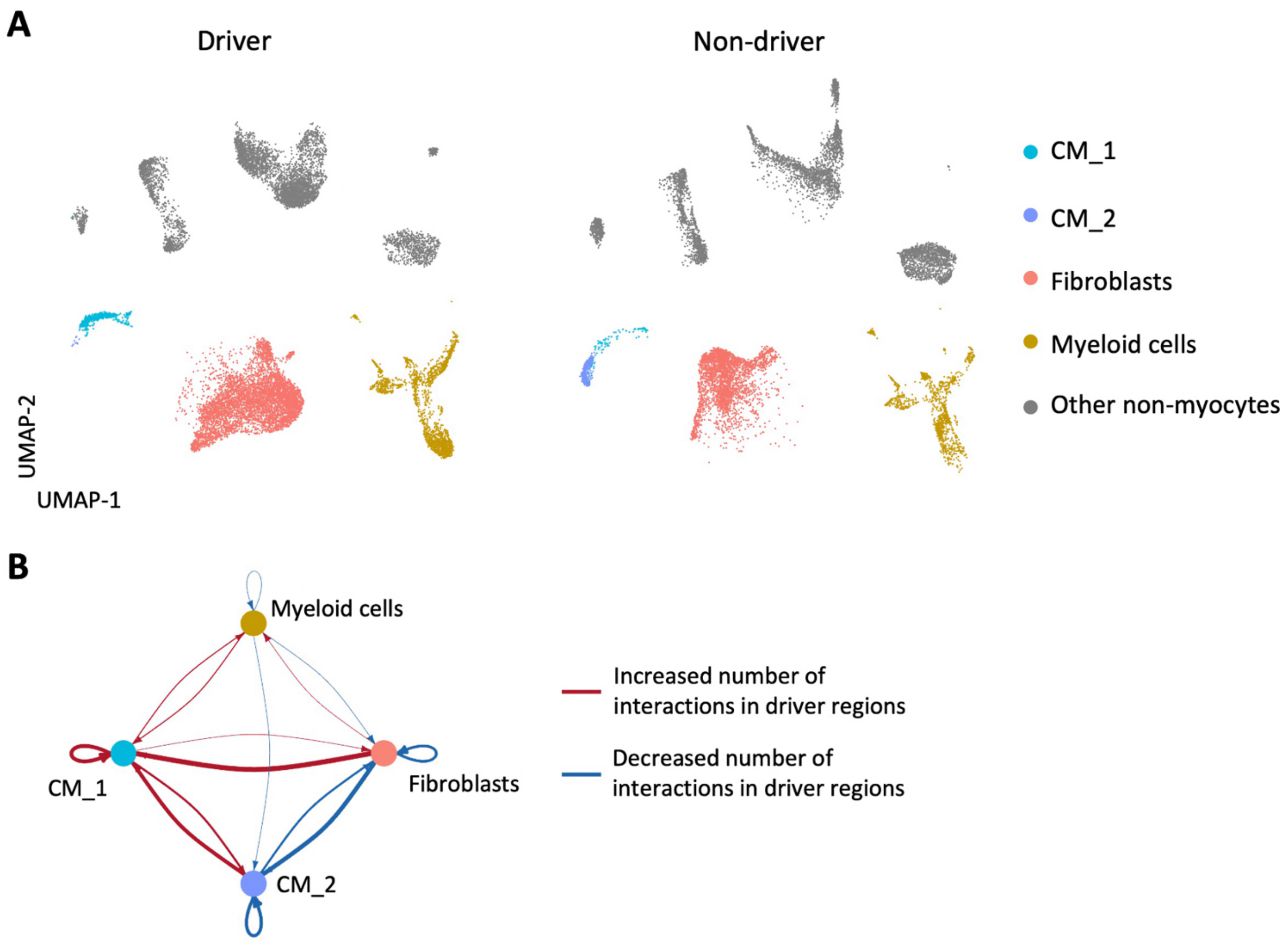

Figure S15. Cell-to-cell communication in atrial driver and non-driver regions of a pig with long-lasting lone persistent atrial fibrillation. A, Unsupervised Uniform Manifold Approximation and Projection (UMAP) clustering of an integrated dataset combining single-nuclei RNA-sequencing and single-cell RNA sequencing data from driver and non-driver regions of a pig with long-lasting lone persistent atrial fibrillation. **B**, Communication probability graph showing the differential number of interactions between driver and non-driver regions among cardiomyocyte (CM) clusters, fibroblasts and myeloid cells. The color-coding indicates a higher (red) or a lower (blue) signaling activity in driver relative to non-driver regions. Edge width is proportional to the total number of contributing ligand-receptor pairs between two interacting populations.

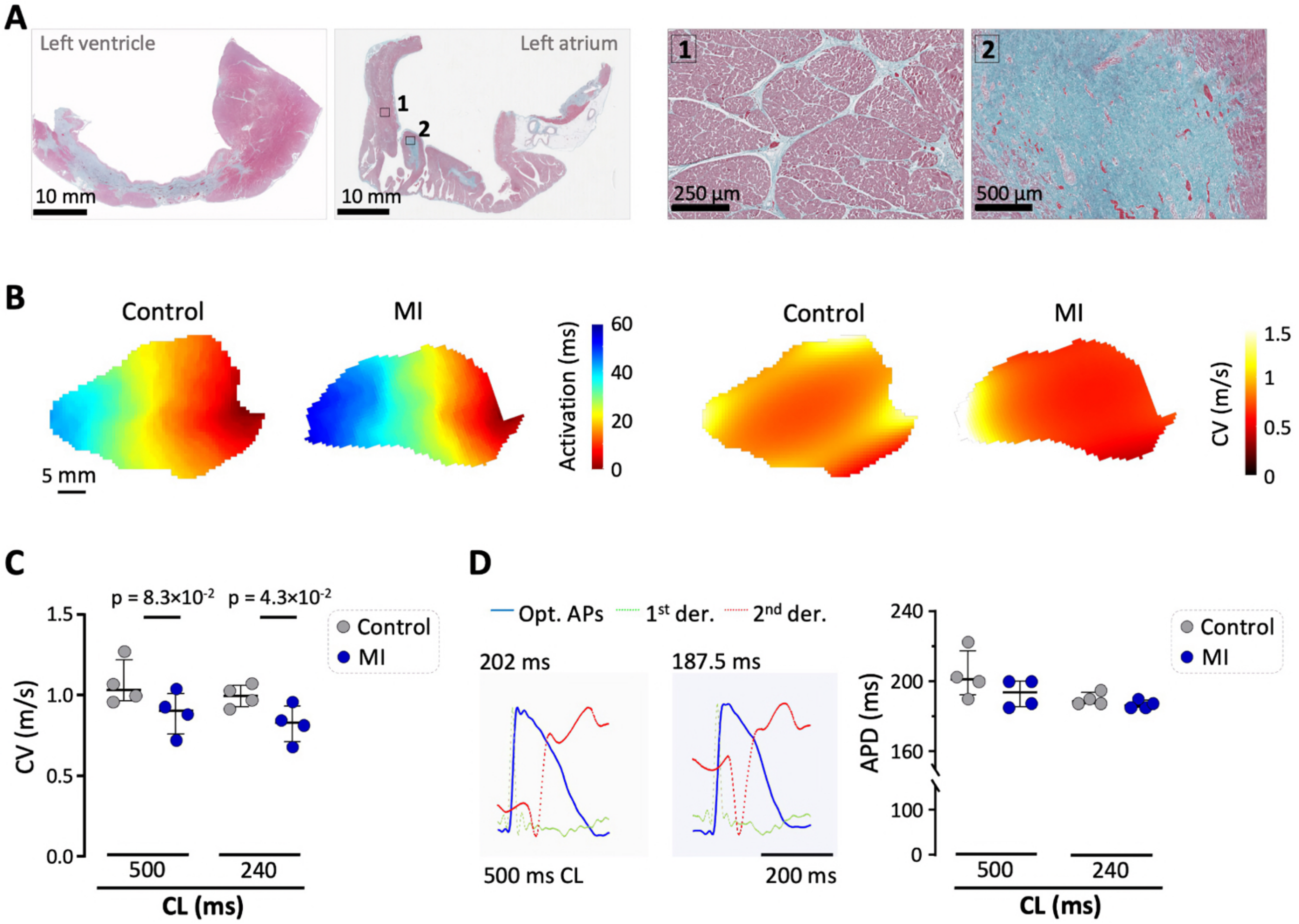

Figure S16. Fibrotic substrate and electrophysiological parameters in the left atrium of pigs with infarct-related substrate and healthy animals in the absence of atrial fibrillation remodeling. A, Sample Masson's trichrome stained sections from the left ventricle and left atrium of an animal with established myocardial infarction (MI). The samples were obtained 2-months after ischemia (3 hours)-reperfusion in the proximal circumflex artery. The infarct substrate generated both interstitial and compact fibrosis in the left atrium, in the absence of any remodelling associated with atrial fibrillation (AF). The samples were taken from one of the animals that died suddenly (see Figure S1) 2 months after MI before the beginning of the AF protocol. **B**, Representative activation (left) and conduction velocity (CV) maps (right) from *ex vivo* optical maps of transmembrane voltage changes in Langendorff perfused hearts of healthy animals and pigs with established infarct-related substrate (4.4 months [4.2, 4.4 months] after MI). This group of animals did not initiate the AF induction protocol, aiming to describe baseline electrophysiological differences between the left atrium of infarcted animals and healthy controls. **C**, Quantification and comparison of CV values in the left atrium of healthy pigs (N=4) and animals with infarct-related substrate (N=4) during pacing at 500 and 240 ms cycle length (CL). **D**, Left, sample quantifications of the action potential duration (APD) at 500 and 200 ms pacing CL. The APD was measured using the methodology described by Efimov et al. (Efimov *et al. Circulation 1994;90:1469-1480*) as the interval between the maximum first derivative (activation time, green dotted line) and the maximum second derivative (repolarization time, red dotted line). Comparison of optical APD values in the left atrium of healthy pigs (N=4) and animals with infarct-related substrate (n=4) at 500 and 240 ms of pacing CL. In (**C**) and (**D**), the two-way analysis of variance (ANOVA) was used to assess statistical significance of differences between means for animals with healthy atria and pigs with infarct-related substrate.

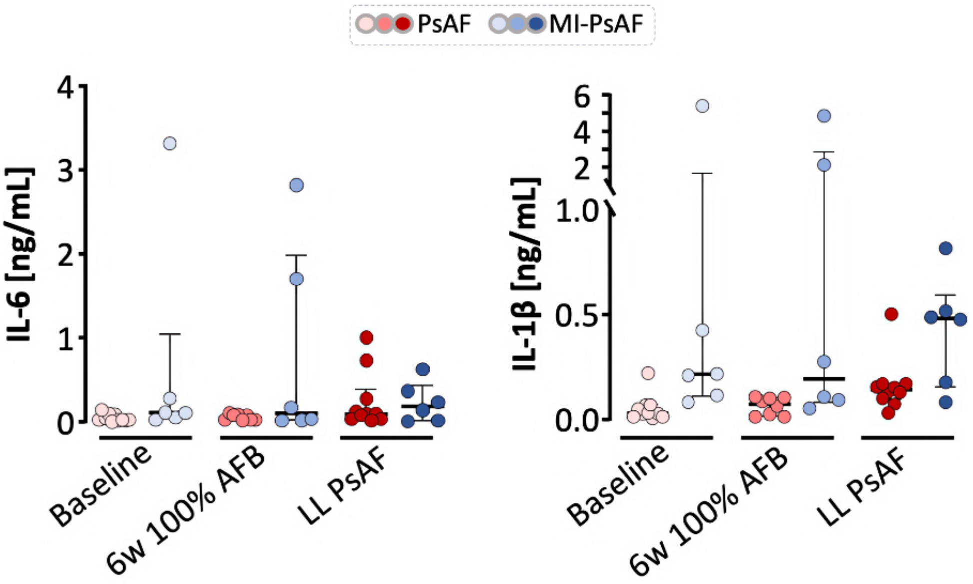

Figure S17. Changes in inflammatory biomarkers during atrial fibrillation progression in pigs with infarct-related substrate. Quantification of interleukin-6 (IL-6) and interleukin-1β (IL-1β) concentrations in plasma samples from the coronary sinus of pigs with lone persistent AF (PsAF) and pigs with PsAF and underlying infarct-related substrate (MI-PsAF). Quantifications and comparisons were performed at different time points of the follow-up (Baseline, after 6 weeks of 100% AF burden [6w 100% AFB] and at the of the follow-up during long-lasting PsAF. The two-way analysis of variance (ANOVA) was used for comparisons, followed by Šídák’s test (unpaired groups) for multiple comparisons.

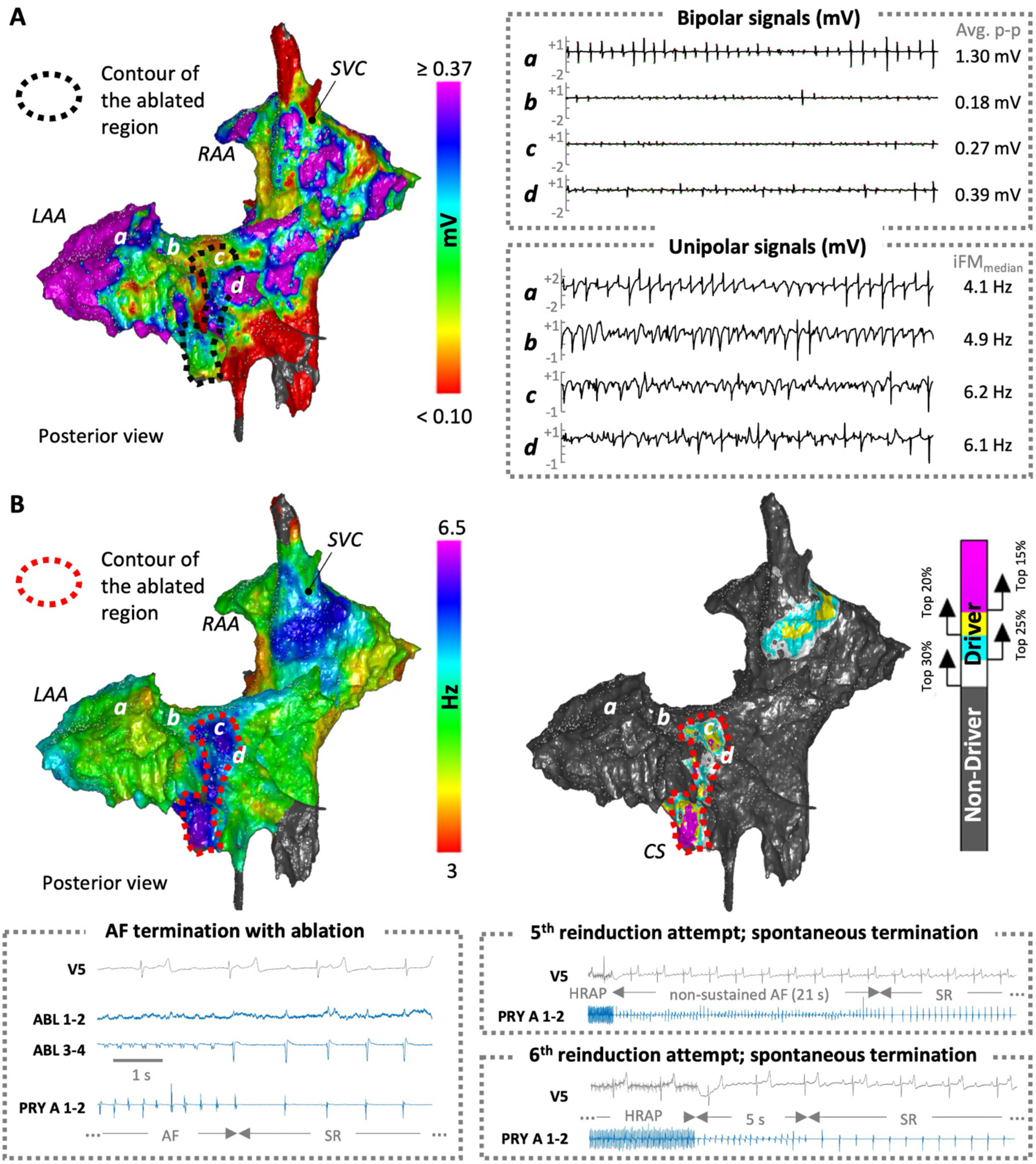

Figure S18. Atrial maps and ablation outcome in a sample pig with long-lasting persistent atrial fibrillation and underlying infarct-related substrate. **A**, Left, bipolar voltage map during atrial fibrillation (AF) showing the distribution of voltage-derived scar regions (<0.10 mV), healthy regions (≥0.37 mV) and heterogenous regions (between 0.10 and 0.37 mV). Atrial regions with normal voltage values are displayed in magenta. The remaining colors represent the transition to the lowest voltage regions, which are compatible with a higher degree of underlying scar. Right, sample bipolar and unipolar tracings from the locations indicated on the maps (a, b, c, d). The voltage map was generated with bipolar signals. Unipolar signals were used for instantaneous frequency modulation (iFM) analysis for driver identification (see Supplementary Materials for details). **B**, Top left, median instantaneous frequency modulation (iFM) map, in which regions with higher median iFM values (driver regions) are displayed in dark blue and magenta colors. Top right**,** driver map in which atrial regions activating faster than their surroundings and within the top 30% median iFM values (drivers) are color-coded in magenta, yellow, cyan and white, which correspond to the top 15%, top 20%, top

25% and top 30% of median iFM values. Conversely, regions with lower median iFM values (from 0-70% of median iFM values; i.e. non-driver regions) were colored in dark grey. Bottom left, persistent AF terminated after 16.9 minutes of radiofrequency energy delivery at the posterior left atrium and distal coronary sinus (CS). The ablated region is indicated with a dotted red contour on both the median iFM map and the driver map. Bottom right, sample surface and intracardiac tracings showing non-sustained AF episodes and spontaneous termination after reinduction attempts with burst pacing (6 reinduction attempts were performed, the last 2 are shown). Sample signals from ‘a’, ‘b’, ‘c’ and ‘d’ show normal bipolar voltage in a non-driver region, low voltage in a non-driver region, low voltage in a driver region and normal voltage in a driver region, respectively. HRAP: high-rate atrial pacing; RAA: right atrial appendage; SR: sinus rhythm; SVC: superior vena cava. ABL indicates signal recordings from bipoles of the ablation catheter. PRY A indicates signal recordings from bipoles 1-2 of the spline A of the multipolar mapping catheter.

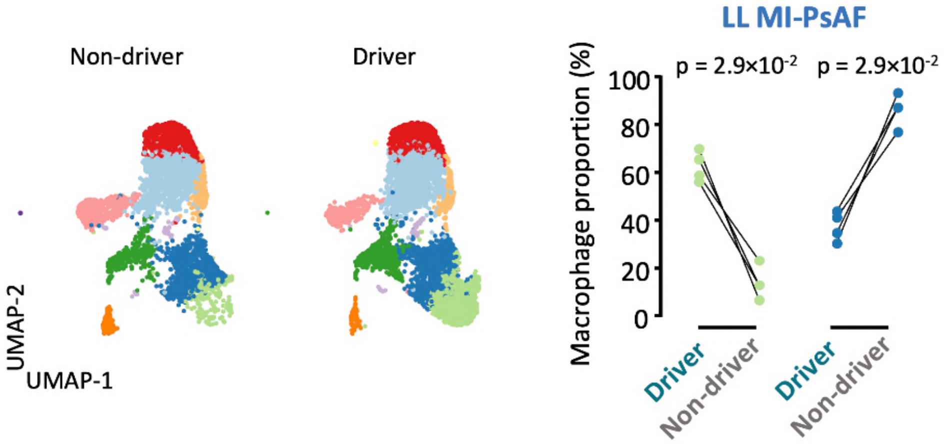

Figure S19. Tissue resident macrophages in driver and non-driver regions in pigs with persistent atrial fibrillation and underlying infarct-related substrate. Left, Uniform Manifold Approximation and Projection (UMAP) plots of myeloid clusters separated by anatomical areas (driver and non-driver regions) of animals with long-lasting lone persistent atrial fibrillation with infarct-related substrate (LL MI-PsAF). Right, quantification of Mac1 and Mac2 cluster proportion relative to total macrophages showing that Mac2 cluster, expressing a protective tissue resident signature, was more predominant in driver regions of animals with LL MI-PsAF. The opposite was true for Mac1 cluster. The Wilcoxon signed-rank test was used to assess statistically significant differences.

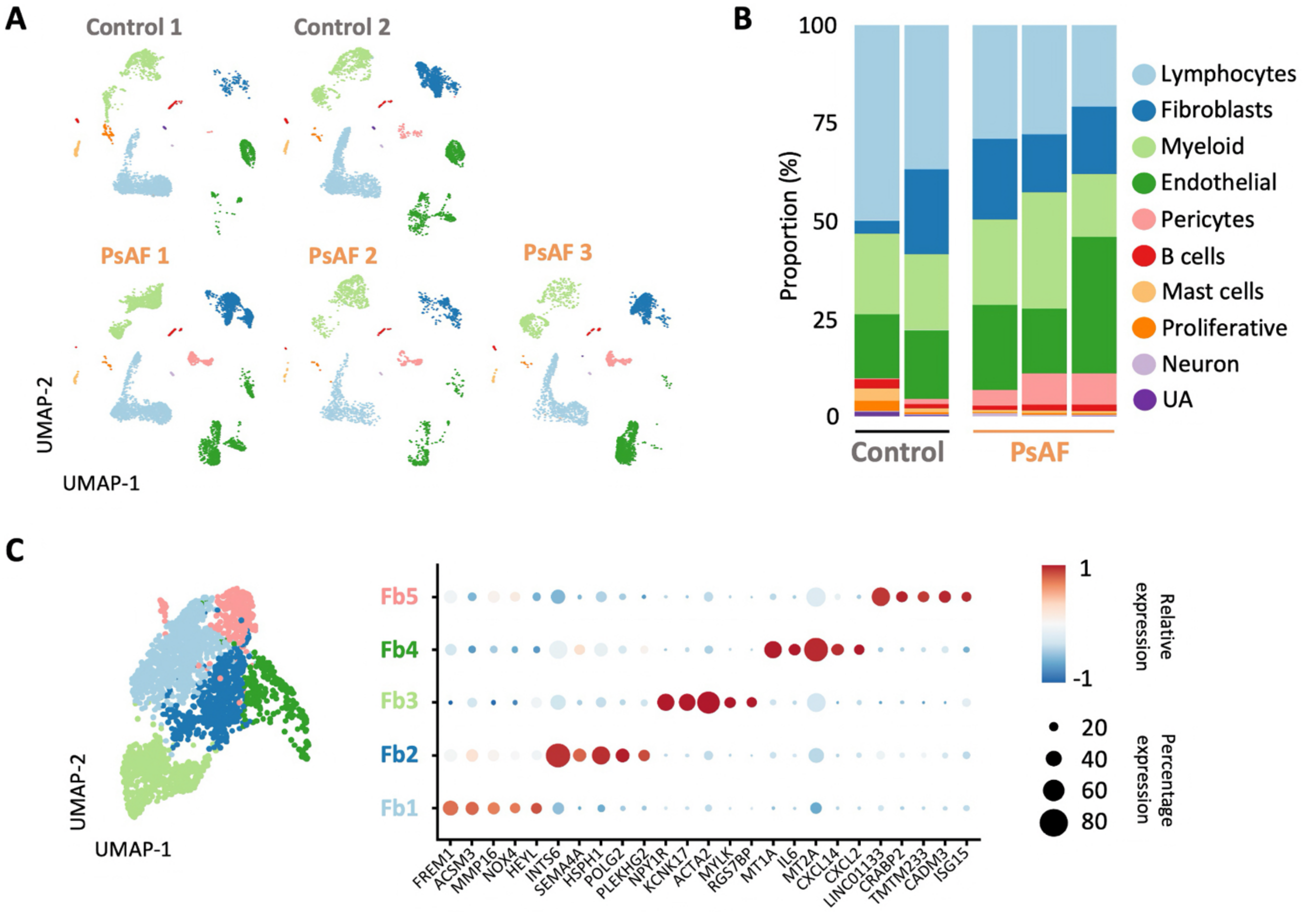

Figure S20. Cellular changes in non-myocyte cell populations of patients with persistent atrial fibrillation and controls in sinus rhythm. **A**, Uniform Manifold Approximation and Projection (UMAP) plots in individual single-cell RNA sequencing experiments from left atrial appendage tissue of patients with persistent atrial fibrillation (PsAF) (n=3) and controls (n=2). Distinct non-myocyte populations are color-coded. **B**, Examination of cell proportion reveals several clusters with changes in relative proportion in PsAF patients compared to controls. **C**, Left, high resolution UMAP plots identifies 5 cardiac fibroblast cluster in PsAF patients. Right, seurat-generated dot plot showing the five most upregulated genes per cluster. UA: Unannotated.

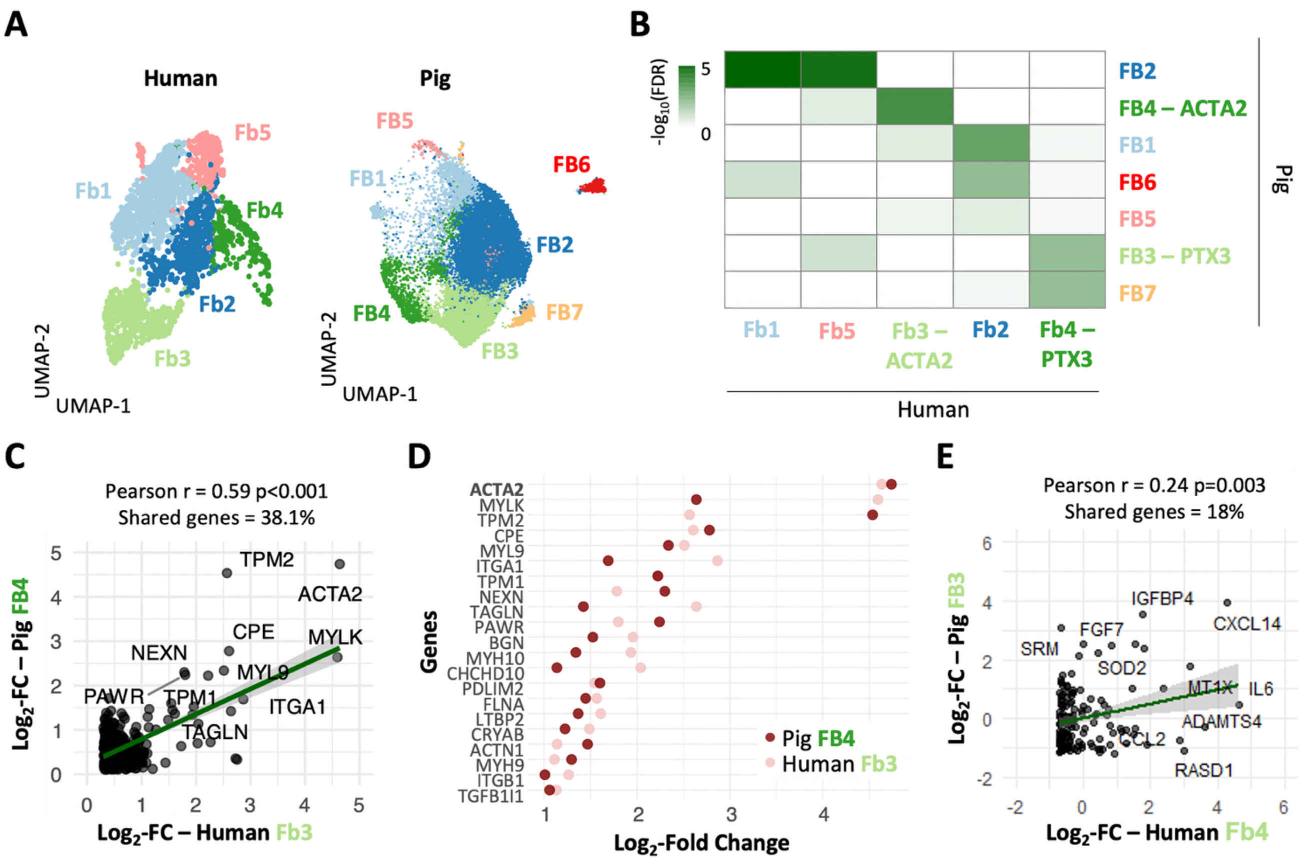

Figure S21. Comparison of fibroblasts clusters in driver regions of pigs with long-lasting lone persistent atrial fibrillation and humans. **A**, High-resolution unsupervised clustering of fibroblast clusters from driver regions of humans with persistent atrial fibrillation (PsAF) and pigs with long-lasting lone PsAF. Atrial tissue samples of driver regions in humans with PsAF were taken from the left atrial appendage (LAA) at the time of LAA closure during thoracoscopic-guided ablation. **B**, Heatmap comparing human and pig fibroblast clusters using their top 100 differentially expressed genes (DEG) compared to all other fibroblast clusters. Only genes expressed in both species were included. The significance of the gene overlap between human and pig clusters was obtained by two-sided Fisher's exact test with multiple testing corrected by the Benjamini–Hochberg method. The color represents the $-\log_{10}$ false discovery rate (FDR) (green: high; white: low). **C**, Comparison of DEG between human and pig fibroblast clusters expressing ACTA2. Each point represents a common gene to both clusters, with $\log_2$-fold change (FC) values for human (x-axis) and pig (y-axis). The green line shows the fitted linear regression with confidence intervals. The most relevant genes, based on the $\log_2$-FC values, are labeled. **D**, List of genes expressed in both species and their respective $\log_2$-FC. **E**, Same as in **(C)** for human and pig fibroblast clusters expressing PTX3.

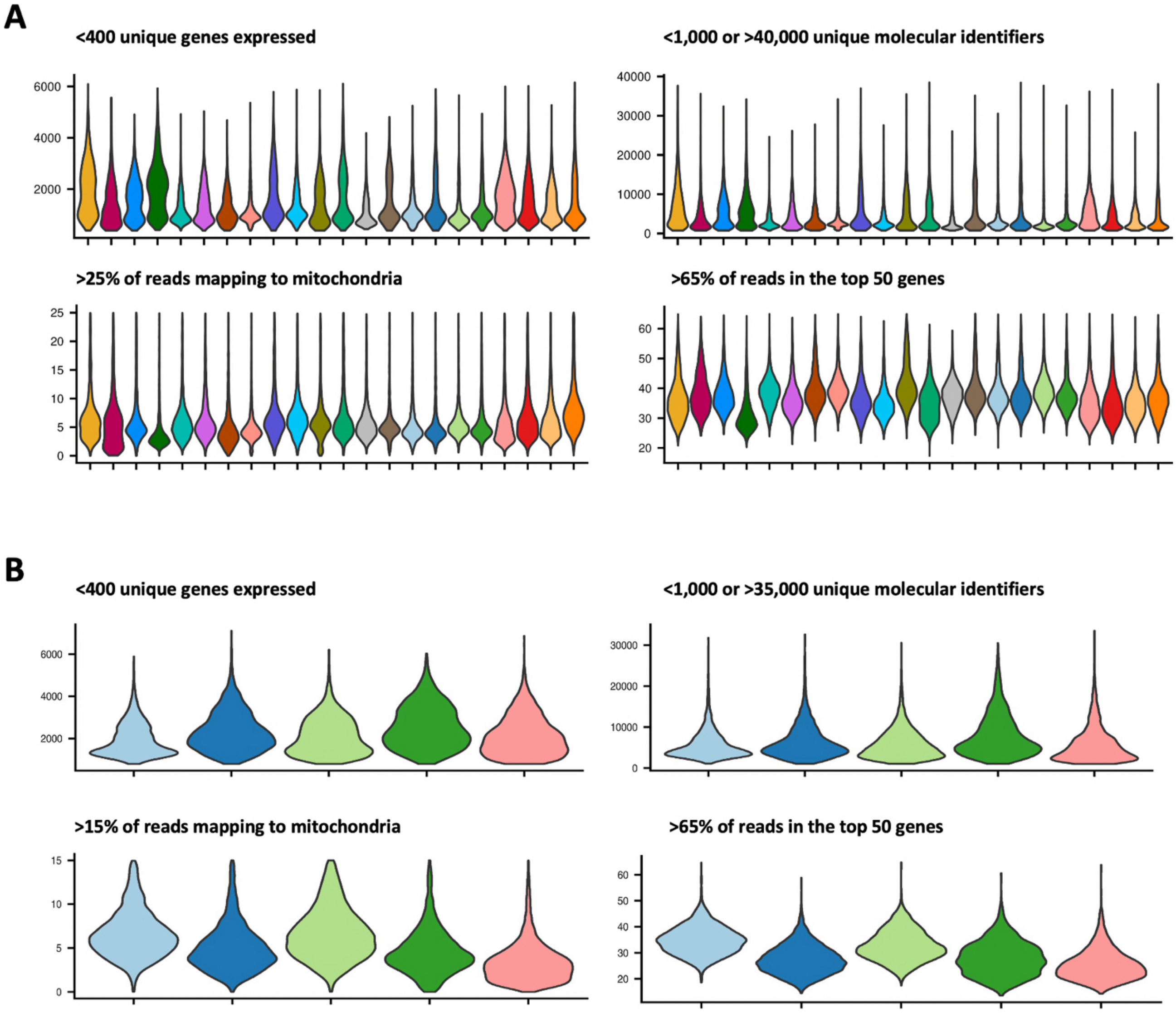

Figure S22. Quality control and cell filtering for single-cell RNA sequencing samples. A, For porcine samples, cells meeting any of the following criteria were filtered out: <400 unique genes expressed, <1,000 or >40,000 unique molecular identifiers, >25% of reads mapping to mitochondria, >65% of reads in the top 50 genes. **B**, For human samples, cells meeting any of the following criteria were filtered out: <400 unique genes expressed, <1,000 or >35,000 unique molecular identifiers, >15% of reads mapping to mitochondria, >65% of reads in the top 50 genes.

SUPPLEMENTARY TABLES

Table S1. Animal characteristics in pigs with long-lasting lone persistent atrial fibrillation (used for *ex vivo* analysis after *in vivo* electroanatomical mapping) and in sham-operated controls.

	PsAF (N=13)	Sham-operated (N=9)	p-value
**Age at the beginning of the study (months)**	6.5 (6.1, 7.2)	7.6 (7.0, 7.7)	0.075
**Weight at the beginning of the HRAP (kg)**	51.5 (50.0, 55.0)	54.0 (50.0, 66.5)	0.055
**Male / Female (% / %)**	8 / 5 (61.5 / 38.5)	3 / 6 (37.5 / 62.5)	0.193
**Echocardiography:**			
**LVEF (%)**	67.5 (63.5, 71.6)	69.6 (62.6, 72.6)	0.866
**LVPWd (cm)**	0.86 (0.81, 0.90)	0.93 (0.87, 1.02)	0.123
**IVSd (cm)**	1.01 (0.96, 1.05)	1.03 (1.00, 1.11)	0.103
**Time to PsAF (months)**	5.8 (3.8, 8.6)	N/A	N/A
**Total study time (months)**	15.3 (13.9, 17.1)	15.4 (14.3, 15.8)	0.832
**Weight at end of the protocol (kg)**	109.0 (91.5, 122.5)	116.0 (112.0, 127.0)	0.243

For continuous variables, the unpaired two-sided Student's t test was used to assess differences, after testing for normality with a Shapiro–Wilk's test. Otherwise, the Wilcoxon's rank sum test was used. For categorical variables, the Pearson's chi–squared test was used to assess differences when the expected frequencies were higher than 5. HRAP: high-rate atrial pacing; IVSd: interventricular septum thickness in diastole; LVEF: left ventricular ejection fraction; LVPWd: left ventricular posterior wall thickness in diastole; N/A: not aplicable; PsAF: persistent atrial fibrillation. Data are presented as median and interquartile range.

Table S2. Animal characteristics in pigs with lone persistent atrial fibrillation that underwent catheter-based ablation at driver and non-driver regions.

	PsAF – ablation of driver regions (N=8)	PsAF – ablation of non-driver regions (N=4)	p-value
**Age at the beginning of the study (months)**	5.9 (5.6, 7.4)	9.0 (8.5, 9.4)	**0.047**
**Weight at the beginning of the HRAP (kg)**	45.0 (43.4, 54.0)	58.3 (57.3, 59.0)	**0.002**
**Male / Female (% / %)**	7 / 1 (87.5 / 12.5)	0 / 4 (0 / 100)	**0.010**
**Echocardiography:**			
**LVEF (%)**	68.0 (63.5, 71.6)	70.1 (64.6, 72.6)	0.832
**LVPWd (cm)**	0.90 (0.86, 0.98)	0.90 (0.77, 0.94)	0.250
**IVSd (cm)**	1.00 (0.92, 1.04)	0.97 (0.98, 1.02)	0.500
**Time to PsAF (months)**	4.3 (2.6, 8.1)	2.8 (2.6, 3.5)	0.267
**Total study time (months)**	10.0 (8.4, 10.8)	8.7 (8.2, 9.3)	0.449
**Weight at end of the protocol (kg)**	103.1 (93.5, 111.0)	94.6 (87.8, 98.1)	0.447

For continuous variables, the unpaired two-sided Student's t test was used to assess differences, after testing for normality with a Shapiro–Wilk's test. Otherwise, the Wilcoxon's rank sum test was used. For categorical variables, the Fisher's exact test was used. HRAP: high-rate atrial pacing; IVSd: interventricular septum thickness in diastole; LVEF: left ventricular ejection fraction; LVPWd: left ventricular posterior wall thickness in diastole; PsAF: persistent atrial fibrillation. Data are presented as median and interquartile range.

Table S3. Animal characteristics of pigs with persistent atrial fibrillation and underlying infarct-related atrial substrate

	MI-PsAF (N=15)
**Age at the time of myocardial infarction (months)**	5.8 (5.0, 6.1)
**Age at the beginning of the study (months)**	8.1 (7.7, 9.2)
**Weight at the time of myocardial infarction (kg)**	44.5 (41.5, 48.0)
**Weight at the beginning of the HRAP (kg)**	60.8 (57.0, 70.5)
**Male / Female (% / %)**	7 / 8 (42.9 / 57.1)
**LVEF (%)**	55.1 (50.3, 58.8)
**Time to PsAF (months)**	5.2 (4.3, 5.6)
**Total study time (months)**	13.3 (12.7, 14.4)
**Weight at end of the protocol (kg)**	109.0 (100.3, 114.5)

AF: atrial fibrillation; HRAP: high-rate atrial pacing; LVEF: left ventricular ejection fraction; MI: myocardial infarction; PsAF: persistent atrial fibrillation. Data are presented as median and interquartile range.

Table S4. Animal characteristics in pigs with infarct-related substrate and in controls with healthy atria used for *ex vivo* optical mapping in whole heart preparations.

	Myocardial infarction (N=4)	Control (N=4)
**Age at the time of MI (months)**	5.8 (5.0, 6.1)	–
**Weight at the time of MI (kg)**	61.5 (55.8, 69.8)	–
**Male / Female (% / %)**	4 / 0 (100 / 0)	3 / 1 (75 / 25)
**LVEF (%)**	54.5 (50.9, 58.5)	69.4 (66.9, 72.0)
**Weight at the time of euthanasia (kg)**	96.3 (82.8, 114.0)	129.0 (119.3, 140.6)

LVEF: left ventricular ejection fraction.. MI: myocardial infarction. Data are presented as median and interquartile range.

Table S5. Proteins with statistically significant changes in the tissue proteome of driver regions compared to non-driver regions in pigs with persistent atrial fibrillation.

Categories	Genes
**Protection over pressure overload**	CMYA5 / MYDGF
**Anti-apoptosis**	BAG3
**Pro-apoptosis**	TMEM109 / APP / TPP2 / DKK3
**Others**	FABP4 / ACLY / ABHD10 / PLIN4 / MPZ / PLIN1 / CA3 / FASN / MYH11 / AK4 / CALU / tr|A0A287BL83|A0A287BL83_PIG Carbonyl reductase (NADPH) OS=Sus scrofa OX=9823 PE=1 SV=1 / PPM1G / MYH6 / DDAH1 / COL1A2 / FABP7 / SUOX / CALD1 / MYLK / COL1A1 / MBP / SNTB1 / TES / SPARCL1 / TAGLN / DCN / ABI3BP / CAVIN3 / ACE / ASPN / TPM2 / PLEKHA2 / NENF / THBS4 / ME1 / OGN / GLRX / CNN1 / EPHX1 / ALDOC / COL3A1 / ATP1A3 / sp|Q29550|EST1_PIG Liver carboxylesterase OS=Sus scrofa OX=9823 PE=1 SV=1 / TNC / AK1 / HHATL / GNAI1 / PRKACA / COL6A5 / LDB3 / PPP1R2 / CDC42BPB / tr|A0A287AD92|A0A287AD92_PIG GrpE protein homolog OS=Sus scrofa OX=9823 PE=1 SV=2 / PTER / UBAP2L / DHRS7 / ADCY3 / EPB41L3 / MYH4 / EPRS1 / GSK3A / CCDC141 / IPO5 / ITM2B / RPS8 / NUDCD3 / GPCPD1 / PCP4L1 / NDUFAF5 / COPB1 / CKB / SORBS2 / TSTD3 / RPL23A / CCT4 / LAMB1 / USP14 / ARF1 / SLC25A20 / PDLIM1 / ISCA2 / MYH14 / CCT7 / DDX1 / DYSF / PTBP1 / TMED7 / CLIC5 / RPL24 / VWA8 / CCT6A / LOC100624149 / FTH1 / RPL35A / LOC100516390 / XDH / RPL17 / EPB41L3 / FECH / FAM120C / RTRAF / MYPN / tr|A0A5G2QZZ3|A0A5G2QZZ3_PIG LIM zinc-binding domain-containing protein OS=Sus scrofa OX=9823 PE=4 SV=2 / REEP5 / MYL1 / NPEPPS / RPSA / DUSP3 / RPL23 / CCT8 / ECHDC3 / XPO1 / tr|A0A287AYR8|A0A287AYR8_PIG LIM zinc-binding domain-containing protein OS=Sus scrofa OX=9823 PE=4 SV=1 / FLOT2 / SPTBN1 / C9H11orf1 / TMEM43 / PALMD / ANXA5 / NCEH1 / SPTAN1 / HADHB / tr|A0A287APR1|A0A287APR1_PIG 60S ribosomal protein L18a OS=Sus scrofa OX=9823 PE=1 SV=1 / PYGB / TCP1 / CCT5 / PSME2 / TSTD1 / HADHA / RTCB / CLTC / NLN / PRKAR1A / HPRT1 / GCN1 / USO1 / CRIP2 / NME2 / CAMKK2 / RPL14 / MICAL1 / APOOL / GRB2 / HSPB3 / NES / PYGL / LAP3 / DSG2 / LETM1 / CAVIN2 / ARMCX3 / LDHB

Only proteins quantified with two or more peptides are shown. Proteins without an annotated gene are displayed with the FASTA protein description.

Table S6. Single cell RNA-sequencing cell counts per sample and atrial region across experimental groups (sham-operated, long-lasting persistent atrial fibrillation, and persistent atrial fibrillation with underlying infarct-related substrate).

Sample	Driver (CS and its adjacent PLA)	Extra_driver (SCV)	Non-driver (RA free wall)	Total	Total per model
Sham-Op_1	6,733	NA	5,671	12,404	
Sham-Op_2	2,082	NA	6,211	8,293	
Sham-Op_3	5,368	NA	6,301	11,669	32,366
PsAF_1	4,862	NA	3,885	8,747	
PsAF_2	5,806	NA	3,923	9,729	
PsAF_3	4,839	*Post-ablation*	4,491	9,330	
PsAF_4	7,039	5,386	3,475	15,900	43,706
MI-PsAF_1	6,468	NA	7,798	14,266	
MI-PsAF_2	8,151	NA	6,436	14,587	
MI-PsAF_3	4,423	6,396	4,775	15,594	44,447
				Total #cells	120,519

CS: coronary sinus; MI: myocardial infarction; NA: not available; PLA: posterior left atrium; PsAF: persistent atrial fibrillation; RA: right atrium; SCV: superior cava vein. Extra-driver indicates a second top driver region with the highest median instantaneous frequency modulation values.

Table S7. Baseline characteristics and after 2 years of follow-up in patients from the pilot series with persistent atrial fibrillation undergoing invasive mapping and ablation of driver regions.

Study variables	Baseline	2-year follow-up	*p-value*
**Age (years)**	57.8 ± 9.1	59.8 ± 9.1	
**Sex female/male (*n* (%))**	2/8 (20/80)	2/8 (20/80)	—
**Clinical history**			
Body mass index (kg/m^2)	25.3 ± 3.3	25.1 ± 3.4	0.910
Lean body mass (kg)	75.4 ± 13.3	75.8 ± 14	0.950
Hypertension (*n* (%))	4 (40)	4 (40)	>0.999
Dyslipidemia (*n* (%))	3 (30)	6 (60)	>0.999
Diabetes mellitus (*n* (%))	2 (20)	2 (20)	>0.999
Stroke or TIA (*n* (%))	0 (0)	1 (10)	>0.999
CHA_2DS_2-VASc	1.0 ± 1.0	1.0 ± 1.0	>0.999
**Medication (*n* (%))**			
β-blockers (*n* (%))	9 (90)	7 (70)	0.582
ACEis/ARBs/MRAs (*n* (%))	4 (40)	3 (30)	>0.999
Statins (*n* (%))	2 (20)	5 (50)	0.350
Oral anticoagulation (*n* (%))	10 (100)	5 (50)	**0.033**
Diuretics (*n* (%))	1 (10)	2 (20)	>0.999
Digoxin (*n* (%))	1 (10)	0 (0)	>0.999
Calcium channel blockers (*n* (%))	2 (20)	2 (20)	>0.999
Antiarrhythmic drugs			
Class IC (*n* (%))	5 (50)	2 (20)	0.387
Class III agents (*n* (%))	5 (50)	1 (10)	**0.012**
**Echocardiography parameters**			
Left ventricular ejection fraction (%)	59.7 ± 5.4	63.1 ± 3.9	0.152
LA area indexed (cm²/m²)	10.8 ± 2.4	10.1 ± 2.2	0.416
**AF type**			
Persistent AF (*n* (%))	10 (100)	1 (10)	**<0.001**
AF history (years)	6.5 ± 4.9	—	—
AF episode duration before ablation (months)	7.0 ± 5.5	—	—
AF recurrence[#] (*n* (%))	—	1 (10)	—

Continuous variables are shown as mean ± standard deviation. Categorical variables are shown as n (%). [#]This patient with documented AF recurrence refused further thoracoscopic-guided ablation to target the remaining driver sites, which were not suitable for catheter-based ablation according to the ablation protocol (See Supplementary Materials for details). For continuous variables, the unpaired two-sided Student's t test was used to assess differences, after testing for normality with a Shapiro–Wilk's test. Otherwise, the Wilcoxon's rank sum test was used. For categorical variables, the Pearson's chi–squared test was used to assess differences when the expected frequencies were higher than 5. Otherwise, the Fisher's exact test was used. AF: atrial fibrillation; ACEis: angiotensin-converting enzyme inhibitors; ARBs: angiotensin II receptor blockers; LA: left atrium; MRA: mineralocorticoid receptor antagonists; TIA: transient ischemic attack. CHA_2DS_2-VASc scoring indicates: Congestive heart failure - 1, Hypertension - 1, Age (≥75 years) - 2, Diabetes mellitus - 1, prior Stroke or TIA or thromboembolism - 2, Vascular disease - 1, Age (65 to 74 years) - 1, and Sex category (female) - 1.

Table S8: Baseline characteristics of patients with available atrial samples at the time of thoracoscopic-guided ablation (atrial fibrillation patients) or open-chest cardiac surgery (sinus rhythm controls).

Study variables	Patients with PsAF (N = 10)	Patients in sinus rhythm (N = 7)	*p-value*
**Age (years)**	63.0 ± 7.0	55.9 ± 15.8	0.201
**Sex female/male (*n* (%))**	2/8 (20/80)	0/7 (0/100)	0.485
**Clinical history**			
Body mass index (kg/m2)	27.3 ± 2.4	27.1 ± 6.4	0.970
Lean body mass (kg)	82.3 ± 13.3	79.0 ± 24.6	0.431
Hypertension (*n* (%))	4 (40)	4 (57.1)	0.637
Dyslipidemia (*n* (%))	5 (50)	4 (57.1)	>0.999
Diabetes mellitus (*n* (%))	0 (0)	1 (14.3)	0.412
Previous stroke or TIA (*n* (%))	0 (0)	0 (0)	>0.999
CHA_2DS_2-VASc	1.0 ± 0.9	1.4 ± 1.3	>0.999
**Cardiopathy**			
Ischemic (*n* (%))	0 (0)	5 (71.4)	**0.003**
Ascending aortic aneurysm (*n* (%))	0 (0)	2 (14.3)	0.412
**Baseline medication (*n* (%))**			
β-blockers (*n* (%))	6 (60)	4 (57.1)	>0.999
ACEis/ARBs/MRAs (*n* (%))	2 (20)	2 (28.6)	>0.999
Statins (*n* (%))	5 (50)	4 (57.1)	>0.999
Oral anticoagulation (*n* (%))	10 (100)	1 (14.3)	**<0.001**
Antiaggregant (*n* (%))	0 (0)	4 (57.1)	**0.015**
Digoxin (*n* (%))	1 (10)	0 (0)	>0.999
Calcium channel blockers (*n* (%))	3 (30)	2 (28.6)	>0.999
Antiarrhythmic drugs			
Class I C (*n* (%))	2 (20)	0 (0)	0.485
Class III agents (*n* (%))	0 (0)	0 (0)	>0.999
**Echocardiography parameters**			
Left ventricular ejection fraction (%)	60.5 ± 9.0	60.7 ± 6.4	0.844
LA area indexed (cm^2/m^2)	15.6 ± 4.5	10.5 ± 2.6	**0.003**
**AF type**			
Persistent AF (*n* (%))	10 (100)	0 (0)	**<0.001**
AF history (years)	10.9 ± 6.8	—	—
AF episode duration before ablation (months)†	6.0 (3.5, 12.8)	—	—

Continuous variables are shown as mean ± standard deviation unless otherwise indicated. (†) indicates data presented as median and interquartile range. Categorical variables are shown as n (%). For continuous variables, the unpaired two-sided Student's t test was used to assess differences, after testing for normality with a Shapiro–Wilk's test. Otherwise, the Wilcoxon's rank sum test was used. For categorical variables, the Pearson's chi–squared test was used to assess differences when the expected frequencies were higher than 5. Otherwise, the Fisher's exact test was used. AF: atrial fibrillation; ACEis: angiotensin-converting enzyme inhibitors; ARBs: angiotensin II receptor blockers; LA: left atrium; MRA: mineralocorticoid receptor antagonists; TIA: transient ischemic attack; CHA_2DS_2-VASc scoring indicates: Congestive heart failure - 1, Hypertension - 1, Age (≥75 years) - 2, Diabetes mellitus - 1, prior Stroke or TIA or thromboembolism - 2, Vascular disease - 1, Age (65 to 74 years) - 1, and Sex category (female) - 1.

Table S9. Single-cell RNA sequencing cell counts per sample in patients with symptomatic persistent atrial fibrillation and controls in sinus rhythm.

Sample	Total
Control_1	3,615
Control_2	4,875
PsAF_1	6,243
PsAF_2	1,872
PsAF_3	3,970
	20,575

PsAF: persistent atrial fibrillation

Unedited immunoblots

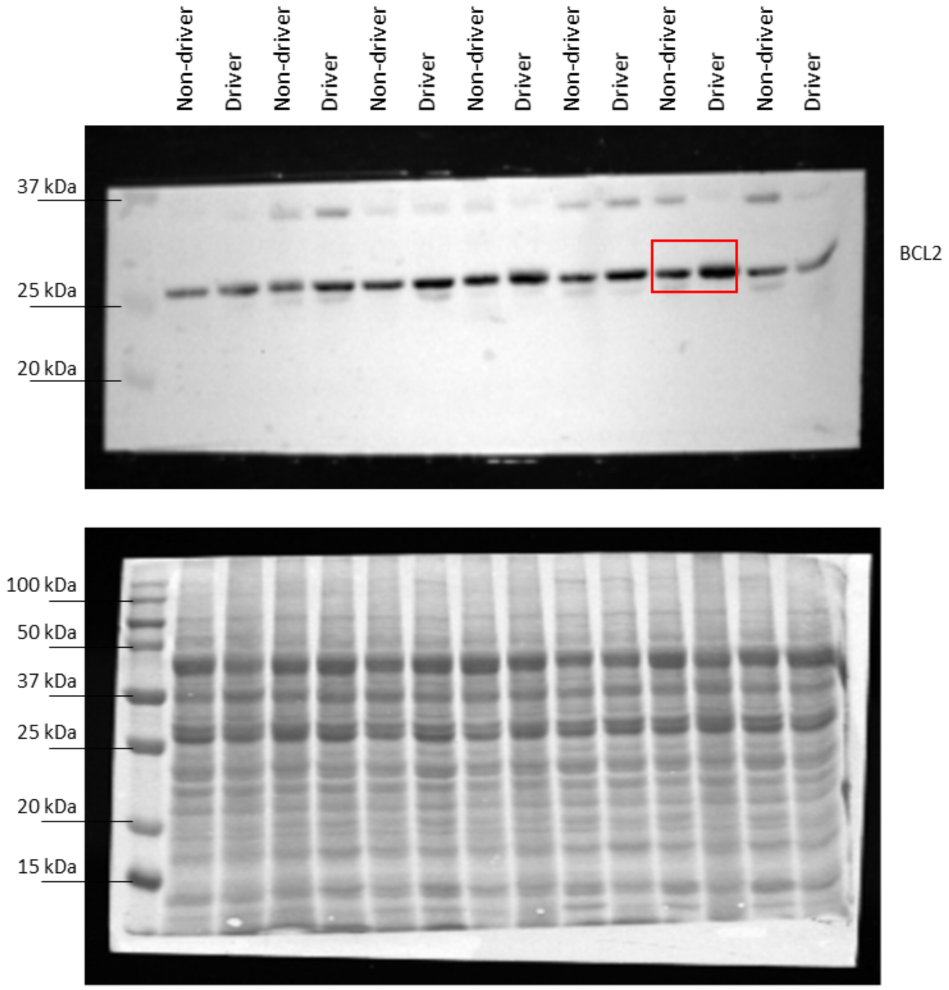

Unedited western blots reported in **Figure 4I**. Red square show the selected bands represented in the main Figure.

Major Resources Table

Animals (in vivo studies)

Species	Vendor or Source	Background Strain	Sex	Persistent ID / URL
Sus Scrofa	CNIC	Yucatan-Large White	Male and Female	

Antibodies (Sus Scrofa and Human)

Target antigen	Vendor or Source	Clone		Working dilution
Mouse-anti-porcine CD45	INIA	2A5	Dylight 405	1:1000
Mouse-anti-porcine CD172a	INIA	BA1C11	DyLight 594	1:500
Mouse-anti-porcine CD163	INIA	2A10	DyLight 488	1:1000
Mouse-anti-porcine CD169	INIA	1F1	DyLight 650	1:1000
Mouse-anti-porcine SLA-II	INIA	2E9/13	DyLight 680	1:1000
Mouse-anti-human CD45	Biologend	2D1	PerCP	0.2 µg/ml
Mouse-anti-human CD64	Biologend	10.1	BV421	0.1 µg/ml
Mouse-anti-human CD14	Biologend	M5E2	Fitc	0.8 µg/ml
Mouse-anti-human HLADR	Biologend	L243	APCCy7	0.2 µg/ml
Mouse-anti-human CCR2	Biologend	K036C2	APC	0.16 µg/ml

INIA: Instituto de Investigación y Tecnología Agraria y Alimentaria

Gene	Forward primer	Reverse primer
GAPDH	CCATCTTCCAGGAGCGAGAT	AGAAGGGGCAGAGATGATGA
IL-1β	GCCAGTCTTCATTGTTCAGGTTT	ATCTCTTTGGGGCCATCAGC
IL-6	TGGACGGCATCAATCTCA	GACCCTGAGGCAAAAGGGAA
TNF-α	GGCCCAAGGACTCAGATCAT	CTGTCCCTCGGCTTTGACAT

Primers (Sus Scrofa)

GAPDH: Glyceraldehyde-3-phosphate dehydrogenase; IL-1β: Interleukin 1 beta; IL-6: Interleukin 6; TNF-α: Tumor necrosis factor alpha.

Data & Code Availability

Description	Source / Repository	Identifier
Mass spectrometry proteomics raw data	ProteomeXchange	PXD053398
Single-cell RNA sequencing raw data	BioStudies	E-MTAB-14275

ARRIVE GUIDELINES

Study Design

Groups	Sex Male / Female	Age	Number (prior to experiment)	Number (after termination)	Littermates (Yes/No)
Healthy controls	3 / 1	22.5 (20.5, 25.0)	4	4	No
Sham-operated controls	3 / 6	7.6 (7.0, 7.7)	9	9	No
Persistent atrial fibrillation	15 / 10	6.5 (5.9, 8.3)	25	25	No
Persistent atrial fibrillation with infarct-related substrate	7 / 8	5.8 (5.0, 6.1)	15	15	No
Myocardial infarction	4 / 0	13.2 (11.5, 13.8)	4	4	No

Data are presented as median and interquartile range. Animals not included in the table: Four pigs with myocardial infarction died before the beginning of the atrial fibrillation protocol due to sudden cardiac death. Four more pigs with persistent atrial fibrillation and underlying infarct-related substrate died suddenly during the atrial fibrillation protocol. Two animals in the group of persistent atrial fibrillation died due to intercurrent pneumonia during the protocol.

Sample Size

The initial sample size was estimated based on the expected changes in atrial electrical remodeling during atrial fibrillation (AF) progression. The dominant frequency of bipolar atrial tracings was used to assess atrial activation rates and the changes on atrial electrical remodeling over the follow-up. The initial sample size was estimated to detect a relative 10% change in dominant frequency values with power of 90% and significance of 5%. Sample size estimations for comparisons between AF animals and sham-operated controls, between driver and non-driver regions in AF animals, and between potentially driver and non-driver regions in healthy controls, were estimated based on previous data on the literature supporting a minimum of 5 animals or samples to reach statistical significance.

Inclusion Criteria

We used slow growing crossbred pigs (Yucatan–Large White) to minimize problems arising from handling over-large animals after long periods with AF. The gold-standard model for translational research in cardiovascular disease is the pig, which we use extensively at CNIC due to its anatomical and physiological similarities to humans.

Exclusion Criteria

All available data from the experimental study were included in the analyses. No data were excluded from the analysis.

Randomization

Randomization did not apply to this study since we did not test any specific therapy that required randomization.

Blinding

Investigators were blinded during the analysis of subsequent experiments after euthanasia, including histopathology and immunohistochemistry, real time quantitative polymerase chain reaction, micro-RNA analysis, quantitative high-throughput proteomics and immunoblotting studies. Blinding was not possible for operators performing *in vivo* electroanatomical mapping and ablation.
